# Advancing Lithium–Oxygen Batteries: Pioneering Cathode Catalyst Innovation and Artificial Intelligence‐Driven Design Paradigms

**DOI:** 10.1002/adma.73460

**Published:** 2026-06-01

**Authors:** Yuqing Yao, Chongyang Hao, Karol Viviana Mejia‐Centeno, Malik Dilshad Khan, Shang Wang, Longqiu Li, Yanhong Tian, Andreu Cabot, Qing Sun

**Affiliations:** ^1^ State Key Laboratory of Precision Welding & Joining of Materials and Structures Harbin Institute of Technology Harbin China; ^2^ Zhengzhou Advanced Research Institute Harbin Institute of Technology Zhengzhou China; ^3^ Catalonia Institute for Energy Research (IREC) Sant Adrià de Besòs Catalonia Spain; ^4^ Department of Electronic and Biomedical Engineering Universitat de Barcelona Barcelona Spain; ^5^ School of Materials Science and Engineering Zhejiang University Hangzhou China; ^6^ Faculty of Chemistry Universitat de Barcelona Barcelona Spain; ^7^ State Key Laboratory of Robotics and Systems Harbin Institute of Technology Harbin China; ^8^ ICREA Pg. Lluis Companys Barcelona Spain

**Keywords:** artificial intelligence, catalyst, high throughput, lithium–oxygen batteries, machine learning, oxygen evolution reaction, oxygen reduction reaction

## Abstract

Lithium‐oxygen batteries (LOBs) are regarded as one of the most promising next‐generation energy storage systems, owing to their exceptionally high theoretical energy density. However, their practical application remains severely hindered by sluggish oxygen reduction and evolution reaction kinetics, insulating discharge products, large voltage polarization, and poor cycling stability. Cathode catalysts play a pivotal role in regulating reaction pathways, accelerating interfacial kinetics, and improving reaction reversibility. This review systematically summarizes the fundamental working principles and key challenges of nonaqueous LOBs, followed by a comprehensive overview of recent advances in cathode catalyst materials, including carbon‐based catalysts, noble metals, transition metal oxides, sulfides, nitrides, carbides, and redox mediators. Particular emphasis is placed on emerging catalyst design strategies, such as single‐atom catalysts, metal–organic frameworks‐derived materials, heterostructure and interfacial engineering, high‐entropy catalysts, and spin‐related oxygen electrocatalysis. In addition, recent progress in high‐throughput computation and artificial intelligence‐driven catalyst design is explored, highlighting the potential for data‐assisted discovery, multiscale structure‐activity relationships, and mechanism‐guided optimization. The review concludes by addressing the critical challenges and outlining future directions for the development of efficient, durable, and practically viable cathode catalysts for nonaqueous LOBs.

## Graphical abstract

This review summarizes the principles and challenges of nonaqueous lithium‐oxygen batteries and recent advances in cathode catalysts, including carbon‐based materials, metals, oxides, sulfides, nitrides, carbides, and redox mediators. It highlights emerging design strategies and artificial intelligence‐driven approaches, emphasizing data‐assisted discovery, structure‐activity relationships, and future directions for efficient, durable, practical catalysts.

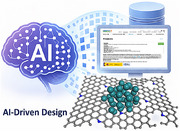

## 1 Introduction

The global energy landscape remains predominantly reliant on fossil fuels, with primary sources such as coal, oil, and natural gas accounting for over 70% of total consumption [1, 2, 3]. The extensive use of fossil energy has been a significant driver of industrialization and modernization, yet it has concurrently precipitated severe environmental degradation and greenhouse gas emissions [4, 5, 6]. Furthermore, due to the finite nature of these resources, their continued exploitation has triggered crises related to resource depletion and energy security [7, 8, 9]. In response to the urgent challenges posed by global climate change and the need for sustainable energy development, renewable clean energy sources, such as solar, wind, and tidal energy, have swiftly emerged as viable alternatives to fossil fuels [10, 11, 12]. However, the output of these energy sources is highly contingent upon geographical location, seasonal variation, and climatic conditions, resulting in substantial intermittency and fluctuations, thereby complicating the attainment of a stable and continuous energy supply [13, 14]. As a result, the advancement of high‐energy‐density, high‐efficiency, and scalable electrochemical energy storage technologies, including lithium‐ion batteries (LIBs), has become a critical strategy [15, 16]. These technologies play a key role in optimizing the utilization of renewable energy and facilitating the transformation of the global energy infrastructure.

As reversible electrochemical energy conversion devices, secondary batteries have found widespread applications in portable electronic devices, electric vehicles, and grid‐scale energy storage systems [17, 18], owing to their superior energy conversion efficiency, rapid response times, and exceptional adaptability [19]. Among various secondary battery technologies, lithium metal batteries (LMBs) have garnered significant attention due to their extraordinary theoretical capacity of 3860 mAh g^−1^ and a remarkably low electrochemical potential of ‐3.04 V relative to the standard hydrogen electrode [20, 21, 22]. The concept of lithium‐oxygen batteries (LOBs), a representative system of LMBs, was revived in 2006 and is now recognized as one of the most promising next‐generation high‐energy‐density electrochemical energy storage technologies, with an extremely high theoretical energy density of approximately 3500 Wh kg^−1^ [23]. In comparison to conventional LIBs, which typically offer energy densities below 300 Wh kg^−1^ [24, 25], LOBs demonstrate an order‐of‐magnitude improvement in energy output, not only offering significant potential for long‐range electric transportation and large‐scale energy storage, but also constituting a critical direction for realizing high‐specific‐energy electrochemical systems [26].

After decades of rigorous research, LOBs have made substantial advancements in areas such as reaction mechanisms, electrode design, and electrolyte systems [27, 28]. However, unlike conventional intercalation‐based systems, LOBs operate through coupled oxygen reduction reactions (ORR) and oxygen evolution reactions (OER), involving multistep electron transfer, soluble and insoluble intermediates, and dynamic phase transformations [29, 30]. The key bottleneck does not merely lie in insufficient catalytic activity, but rather in the lack of precise control over reaction pathways, intermediate evolution, and interfacial processes [31, 32]. In particular, the formation and transformation of LiO_2_ intermediates, the nucleation and growth of insulating Li_2_O_2_, and the generation of reactive oxygen species (especially singlet oxygen, ^1^O_2_) collectively govern the reversibility and stability of the system. Additionally, the discharge product, Li_2_O_2_, is insoluble in organic electrolytes and exhibits poor electrical conductivity [33]. It readily accumulates on the electrode surface, forming an insulating layer that obstructs electron and ion transport, thereby contributing to rapid capacity degradation. Moreover, side reactions produce carbonates and peroxides that cause electrolyte decomposition and structural degradation of the electrodes [34], severely compromising cycling stability. As a result, LOBs remain confined to the laboratory stage, with a significant gap still existing between their current performance and the requirements for commercial applications [35].

Among the core components of LOBs, the cathode catalyst plays a decisive role in governing reaction kinetics, energy efficiency, and cycle life [36]. Beyond simply lowering overpotentials, catalysts actively regulate intermediate adsorption/desorption, discharge‐product evolution, and reaction selectivity [37, 38]. Currently, the most extensively investigated catalyst types include carbon‐based materials, transition metal (TM) compounds such as oxides, sulfides, nitrides, and carbides, as well as noble metals and their composite systems. Carbon materials, including graphene, carbon nanotubes (CNTs), and porous carbon, are widely employed in electrode materials due to their high electrical conductivity and large specific surface area [39, 40], while they are prone to oxidation under high operating potentials. TM oxides (TMOs) and their derivatives feature tunable electronic structures and abundant active sites, enabling effective reduction of reaction overpotentials. However, they still face challenges such as low catalytic efficiency, poor stability, low conductivity, slow reaction kinetics, high cost, and selectivity issues. Noble metals, such as Pt, Ru, and RuO_2_, exhibit outstanding bifunctional catalytic performance [41, 42], yet their high cost and limited stability significantly restrict practical application.

Nevertheless, existing studies and reviews have predominantly focused on material classification and performance comparison, lacking a unified framework that systematically integrates catalytic behavior from a mechanistic perspective [43, 44]. To address these challenges, this review systematically analyzes the key catalytic processes in nonaqueous LOBs. Specifically, five fundamental mechanistic aspects are discussed: (i) nucleation mechanisms of Li_2_O_2_ discharge products; [45, 46, 47] (ii) growth and phase evolution of discharge products; (iii) regulation of LiO_2_ superoxide intermediates; [48, 49] (iv) electrochemical activation of insulating Li_2_O_2_; and (v) reaction selectivity and suppression of side reactions [50, 51]. Building upon these mechanistic insights, we further correlate them with catalyst design strategies, including single‐atom catalysts, MOF‐derived structures, interface engineering, and high‐entropy systems, thereby constructing a comprehensive framework that links “mechanistic regulation‐structural design‐performance enhancement” [52, 53]. Through this integrative approach, the intrinsic relationship between catalytic behavior and the overall reaction network is elucidated, providing clear mechanistic guidance for rational catalyst design.

Furthermore, the incorporation of in situ and quasi‐in situ characterization techniques, such as in situ X‐ray diffraction (XRD), X‐ray absorption spectroscopy (XAS), Raman spectroscopy, and transmission electron microscopy (TEM), alongside multiscale theoretical simulations, has facilitated a deeper understanding of the atomic‐level mechanisms of evolution reactions [54, 55, 56, 57, 58, 59]. particularly the evolution of interfacial processes. This synergy of experimental techniques and computational tools provides invaluable insights into the fundamental behavior of catalysts, offering critical scientific guidance for the rational design and optimization of next‐generation cathode catalysts for LOBs.

With the continuous advancements in catalyst design, the introduction of emerging technologies, such as high‐throughput (HT) computational screening and artificial intelligence (AI)‐driven methods, has revolutionized the discovery and optimization of catalysts [60, 61, 62]. AI, combined with big data and machine learning (ML) algorithms, has enabled more efficient screening and prediction of catalyst properties, accelerating the identification of high‐performing materials [63]. The combination of these advanced methodologies, particularly AI‐enhanced catalyst design, holds immense promise for accelerating the development of efficient, durable, and commercially viable catalysts for high‐energy‐density electrochemical energy storage systems.

This review systematically integrates research progress spanning from conventional catalytic materials to emerging intelligent design strategies, and establishes a clear academic framework linking fundamental mechanisms, materials innovation, methodological advances, and application prospects. This unified perspective not only facilitates a comprehensive understanding of the intrinsic roles of different catalytic systems in LOBs, but also provides a solid theoretical foundation for the transition from empirically guided optimization to mechanism‐driven design. Furthermore, by incorporating emerging data‐driven and artificial intelligence approaches, this work expands the scope and pathways of rational catalyst design, offering forward‐looking guidance for the development of efficient, stable, and practically viable LOB systems.

## 2 Development and Working Principles of LOBs

The concept of LOBs can be traced back to 1976, when E. L. Littauer and K. C. Tsai first proposed a metal‐air battery system employing metallic lithium as the anode and oxygen as the cathode reactant [64], marking the emergence of the prototype lithium‐oxygen electrochemical energy storage system. This system initially adopted aqueous electrolytes, in which the charge‐discharge reactions were based on the reversible conversion between LiO_2_ and LiOH. However, metallic lithium undergoes severe parasitic reactions in aqueous electrolytes [65], producing hydrogen gas and causing electrode corrosion, which not only significantly reduces energy efficiency but also introduces serious safety hazards and self‐discharge issues. Owing to the intrinsic instability of aqueous systems, such early LOBs failed to receive sustained in‐depth investigation for an extended period, and their development was consequently stalled.

### 2.1 Development of LOBs

It was not until 1996 that K. M. Abraham and Z. Jiang successfully designed and assembled a non‐aqueous LOB in the laboratory [66], marking a breakthrough in the development of this system. In their design, metallic lithium was employed as the anode, a lithium‐ion (Li^+^)‐conducting solid electrolyte membrane was used to replace the conventional separator, and high‐specific‐surface‐area carbon materials served as the cathode catalytic host, thereby constructing a non‐aqueous battery system capable of stable operation in organic electrolytes. This innovative configuration effectively avoided parasitic reactions between metallic lithium and water, significantly enhancing the reversibility and cycling stability of the battery. Experimental results demonstrated that the battery could deliver a discharge specific capacity as high as 1400 mAh g^−1^ [67], thereby validating for the first time the feasibility of LOBs as rechargeable energy storage systems and laying the foundation for subsequent rapid development in this field.

Thereafter, research on LOBs entered a systematic and mechanism‐oriented stage, with research institutions worldwide successively conducting in‐depth investigations into electrode materials, electrolytes, and reaction mechanisms. In 2002, Read et al. [68] reported the significant influence of electrolyte solvent properties and air electrode structures on electrochemical performance, demonstrating that the solvation ability, viscosity, and stability of the electrolyte directly determine discharge product morphology and battery reversibility, thereby providing important guidance for subsequent electrolyte design. In 2006, the Bruce group first verified lithium peroxide (Li_2_O_2_) as the discharge product [69], using in situ differential electrochemical mass spectrometry, and revealed that it could be electrochemically oxidized and decomposed into oxygen during charging, enabling relatively stable charge‐discharge cycling of approximately 50 cycles. This milestone discovery established for the first time the reversible redox mechanism of Li_2_O_2_ as the primary discharge product, fundamentally elucidating the energy storage and release mechanism of LOBs.

**FIGURE 1:**
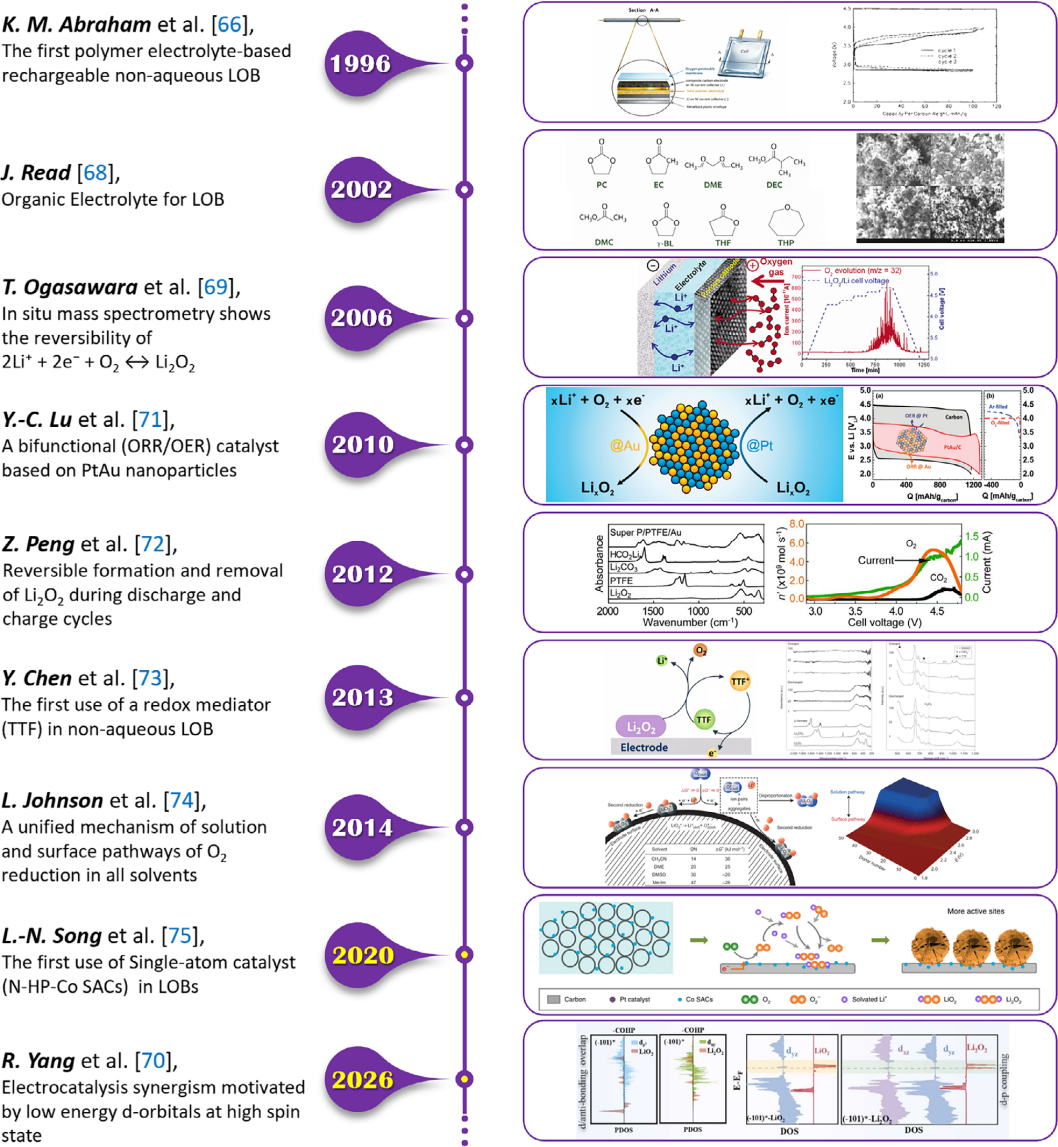
The Development History and Major Milestones of LOBs. Reproduced with permission [66]. Copyright 1996, IOP Publishing. Reproduced with permission [68]. Copyright 2002, IOP Publishing. Reproduced with permission [69]. Copyright 2006, American Chemical Society. Reproduced with permission [71]. Copyright 2010, American Chemical Society. Reproduced with permission [72]. Copyright 2012, American Association for the Advancement of Science. Reproduced with permission [73]. Copyright 2013, Springer Nature. Reproduced with permission [74]. Copyright 2014, Springer Nature. Reproduced with permission [75]. Copyright 2020, Springer Nature. Reproduced with permission [70]. Copyright 2026, Elsevier.

Since then, pioneering studies have greatly stimulated international academic interest in LOBs, driving a series of breakthrough advances in electrochemical mechanism elucidation, electrode design, catalyst development, and electrolyte optimization, as illustrated in Figure 1 [66, 68, 69, 70, 71, 72, 73, 74, 75]. With the advancement of in situ and quasi‐in situ characterization techniques and multiscale theoretical simulations [76, 77], the complex reaction processes and evolution pathways of intermediate species in LOBs have been more clearly revealed, enabling this system to transition from an early exploratory stage toward a mechanism‐guided rational design paradigm. At present, LOBs have become one of the major research focuses in next‐generation high‐energy‐density electrochemical energy storage systems, providing important scientific foundations and technological pathways for overcoming the energy density limitations of conventional LIBs.

### 2.2 Working Principles of LOBs

**FIGURE 2:**
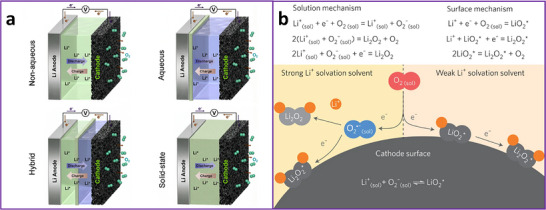
(a) Schematic configuration of all types of LOBs. Reproduced with permission [81]. Copyright 2026, Elsevier. (b) Reduction mechanisms in LOBs at low overpotentials [82]. Copyright 2026, Springer Nature.

The fundamental working principles of LOBs rely on the reversible reduction and evolution of oxygen. According to the type and physical state of the electrolyte, LOBs can be classified into four categories: i) non‐aqueous [72]; ii) aqueous [78]; iii) solid‐state [79]; and iv) hybrid systems [80], as illustrated in Figure 2a [81].

Among the various configurations, rechargeable non‐aqueous LOBs have attracted extensive attention over the past decades due to their high energy density and reversibility [83, 84, 85], and have gradually become the dominant research focus of LOBs. In rechargeable non‐aqueous systems, a typical LOB consists of a lithium metal anode, a porous oxygen cathode, and an electrolyte, with pure oxygen serving as the reactant gas. The fundamental electrochemical processes include lithium stripping and deposition at the anode, as well as ORR and OER at the cathode. Taking the non‐aqueous system as an example, during discharge, metallic lithium is oxidized at the anode to generate Li^+^, which subsequently migrates through the electrolyte to the cathode [86]. The corresponding anodic electrochemical reaction is expressed as:

(1) $$\mathrm{Li}\to Li^{+}+e^{-}$$

At the air cathode, oxygen molecules (O_2_) are reduced to superoxide anions (O_2_^−^) under the catalytic effect of the cathode catalyst. The superoxide anions react with Li^+^ at the electrolyte/oxygen/catalyst three‐phase interface to form LiO_2_ intermediates. Owing to the chemical instability of LiO_2_, the discharge product Li_2_O_2_ is typically formed via disproportionation or further electrochemical reduction [87]. Accordingly, the overall cathodic reaction during discharge can be summarized as:

(2) $$2Li^{+}+O_{2}+2e^{-}\to Li_{2}O_{2}$$

During charging, Li_2_O_2_ is oxidized and decomposed under an applied potential and the action of the catalyst, regenerating oxygen and driving Li^+^ back to the anode to form metallic lithium [88]. The entire redox process occurs at the cathode, while Li^+^ migrates through the electrolyte to maintain charge balance and complete the electrochemical cycle.

However, the solubility of oxygen in non‐aqueous solvents is extremely low, and the discharge product Li_2_O_2_ is an electrically insulating solid [89]. Li_2_O_2_ typically deposits on the electrode surface in the form of thin films or toroidal crystalline particles. As discharge proceeds, the accumulation of Li_2_O_2_ blocks cathode pores and covers catalytic active sites, leading to restricted oxygen diffusion, increased electrode polarization, and ultimately capacity fading and degraded cycling performance. Studies have shown that the discharge capacity is strongly influenced by the deposition morphology and particle size of Li_2_O_2_, as well as the pore structure of the electrode [90, 91]. In addition, both LiO_2_ intermediates and Li_2_O_2_ possess strong oxidizing properties and readily undergo side reactions with electrolyte solvents and carbon supports. These side reactions generate irreversible carbonate or ether‐based products, further deteriorating the energy efficiency and lifespan of LOBs.

The reaction behavior of batteries varies significantly across different electrolyte systems [92, 93]. In aqueous batteries, the discharge products are typically H_2_O or LiOH, and although the theoretical discharge voltage is relatively high, severe self‐reduction reactions between metallic lithium and the aqueous electrolyte lead to substantial safety risks and energy losses [78]. In hybrid aqueous and non‐aqueous systems, organic and aqueous electrolytes are employed on the lithium metal anode and oxygen cathode sides, respectively, with a solid electrolyte membrane serving as both the ionic conductor and physical separator [94]. This configuration partially protects the lithium metal anode and mitigates excessive accumulation of Li_2_O_2_ at the cathode. However, the solid electrolyte layer exhibits insufficient chemical stability under strongly acidic or alkaline conditions, and the diffusion kinetics of Li^+^ in multiphase media are limited, resulting in increased battery polarization [80].

Although solid‐state LOBs can effectively suppress electrolyte decomposition and lithium anode corrosion, their discharge capacity and rate capability remain markedly inferior to those of liquid systems due to the relatively low ionic conductivity of solid electrolytes [95]. Consequently, current research efforts are primarily focused on rechargeable non‐aqueous LOBs, which employ proton‐inert organic electrolyte systems and achieve a more favorable balance among energy density, reversibility, and cycling stability.

In recent years, with the advancement of in situ characterization and theoretical modeling, it has been increasingly recognized that the discharge mechanisms of non‐aqueous LOBs mainly involve two modes: i) the solution‐mediated mechanism and ii) the surface‐mediated mechanism (Figure 2b) [82]. The dominant pathways of these mechanisms are closely related to the donor number (DN) of the electrolyte solvent, lithium salt chemistry, oxygen solubility, and solvent‐ion coordination structures.

In the solution‐mediated mechanism, LiO_2_ intermediates can dissolve into the electrolyte and subsequently form toroidal Li_2_O_2_ particles in the solution phase via disproportionation reactions. This mechanism enables relatively high discharge capacities. However, the large size of the resulting particles impedes oxygen diffusion, leading to a relatively short cycle life. The primary reaction pathways can be described as:

(3)

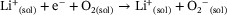



(4)

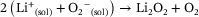



(5)

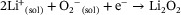




In contrast, in the surface‐mediated mechanism, Li_2_O_2_ is generated directly on the electrode surface in the form of thin films [96, 97]. This process is constrained by electrode conductivity and the distribution of active sites. Despite delivering a lower discharge capacity, the abundance of defect structures in the film endows the system with relatively superior charge‐discharge reversibility. The primary surface‐mediated reaction step can be expressed as:

(6) $$Li^{+}+e^{-}+O_{2(\mathrm{sol})}\to \mathrm{Li}{O_{2}}^{*}$$

(7) $$Li^{+}+e^{-}+\mathrm{Li}{O_{2}}^{*}\to Li_{2}{O_{2}}^{*}$$

(8) $$\mathrm{Li}{O_{2}}^{*}\to Li_{2}{O_{2}}^{*}+O_{2}$$

During charging, Li_2_O_2_ undergoes stepwise OER, with amorphous phases oxidized at ∼3.2 V and crystalline phases decomposed at ∼4.5 V [98].

Furthermore, recent studies have revealed that highly reactive oxygen species, particularly singlet oxygen (^1^O_2_), play a critical role in nonaqueous LOBs. ^1^O_2_ can be generated through multiple pathways, including the disproportionation of LiO_2_ intermediates, electrochemical oxidation of Li_2_O_2_ during charging, and oxygen evolution processes at high potentials. Compared with the ground‐state triplet oxygen (^3^O_2_), ^1^O_2_ exhibits much higher chemical reactivity and can readily react with electrolyte components, carbon electrodes, and other organic species [99, 100].

The formation of ^1^O_2_ is widely recognized as a key contributor to electrolyte decomposition and the generation of parasitic byproducts such as Li_2_CO_3_ and ROCO_2_Li. These species are difficult to decompose during charging and tend to accumulate on the electrode surface, blocking active sites and pore structures, thereby increasing polarization and reducing reversibility. Moreover, ^1^O_2_ can induce interfacial degradation and accelerate catalyst deactivation, ultimately leading to rapid capacity fading [101]. Therefore, suppressing the generation of ^1^O_2_ or developing effective quenching strategies has become a crucial direction for improving the cycling stability of LOBs.

The working principles of LOBs are governed by the reversible ORR/OER processes, involving complex multiphase and multistep reaction pathways. Among various configurations, non‐aqueous LOBs have emerged as the most intensively studied system due to their favorable balance between energy density and reversibility. However, their performance is intrinsically limited by the insulating nature of Li_2_O_2_, sluggish reaction kinetics, and the intricate interplay between solution‐mediated and surface‐mediated mechanisms. In addition, the generation of highly reactive oxygen species, particularly singlet oxygen (^1^O_2_), further exacerbates parasitic reactions and interfacial degradation, posing critical challenges to cycling stability and energy efficiency. Therefore, a comprehensive understanding of reaction pathways, intermediate evolution, and interfacial processes is essential for guiding catalyst design and system optimization. Such insights provide a fundamental basis for developing advanced LOB systems with improved reversibility, stability, and practical applicability.

### 2.3 Challenges of LOBs

Despite the ultrahigh theoretical energy density of nonaqueous LOBs, their complex reaction mechanisms and multiphase interfacial processes give rise to several critical challenges that severely hinder practical applications [102]. In general, these issues can be summarized into four key aspects:

i) high charge‐discharge overpotential and low energy conversion efficiency. Although the theoretical potential of LOBs is 2.96 V [103], a large voltage gap between charge and discharge is commonly observed in practice, resulting in poor energy efficiency [101]. The primary origin lies in the insulating nature of the discharge product Li_2_O_2_ (conductivity ∼10^−14^ S cm^−1^), which deposits on the electrode surface, blocks electron/ion transport, and passivates active sites. This leads to sluggish reaction kinetics and increased polarization. Therefore, constructing highly conductive porous electrodes, introducing bifunctional catalysts, and employing soluble redox mediators to facilitate the reversible formation and decomposition of Li_2_O_2_ are key strategies to reduce overpotential.

ii) Limited rate capability. The electrochemical reactions in LOBs occur at the gas‐liquid‐solid three‐phase interface, where mass transport and reaction kinetics are inherently slow. Under high current densities, rapid Li_2_O_2_ deposition leads to electrode passivation and a sharp increase in impedance, thereby restricting rate performance [104]. Effective approaches include regulating discharge pathways (surface versus solution‐mediated routes), designing catalysts that enable controlled product growth, and optimizing electrode porosity and oxygen transport [105].

iii) Poor cycle life. Highly reactive oxygen intermediates, such as O_2_^−^ and singlet oxygen (^1^O_2_), can induce electrolyte decomposition and carbon corrosion, generating irreversible byproducts (e.g., Li_2_CO_3_) that cause capacity decay [106]. Meanwhile, incomplete decomposition of Li_2_O_2_ during charging results in its gradual accumulation, clogging electrode pores, increasing charging voltage, and further aggravating side reactions [107]. Accordingly, improving electrolyte stability, suppressing the formation of reactive oxygen species (especially ^1^O_2_), and developing robust non‐carbon electrodes are crucial for enhancing cycling performance.

iv) Safety issues associated with the lithium metal anode. Lithium metal is highly reactive and readily reacts with trace gases or moisture [108]. During cycling, dendrite growth may occur, posing risks of internal short circuits. In addition, volume fluctuations of lithium can disrupt the solid electrolyte interphase (SEI), leading to continuous electrolyte consumption and interfacial instability [109]. Common strategies to address these issues include constructing artificial protective layers, optimizing SEI chemistry, and employing solid‐state electrolytes [110].

The fundamental challenges of LOBs can be attributed to sluggish reaction kinetics, unstable interfaces, and severe side reactions. Future efforts should focus on the synergistic optimization of catalysts, electrolytes, and electrode architectures to achieve simultaneous improvements in energy efficiency, cycling stability, and safety.

### 2.4 Key Issues of LOBs

**FIGURE 3:**
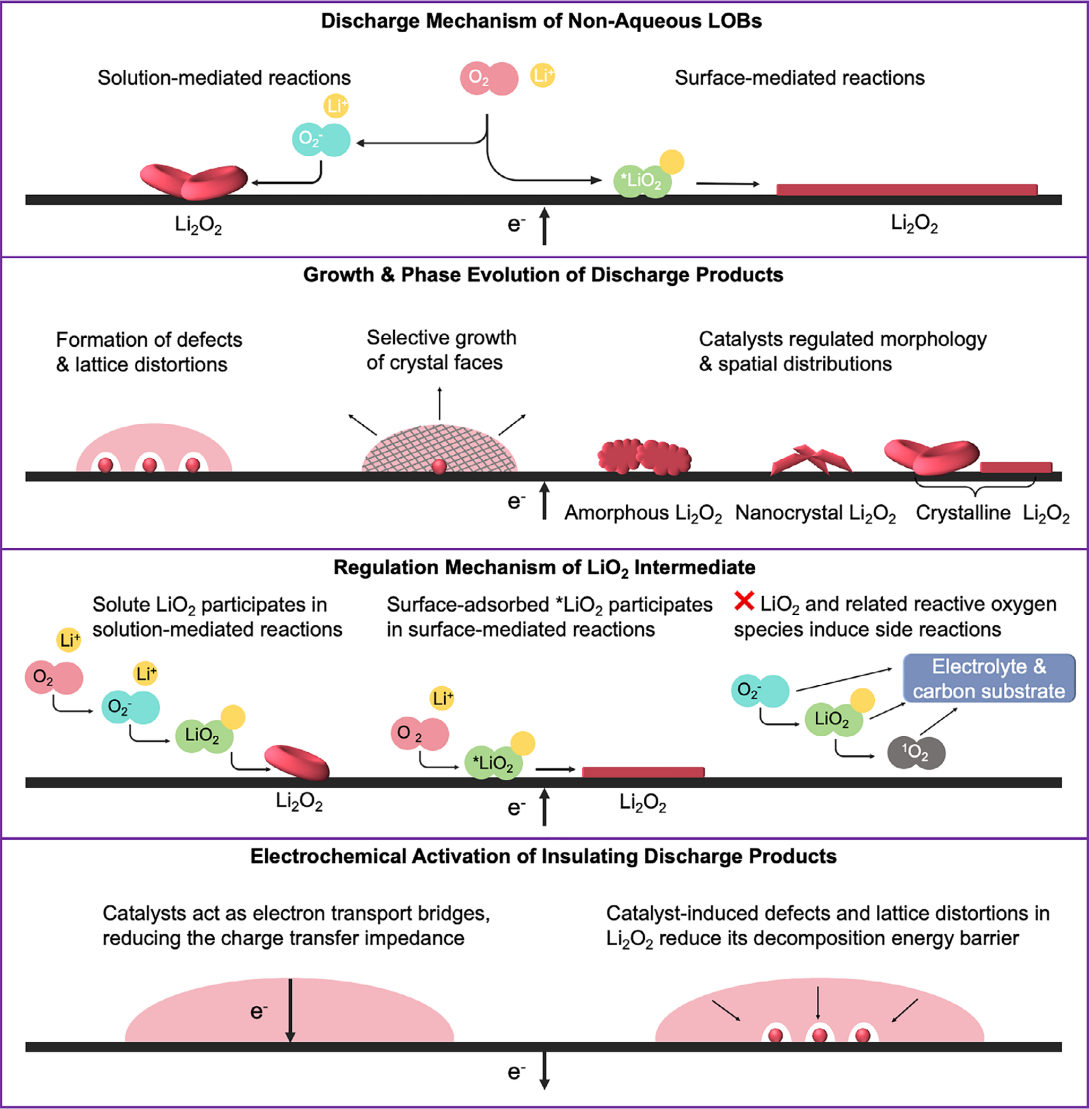
Key catalytic mechanisms for catalyst regulation of discharge intermediates and discharge products in non‐aqueous LOBs.

The core of performance improvement in LOBs lies in accelerating the catalytic processes of ORR and OER. Introducing efficient cathode catalysts is widely regarded as a key strategy for improving LOBs performance. However, from a mechanistic perspective, catalysis in LOBs does not simply lower overpotential, but deeply participates in and regulates reaction‐pathway selection, intermediate behavior, discharge‐product evolution, and the occurrence and suppression of side reactions [111]. Therefore, a fundamental understanding of catalytic processes has become a central focus of current research. Based on existing studies, the key catalytic mechanistic issues in nonaqueous LOBs can be summarized into five interrelated yet scientifically distinct aspects (Figure 3): i) Nucleation Mechanisms of Li_2_O_2_ Discharge Product; ii) Growth and Phase‐Evolution Mechanisms of Discharge Products; iii) Regulation Mechanisms of Superoxide Intermediate LiO_2_; iv) Electrochemical Activation Mechanisms of Insulating Discharge Product; and v) Reaction Selectivity and Side‐Reaction Suppression Mechanisms.

#### 2.4.1 Nucleation Mechanisms of Li_2_O_2_ Discharge Product

In nonaqueous LOBs, the initial nucleation behavior of Li_2_O_2_ determines the subsequent evolution of the discharge process and serves as a critical starting point governing capacity utilization and electrode stability [112]. Fundamentally, nucleation is governed by the interplay of interfacial thermodynamics and kinetics, with the associated energy barrier closely dependent on surface energy, local supersaturation, and the interfacial chemical environment. At this stage, catalysts primarily function by regulating the initial conditions of nucleation. On one hand, the abundant and tunable active sites on catalyst surfaces can significantly lower the heterogeneous nucleation barrier of Li_2_O_2_, thereby increasing nucleation density and altering its spatial distribution. On the other hand, differences in wettability between reaction intermediates, products, and catalyst surfaces further influence the nucleation size and its spatial characteristics [113]. Notably, nucleation behavior determines not only whether Li_2_O_2_ forms, but more critically its initial phase state. Existing studies indicate that in certain catalytic systems, Li_2_O_2_ may preferentially nucleate in amorphous or defect‐rich states rather than directly forming highly ordered crystalline structures [114]. Such differences in initial phase state can profoundly affect subsequent electron‐transport properties and reversible decomposition behavior. Therefore, from a mechanistic viewpoint, catalysts effectively reshape the initial conditions of the entire discharge reaction by regulating nucleation during the early discharge stage.

#### 2.4.2 Growth and Phase‐Evolution Mechanisms of Discharge Products

Following initial nucleation, continuous formation of Li_2_O_2_ is accompanied by complex growth and phase‐evolution processes [115]. Unlike conventional battery systems, discharge products in LOBs do not deposit uniformly, but instead exhibit highly diverse morphologies and structural characteristics. At this stage, catalysts mainly exert influence by regulating Li_2_O_2_ growth kinetics and crystal‐evolution pathways. Specifically, selective adsorption of catalysts on certain Li_2_O_2_ crystal facets can induce anisotropic growth, resulting in flake‐like or spherical structures. In addition, catalyst surfaces may induce defect formation, lattice distortion, or amorphous regions in Li_2_O_2_ [116], significantly altering its electronic and ionic transport properties. Meanwhile, the spatial distribution of discharge products is also strongly influenced by catalysts. Rationally designed catalytic systems can promote uniform Li_2_O_2_ deposition within electrode pores, avoiding localized blockage and premature electrode failure. Mechanistically, discharge‐product growth and phase evolution are not simple physical deposition processes, but dynamic evolutions highly coupled with interfacial chemistry, charge transport, and intermediate behavior.

#### 2.4.3 Regulation Mechanisms of Superoxide Intermediate LiO_2_

During discharge in LOBs, LiO_2_ is generally considered a key superoxide intermediate linking O_2_ reduction and Li_2_O_2_ formation [117]. LiO_2_ exhibits strong radical character and extremely high reactivity, and its behavior at the electrode interface directly determines reaction‐pathway selection. Mechanistically, LiO_2_ faces three possible evolutionary pathways: i) LiO_2_ can be further reduced on the electrode surface to form Li_2_O_2_; ii) LiO_2_ can desorb from the electrode surface and enter the solution phase, participating in solution‐mediated reactions; and iii) LiO_2_ can induce side reactions, such as attacking electrolyte molecules. The core role of catalysts is to regulate the adsorption‐stabilization‐desorption balance of LiO_2_ on the electrode surface. Strong adsorption of LiO_2_ by catalysts favors surface‐mediated discharge mechanisms, whereas weaker adsorption promotes LiO_2_ desorption into solution and enhances solution‐mediated pathways. This regulation mechanism is regarded as one of the fundamental causes of differences in Li_2_O_2_ morphology, such as film‐like versus particulate structures [118]. In addition, the stability of LiO_2_ is closely related to its electronic structure, and different catalysts can modify its disproportionation kinetics by regulating charge distribution. Therefore, regulation of LiO_2_ intermediates is not only key to understanding ORR pathway selection, but also a central scientific issue in constructing highly reversible LOBs systems.

#### 2.4.4 Electrochemical Activation Mechanisms of Insulating Discharge Products

The strong electronic insulating nature of Li_2_O_2_ is widely recognized as a fundamental cause of high charging overpotential and low energy efficiency in LOBs [119]. Consequently, achieving efficient and reversible decomposition of Li_2_O_2_ is a core mechanistic issue in catalytic research. During charging, oxidation of Li_2_O_2_ is not a simple reverse reaction, but often involves electron extraction from insulating phases and formation of defects or intermediate states. Catalysts can promote this “electrochemical activation” through multiple pathways: i) On one hand, catalysts serve as electron‐transport bridges, reducing charge‐transfer resistance at the Li_2_O_2_/electrode interface; ii) On the other hand, catalysts may induce oxygen vacancies or nonstoichiometric structures in Li_2_O_2_, thereby effectively lowering its decomposition energy barrier. The essence of this mechanism lies in enabling an intrinsically insulating discharge product to participate in reversible electrochemical reactions [120]. This issue directly affects charge‐discharge overpotential and ultimately determines whether long‐term stable cycling can be achieved.

#### 2.4.5 Reaction Selectivity and Side‐Reaction Suppression Mechanisms

In addition to the main reactions, LOB systems commonly suffer from side reactions such as electrolyte decomposition, carbon‐support corrosion, and attack by reactive oxygen species. These side reactions are often closely related to the presence of catalysts, constituting a “double‐edged sword” problem in catalytic systems [121]. Mechanistically, some catalysts may promote radical reactions or Fenton‐like processes while accelerating ORR/OER, thereby intensifying electrolyte degradation. Moreover, reactive oxygen species generated during charging, especially singlet oxygen(^1^O_2_), are widely regarded as major triggers of side reactions. Therefore, efficient cathode catalysts must not only exhibit high catalytic activity, but also possess good reaction selectivity by promoting desired reactions while suppressing undesired side reactions [122]. Such selectivity fundamentally depends on the electronic structure, acid‐base properties, and ability to regulate reactive oxygen species on catalyst surfaces. From this perspective, catalytic research in LOBs has gradually shifted from merely “increasing reaction rates” toward fine regulation of complex reaction networks.

Therefore, the design of LOBs cathode catalysts must be grounded in systematic and in‐depth mechanistic understanding. Only by synergistically regulating discharge‐product nucleation, superoxide‐intermediate behavior, product growth and phase evolution, electrochemical activation of insulating products, and reaction selectivity can efficient, stable, and practically viable LOBs systems be realized.

## 3 Research Progress on Cathode Catalyst Materials for LOBs

In non‐aqueous electrolyte systems, the primary discharge product of LOBs is Li_2_O_2_, and the chemical properties and morphology of the discharge product exert a significant influence on cycling performance. With continuous deposition of Li_2_O_2_ during discharge or incomplete decomposition during charging, oxygen transport pathways are progressively blocked, while the electrically insulating nature of the product induces electrode polarization, ultimately leading to rapid performance degradation. Therefore, cathode catalyst materials for LOBs are required to possess a high specific surface area and rational pore architecture to enhance discharge capacity [123], as well as good electrical conductivity and high catalytic activity toward ORR and OER to reduce overpotential.

**FIGURE 4:**
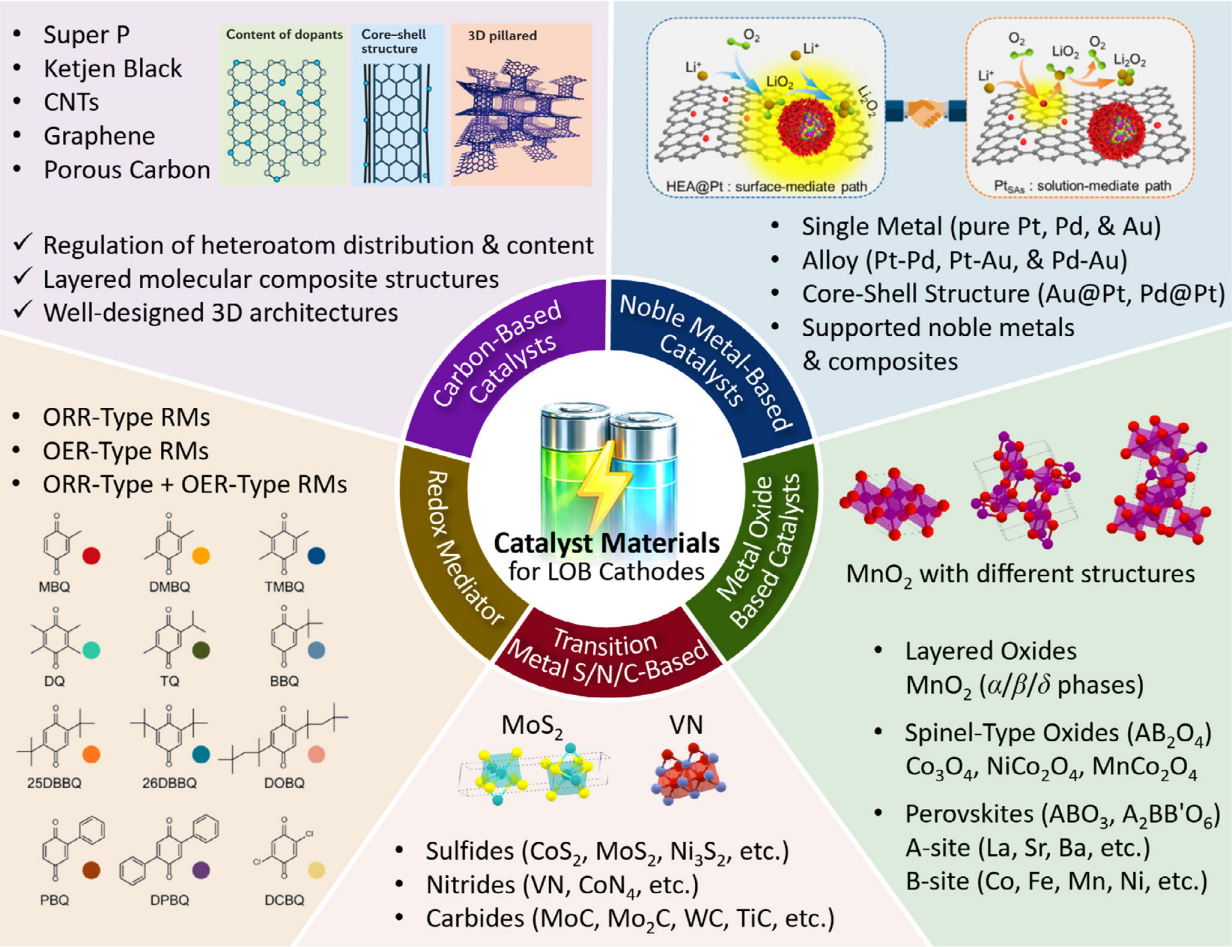
Classification of cathode catalyst materials for LOBs. Reproduced with permission [124]. Copyright 2016, American Chemical Society. Reproduced with permission [125]. Copyright 2025, Royal Society of Chemistry. Reproduced with permission [126]. Copyright 2023, Springer Nature.

In this chapter, representative research directions are systematically categorized according to catalyst composition and functional mechanisms, covering carbon‐based catalysts, noble‐metal‐based catalysts, TMOs, and TM sulfides, nitrides, and carbides. In addition, emerging strategies that regulate reaction pathways through RMs are further introduced, as illustrated in Figure 4. Through a comparative analysis of the structural characteristics, catalytic mechanisms, and performance merits and limitations of different catalytic systems, this chapter aims to delineate the developmental trajectory of cathode catalysts for LOBs, identify existing issues and challenges, and provide theoretical foundations and research insights for the subsequent design of high‐performance catalytic materials.

### 3.1 Carbon‐Based Catalysts

Carbon materials, owing to their high specific surface area, excellent electrical conductivity, good chemical stability, low cost, and engineerable 3D porous structures, have long been regarded as one of the most important functional components in electrochemical energy storage devices [127, 128]. In LOBs, these properties are particularly beneficial for promoting rapid electron and oxygen transport, enhancing the accommodation efficiency of discharge products, and maintaining mechanical stability during long‐term cycling. In addition to the widespread use of commercial carbons such as Super P [129], Ketjen Black (KB) [130], Vulcan carbon [131], and acetylene black as conductive additives [132], various carbon materials with specific structures or functionalities, including CNTs, graphene, and porous carbon frameworks, have also been continuously developed for constructing high‐performance cathodes.

In the early stages of LOBs research, carbon blacks were first employed as air electrodes due to their 3D porous structures and relatively high specific surface areas, but their capacity was limited, and their structures were prone to collapse during cycling, leading to performance degradation [133]. Subsequent studies gradually clarified that pore structure plays a critical role in oxygen diffusion, Li^+^ transport, and discharge product deposition behavior, and that rational regulation of pore volume and pore size distribution can significantly improve electrochemical performance, making pore‐structure engineering an important strategy for enhancing carbon‐based cathodes.

**FIGURE 5:**
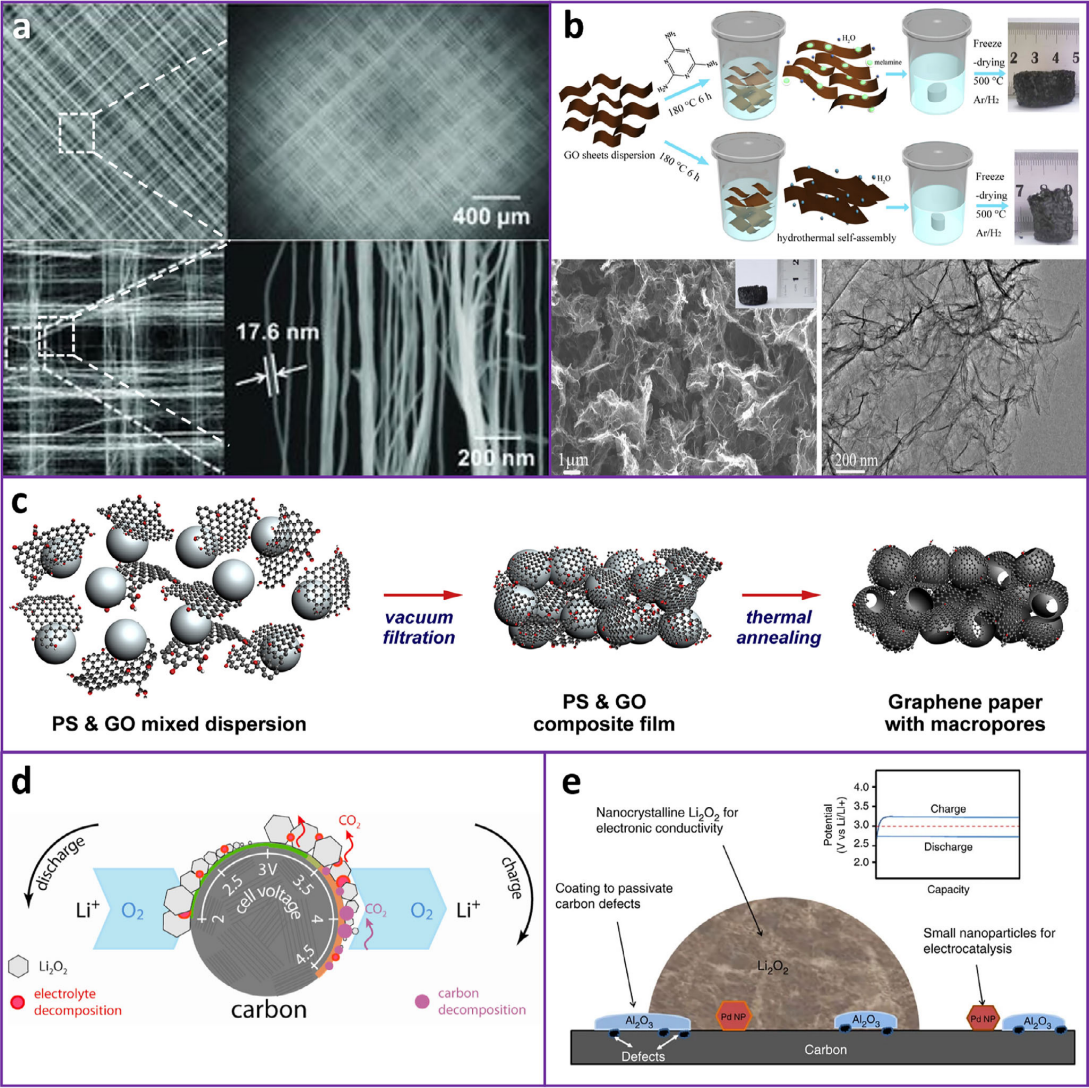
(a) SEM images of the CNT fibril at low magnification and high magnification (inset: large area image of the air electrode). Reproduced with permission [134]. Copyright 2012, Wiley. (b) Schematic illustration of the preparation processes of N‐3DG and 3DG. Reproduced with permission [136]. Copyright 2016, Elsevier. (c) Schematic illustration showing the procedure for the fabrication of the mp‐GP. Reproduced with permission [137]. Copyright 2016, Elsevier. (d) The Carbon Electrode in Nonaqueous LOBs. Reproduced with permission [140]. Copyright 2012, American Chemical Society. (e) A schematic diagram illustrating the combined effects of an alumina (Al_2_O_3_) coating, palladium nanoparticles, and nanocrystalline Li_2_O_2_ (the inset shows the hypothetical charge/discharge voltage versus capacity curve). Reproduced with permission [141]. Copyright 2013, Springer Nature.

With the development of structurally controllable carbon materials, 1D CNTs and carbon nanofibers (CNFs) have been widely applied to improve reversible capacity and cycle life due to their excellent electrical conductivity, mechanical robustness, and ability to construct continuous electron transport pathways. Ordered or highly porous carbon nanotube architectures prepared by chemical vapor deposition not only provide fast oxygen diffusion channels but also significantly increase electrocatalytically active sites, thereby facilitating ORR and OER (Figure 5a) [134]. Further optimization of the graphitization degree and surface chemical properties of CNTs enables more pronounced performance in terms of high specific capacity and high reversibility [135]. Meanwhile, graphene materials, benefiting from their ultra‐high specific surface area and outstanding electron transport capability, exhibit significant advantages in constructing 3D network electrodes. Nitrogen‐doped 3D graphene aerogels with hierarchical microporous‐mesoporous structures not only facilitate electrolyte infiltration and oxygen diffusion but also provide sufficient space for the deposition and removal of solid Li_2_O_2_ (Figure 5b) [136]. Macroporous graphene or porous carbon films prepared using monodisperse polystyrene colloidal spheres as sacrificial templates further optimize discharge product formation and decomposition pathways and reduce charge‐discharge polarization (Figure 5c) [137].

Beyond structural optimization, defect engineering and heteroatom regulation can further enhance the intrinsic catalytic activity of carbon materials. The introduction of mesopores onto carbon surfaces via steam activation, hydrothermal treatment, or alkali etching can simultaneously increase specific surface area, active site density, and structural stability, thereby effectively enhancing capacity output [138]. Heteroatom doping, such as with N, S, P, or B, modulates the electronic structure and surface chemistry of carbon, strengthens the adsorption and activation of O_2_ and key reaction intermediates, improves ORR/OER kinetics, and mitigates irreversible side reactions under high potentials [139].

Despite significant improvements in reaction kinetics and capacity performance through structural and chemical regulation, carbon‐based cathodes still suffer from insufficient stability during high‐potential charging processes. Studies have shown that when the potential exceeds approximately 3.5 V, carbon materials can react with Li_2_O_2_ to form Li_2_CO_3_ and may even undergo oxidative decomposition of the carbon framework (Figure 5d) [140]. In addition, reactive oxygen species such as superoxide radicals can attack carbon surfaces to form oxidative functional groups including hydroxyl and carbonyl species, further weakening structural stability and potentially inducing electrolyte degradation. These side reactions collectively lead to gradual capacity fading and deteriorated cycling performance, constituting a major bottleneck for the application of carbon‐based cathodes.

To alleviate these stability issues and further enhance electrochemical performance, interfacial protection and composite catalytic strategies have been extensively employed. For instance, constructing ultrathin oxide protective layers on carbon surfaces via atomic layer deposition effectively blocks direct contact between carbon and reactive species, suppresses side reactions, and simultaneously reduces charging overpotential, as illustrated in Figure 5e [141]. Surface modification using stable layers such as ZnO or loading catalytically active metal oxides [142], including CoFe_2_O_4_, NiFe_2_O_4_, NiCo_2_O_4_, and MnO_2_, onto porous carbon frameworks can also markedly enhance reaction reversibility and structural durability [143, 144, 145]. Through these strategies, the electrical conductivity and structural advantages of carbon materials can be retained while their cycling stability under complex redox environments is substantially improved, providing practically meaningful material pathways for the development of high‐performance LOBs.

### 3.2 Noble‐Metal‐Based Catalysts

**FIGURE 6:**
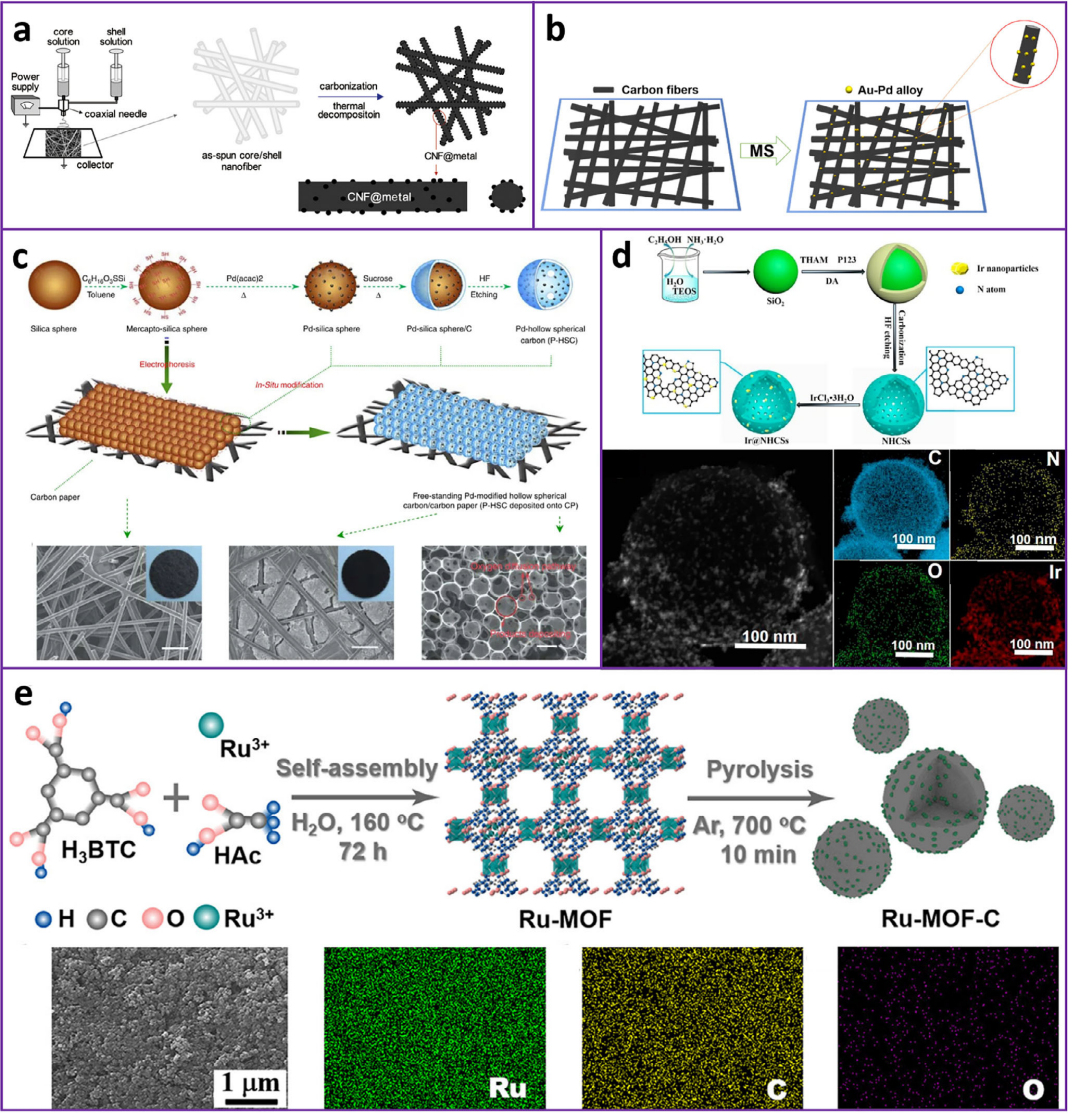
(a) Schematic of the coaxial electrospinning fabrication procedure for the CNF@metal mats. Reproduced with permission [146]. Copyright 2018, Elsevier. (b) Schematic illustration of the preparation of Au‐Pd@CNFs. Reproduced with permission [148]. Copyright 2020, Elsevier. (c) Schematic representations for the design and preparation of the palladium (Pd)‐modified hollow spherical carbon (P‐HSC) deposited onto carbon paper (CP) cathode. Reproduced with permission [152]. Copyright 2013, Springer Nature. (d) Schematic illustration and the morphologies of the synthesis procedure of NHCSs and Ir@NHCSs. Reproduced with permission [153]. Copyright 2019, Elsevier. (e) Schematic illustration of the main fabrication process of Ru‐MOF‐C, and SEM image and corresponding EDS mapping analysis of Ru‐MOF‐C. Reproduced with permission [155]. Copyright 2019, American Chemical Society.

Noble‐metal catalysts exhibit unique advantages in the ORR and OER of LOBs owing to their partially filled d orbitals, moderate adsorption energies toward reaction intermediates, and excellent electrochemical stability. Typical noble metals such as platinum (Pt) [146], palladium (Pd) [147], gold (Au) [148], ruthenium (Ru) [149, 150], and iridium (Ir) remain stable over wide potential windows and effectively reduce the energy barriers of oxygen electrochemical reactions [151]. Specifically, Pt and Pd usually display fast ORR kinetics, Ru and Ir exhibit higher catalytic activity toward OER, and Au shows outstanding stability under strongly oxidative environments. The synergistic catalytic characteristics of these materials render them core catalytic systems for enhancing the energy efficiency and reversibility of LOBs, as illustrated in Figure 6a‐b [146, 148].

When integrated into electrode architectures with high specific surface area and well‐developed conductive frameworks, the catalytic performance of noble metals can be further amplified. For example, porous Au‐based electrodes can maintain stable reaction interfaces under high‐rate conditions [72], while electrodes loaded with Ru or Ir nanostructures retain high structural integrity even at strongly oxidative potentials. These results demonstrate that through rational design of support structures, noble metals such as Pt, Pd, Au, Ru, and Ir can deliver excellent catalytic efficiency and cycling stability under harsh electrochemical conditions.

Noble metals can also markedly regulate the nucleation and growth behavior of the solid discharge product Li_2_O_2_. When noble metals are introduced into porous carbon matrices, such as Pd‐decorated hollow carbon frameworks or hierarchical carbon networks embedded with Ru or Ir [152, 153], uniform, thin, or dispersed Li_2_O_2_ morphologies are formed instead of dense insulating films typically observed on conventional carbon electrodes (Figure 6c,d). Materials such as Pd and PtRu alloys are particularly effective in promoting 2D nucleation of Li_2_O_2_, thereby significantly reducing polarization and preventing electrode passivation caused by product blockage [154]. This capability to modulate deposition pathways enables electrodes to sustain high discharge capacities and good reversibility even at high current densities.

Embedding noble‐metal nanoparticles into mesoporous or hollow carbon hosts further realizes synergistic enhancement between structure and catalysis. For instance, Ir nanoparticles composited with nitrogen‐doped hollow carbon spheres utilize internal cavities to accommodate Li_2_O_2_, mesoporous channels to enhance mass transport, and abundant exposed Ir active sites to effectively reduce charging overpotential [153]. Similarly, uniformly dispersed Ru nanoparticles within 3D hierarchical carbon frameworks significantly improve interfacial reaction activity and cycling stability, allowing electrodes to maintain low polarization and high reversibility during prolonged cycling [149].

In terms of interfacial chemical stability, noble metals also play a critical role. For example, PtRu‐alloy‐modified carbon nanofiber self‐supported electrodes can effectively suppress the accumulation of by‐products such as Li_2_CO_3_ during cycling, thereby preventing gradual interfacial deactivation [154]. Meanwhile, Pd and Ir, owing to their superior corrosion resistance, are able to maintain stable reaction interfaces in most electrolyte systems, enabling electrodes to sustain low polarization over extended cycling.

Conventional supported noble‐metal catalysts often suffer from particle detachment and interfacial disconnection, particularly under the vigorous gas‐liquid‐solid three‐phase reactions in LOBs. To overcome this limitation, noble‐metal‐carbon deep composite strategies proposed in recent years have attracted considerable attention. For instance, 3D Ru‐C composite structures derived from metal–organic frameworks (MOFs) enable uniform distribution of Ru nanoparticles within carbon scaffolds, fundamentally preventing particle detachment and achieving ultralong cycling life with high catalytic stability (Figure 6e) [155]. Such deep composite architectures provide an effective pathway for enhancing the durability of noble‐metal catalysts.

Noble‐metal catalysts including Pt, Pd, Au, Ru, and Ir can substantially enhance reaction kinetics and energy efficiency in LOBs and represent one of the most high‐performing classes of cathode catalytic materials to date. Nevertheless, their high cost, limited resource availability, and the electrochemical instability of certain materials at high potentials still restrict large‐scale practical applications [156]. Consequently, future research is increasingly directed toward bimetallic alloy design, such as PtRu and PdAu, single‐atom dispersion strategies, and multiscale noble‐metal‐carbon composite architectures to maximize catalytic utilization while reducing noble‐metal loading and further improving structural stability.

### 3.3 TMO‐Based Catalysts

TMOs, benefiting from tunable metal‐oxygen (M‐O) bond electronic structures, rich combinations of multivalent states, and excellent chemical stability, have emerged as one of the most promising cathode catalyst systems for LOBs [157]. Compared with conventional carbon materials, TMOs exhibit superior interfacial reaction tolerance, surface charge regulation capability, and lattice electronic structure flexibility, enabling higher reaction reversibility and cycling stability in bifunctional ORR/OER catalysis. In addition, strategies such as A/B‐site doping, oxygen‐vacancy engineering, crystal‐phase modulation, and hierarchical structural design allow coupled regulation of catalytic activity and interfacial stability over a broad parameter space, providing abundant material and structural design opportunities for developing high‐efficiency and durable LOBs. TMOs, featuring tunable metal‐oxygen electronic structures, multiple accessible valence states, and excellent chemical stability, have emerged as one of the most promising classes of cathode catalysts for LOBs [157]. Compared with conventional carbon‐based materials, TMOs exhibit superior tolerance to interfacial reactions, enhanced capability for surface charge modulation, and greater flexibility in electronic structure design, enabling improved reversibility and cycling stability in ORR/OER processes.

Despite the significant structural diversity among different types of TMOs, their catalytic behavior can be fundamentally attributed to the synergistic regulation of oxygen intermediate adsorption (O_2_^−^, LiO_2_, Li_2_O_2_), electron transfer pathways, and the kinetics of Li_2_O_2_ formation and decomposition. Therefore, a systematic understanding from a unified perspective encompassing structure type, electronic structure, and reaction mechanism is essential for establishing general design principles for advanced catalysts.

#### 3.3.1 Structural Types and Electronic Structure Regulation Mechanisms

(1) Layered Oxides: Layered manganese oxides, including δ‐MnO_2_, α‐MnO_2_, and β‐MnO2, have become among the most active oxide systems in LOBs [158], owing to their tunable Mn^3+^/Mn^4+^ oxidation states, abundant oxygen vacancies, and expandable interlayer channel structures. Their lamellar architecture effectively promotes O_2_ adsorption and activation, stabilizes LiO_2_ intermediates, and provides low‐energy‐barrier pathways for the nucleation and decomposition of Li_2_O_2_, endowing them with intrinsic advantages in enhancing ORR/OER reversibility.

**FIGURE 7:**
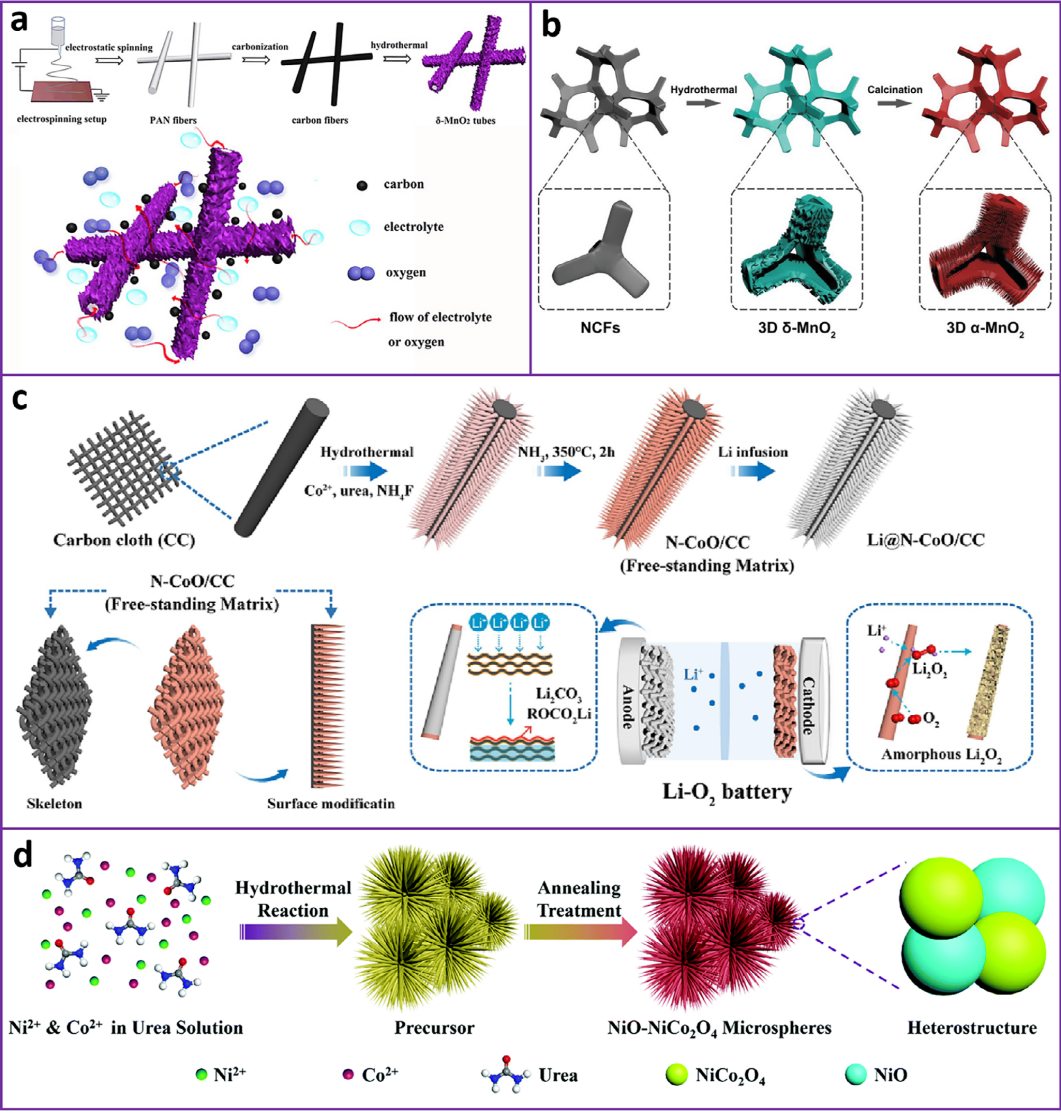
(a) Schematic illustration of the preparation of δ‐MnO_2_ tubes and the transfer of electrolyte and oxygen in the δ‐MnO_2_ tubes. Reproduced with permission [159]. Copyright 2015, Wiley. (b) Schematic illustration of the fabrication of 3D hollow α‐MnO_2_ framework. Reproduced with permission [154]. Copyright 2019, Wiley. (c) Schematic illustration and characterization of the N‐CoO/CC matrix. Reproduced with permission [165]. Copyright 2023, Elsevier. (d) Schematic illustration of the formation of NiO‐NiCo_2_O_4_ microspheres through a two‐step method. Reproduced with permission [166]. Copyright 2019, The Royal Society of Chemistry.

To improve the performance of layered MnO_2_, existing studies have pursued systematic regulation from structural, electronic, and interfacial perspectives. Porous δ‐MnO_2_ tubular architectures significantly enhance active‐site utilization, reduce OER overpotential, and induce the formation of thin‐sheet Li_2_O_2_, thereby delivering superior rate capability and cycling stability (Figure 7a) [159]. 3D hollow α‐MnO_2_ frameworks achieve more efficient oxygen diffusion and discharge‐product accommodation through large specific surface areas and interconnected pores, enabling electrodes to deliver discharge capacities exceeding 8500 mAh g^−1^ while maintaining long‐term cycling stability (Figure 7b) [160]. From the perspective of electronic structure regulation, modulation of Mn‐O bond characteristics using high‐valence clusters such as SeO_4_^2−^ stabilizes Jahn‐Teller‐activated structures and promotes reversible decomposition of Li_2_O_2_ (Figure 7c) [161]. Meanwhile, introducing Ru into the β‐MnO_2_ lattice further enhances lattice expansion and active‐site exposure and can even enable a four‐electron LiOH pathway under photo‐assisted conditions, representing an important direction for mechanistic innovation in MnO_2_‐based systems [162]. Constructing composite structures with carbon supports, such as α‐MnO_2_/NGNF and α‐MnO_2_/MWCNTs [163, 164], markedly improves electronic conductivity, enhances ORR/OER reversibility, and broadens the applicability of MnO_2_ across different battery systems (Figure 7d).

The hierarchical porous architecture, surface coordination regulation, and electronic structure reconstruction of layered MnO_2_ can synergistically reduce the energy barriers of oxygen reactions, enabling effective control over the morphology of Li_2_O_2_ and the associated reaction pathways. However, its intrinsically low electrical conductivity, along with structural instability and surface reconstruction under high‐voltage conditions, limits its long‐term cycling performance. Therefore, it is imperative to develop three‐dimensional crystalline systems with enhanced electronic structure tunability and improved conductivity.

The synergistic integration of hierarchical pore design, surface coordination regulation, and electronic structure reconstruction in layered MnO_2_ effectively lowers oxygen reaction energy barriers, directs discharge‐product evolution, and enables oxygen electrode reactions with high specific capacity and high reversibility. This structure‐electronic‐interfacial triple‐regulation framework provides a clear materials‐science pathway for constructing high‐performance cathode catalysts for LOBs.

(2) Spinel‐Structured Oxides: Compared with layered oxides that rely on two‐dimensional diffusion and surface adsorption regulation, spinel structures (such as Co_3_O_4_, MnCo_2_O_4_, NiCo_2_O_4_, and ZnCo_2_O_4_) exhibit more pronounced advantages in electronic structure modulation and electrical conductivity, owing to the synergistic interactions of A‐ and B‐site metal cations within their three‐dimensional lattice [167]. The catalytic performance of spinel oxides is primarily governed by regulation of the e_g_ orbital occupancy of B‐site metal centers, such as Co^2+^/Co^3+^ and Mn^3+^/Mn^4+^. When the e_g_ occupancy approaches an optimal range of 1.0‐1.2, the adsorption‐desorption balance of intermediates including O_2_^−^, LiO_2_, and Li_2_O_2_ is optimized, thereby significantly enhancing ORR/OER reversibility. In addition, the high density of surface steps and edge sites on spinel oxides strengthens O─O bond cleavage and LiO_2_ adsorption, while their relatively high electrical conductivity further suppresses interfacial corrosion under high charging potentials.

Through electronic structure modulation and the construction of 3D open architectures, the ability of spinel catalysts to regulate the nucleation, growth, and decomposition pathways of Li_2_O_2_ can be further enhanced. For example, self‐supported nitrogen‐doped CoO nanoarrays can induce the formation of amorphous Li_2_O_2_ and achieve ORR/OER at low polarization, while simultaneously suppressing lithium dendrite growth at the anode, thus exhibiting a synergistic optimization effect at both electrodes (Figure 7c) [165]. Multimetal spinel oxides, such as NiCo_2_O_4_ and ZnCo_2_O_4_, enable further optimization of e_g_ occupancy and oxygen vacancy concentration through heterometal incorporation with elements such as Zn, Ni, and Fe. Hierarchically porous NiCo_2_O_4_ foam architectures enhance electron and ion transport through interconnected pore networks and expose abundant active sites, delivering high specific capacities exceeding 10,000 mAh g^−1^ together with excellent energy efficiency [168]. Constructing NiCo_2_O_4_/BCN composite interfaces using high‐specific‐surface‐area BCN nanotubes as supports significantly strengthens electronic coupling, enabling low overpotential (≈0.96 V) and stable cycling over more than 300 cycles [169]. In addition, urchin‐like NiO‐NiCo_2_O_4_ dual‐oxide structures further improve oxygen reaction kinetics through synergistic interfacial effects, allowing batteries to maintain considerable capacity even under high‐rate conditions (Figure 7d) [166].

By virtue of tunable e_g_ electronic occupancy, heterometal doping, and hierarchical structural design, spinel oxides can establish truly reversible reaction pathways during Li_2_O_2_ formation and decomposition, making them one of the key catalytic platforms for achieving high‐energy‐efficiency LOBs. However, despite their advantages in electrical conductivity and bifunctional catalytic activity, spinel oxides still suffer from limitations in active site distribution and interfacial stability, particularly in complex electrolyte environments where side reactions are prone to occur. Therefore, developing catalytic systems with greater structural tunability and dynamic adaptability has become an important direction for future research.

(3) Perovskite‐Structured Oxides: Building upon these advances, perovskite oxides (ABO_3_) offer a higher degree of freedom in electronic structure design through dual‐site regulation at the A and B positions and the introduction of high concentrations of oxygen vacancies, enabling precise control over reaction pathways at the atomic scale [170]. Substitution at the A site (e.g., La, Sr, Ba) can modify lattice distortion and O 2p‐B 3d hybridization strength, thereby significantly influencing the adsorption behavior of oxygen intermediates. Regulation of B‐site TMs (e.g., Co, Fe, Mn, Ni) directly determines e_g_ occupancy and bifunctional catalytic performance. Extensive studies have demonstrated that when e_g_ ≈ 1, the adsorption strength of ORR/OER intermediates reaches an optimal balance, maximizing reaction reversibility. Moreover, reversible surface reconstruction of perovskites, such as changes in B‐site oxidation states and enrichment of surface oxygen vacancies, enhances interfacial adaptability via dynamic evolution mechanisms, effectively suppressing excessive Li_2_O_2_ growth and electrode passivation. Construction of double perovskite structures (A_2_BB'O_6_) and oxygen‐vacancy‐rich nonstoichiometric phases can enhance interfacial charge transport and reduce reaction energy barriers, establishing perovskites as important catalytic platforms for performance breakthroughs through precise electronic‐structure design.

Representative systems such as LaNiO_3_ and its Fe‐substituted derivatives LaNi_1‐x_Fe_x_O_3_ have demonstrated excellent bifunctional activity and structural stability [171, 172], serving as important examples of perovskite application in LOBs electrodes. By tuning e_g_ occupancy and the Mn^3+^/Mn^4+^ ratio through B‐site Ni doping in manganite perovskites La_0.7_Sr_0.3_Mn_1‐x_Ni_x_O (x = 0‐0.5), interfacial charge transfer is significantly accelerated, and the LiO_2_ to Li_2_O_2_ reaction pathway is regulated, enabling higher specific capacities with good cycling stability [173].

To further enhance active‐site exposure and gas diffusion efficiency, the design of hierarchical porous architectures has become an important strategy. Perovskite nanotube structures represented by La_0.5_Sr_0.5_CoO_3‐x_ (LSC) provide interconnected channels and high surface area, markedly improving Li_2_O_2_ storage capacity and reaction reversibility to achieve high round‐trip efficiency and prolonged cycle life [174]. Embedding Ba_0.5_Sr_0.5_Co_0.8_Fe_0.2_O_3‐δ_ (BSCF) into bamboo‐like carbon nanofiber architectures synergistically promotes O_2_ adsorption, electron transport, and Li_2_O_2_ decomposition via high oxygen‐vacancy concentrations and 3D conductive networks, delivering discharge capacities exceeding 11 000 mAh g^−1^ [175]. Constructing porous La_0.6_Ca_0.4_Fe_0.8_Ni_0.2_O_3_ (LCFN) nanotubes via cooperative A/B‐site doping further improves uniform Li_2_O_2_ deposition and reversible decomposition, achieving capacities above 13,000 mAh g^−1^ with stable cycling over more than one hundred cycles [176].

The high performance of perovskite oxides arises from the synergistic effects of A/B‐site electronic regulation, oxygen‐vacancy engineering, surface reconstruction, and hierarchical structural design. By integrating electronic‐structure engineering with structural engineering, perovskites enable performance leaps and represent a key development direction for future high‐efficiency LOB catalysts.

Layered, spinel, and perovskite oxides represent distinct stages of development, ranging from two‐dimensional diffusion regulation to three‐dimensional electronic structure modulation and ultimately to atomic‐scale electronic state enginee ring. Table 1 summarizes the key performance metrics of representative TMO‐based catalysts, including discharge capacity, overpotential, current density, and cycling performance, providing a direct basis for evaluating the advantages and limitations of different catalytic systems. The fundamental differences among these systems in regulating ORR/OER processes mainly lie in (i) the adsorption modes of oxygen intermediates, (ii) electron and ion transport pathways, and (iii) the mechanisms of Li_2_O_2_ formation and decomposition. However, current studies still lack a systematic and quantitative understanding of the correlation between structural features and reaction pathways, which, to some extent, limits the rational design of catalysts.

**TABLE 1:** Applications for Representative TMO‐based catalysts in LOBs.

Material	First discharge capacity (mAh g^−1^@mA g^−1^)	Overpotential (V)	Current rate (mA g^−1^)	Cycling performance	Refs.
δ‐MnO_2_	1425@600	—	100	50 cycles	[159]
3D α‐MnO_2_	8583@100	—	200	170 cycles	[155]
α‐MnO_2_ NWs	10000@200	—	200	70 cycles	[161]
α‐MnO_2_/MWCNTs	320@100	1.24	1000	133 cycles	[163]
N‐CoO/CC	2.33 mAh cm^−2@0.05^ mA cm^−2^	—	0.1 mA cm^−2^	200 cycles	[165]
3D NiCo_2_O_4_	10137@200	1.17	200	80 cycles	[168]
NCO@BCNNT	9823@100	0.97	500	320 cycles	[169]
NiO‐NiCo_2_O_4_	9231@100	1.48	100	80 cycles	[166]
La_0.5_Sr_0.5_CoO_3‐x_ nanotubes	5799@0.025 mA cm^−2^	1.14	0.1 mA cm^−2^	50 cycles	[168]
Ba_0.5_Sr_0.5_Co_0.8_Fe_0.2_O_3‐δ_(BSCF@C)	12708@200	0.74	500	98 cycles	[169]
La_0.6_Ca_0.4_Fe_0.8_Ni_0.2_O_3_ nanotubes	13019@400	—	400	64 cycles	[170]

#### 3.3.2 Key Regulation Strategies

The catalytic behavior of TMOs in LOBs strongly depends on their electronic structure, surface chemistry, and lattice defect characteristics [177]. Although different types of TMOs exhibit distinct advantages, their practical performance is still constrained by common issues, including insufficient electrical conductivity, mismatched intermediate adsorption, and interfacial side reactions. As a result, current research has gradually shifted from simple structural classification toward the development of regulation strategies that are guided by critical performance‐limiting factors.

(1) Oxygen‐Vacancy Engineering (enhancing O_2_ activation and conductivity): Oxygen vacancies can significantly enhance the intrinsic electrical conductivity of TMOs, strengthen electron donation from metal centers, and promote O_2_ adsorption and bond cleavage [178]. Introduction of high concentrations of oxygen vacancies via high‐temperature annealing, plasma treatment, or chemical reduction can effectively improve the adsorption and conversion of LiO_2_ intermediates, regulating Li_2_O_2_ nucleation density and deposition morphology while avoiding dense insulating layers and enhancing interfacial openness and reversibility [179].

In Mo‐based oxide systems, oxygen‐vacancy engineering has likewise been proven to be an effective approach for simultaneously improving conductivity and bifunctional activity. For instance, oxygen‐vacancy‐rich hexagonal h‐MoO_3_ (MoO_3_‐HTP) exhibits a much lower initial overpotential of approximately 1.58 V and achieves stable cycling for about 137 cycles at 200 mA g^−1^ with a limited capacity of 500 mAh g^−1^ [180]. Oxygen‐vacancy engineering, as demonstrated by density functional theory (DFT) and experimental results, enhances electronic conductivity, optimizes LiO_2_ and Li_2_O_2_ behaviors, reduces reaction barriers, and accelerates kinetics, offering an effective strategy for improving the catalytic performance of TMOs like MoO_3_ in LOBs.

(2) Heterometal Doping (optimizing electronic structure and adsorption energy): Heterometal doping, such as Zn, Ni, Fe, or Cu incorporation in spinel oxides, modifies M‐O bond covalency and tunes the d‐band center and e_g_ occupancy, thereby simultaneously optimizing ORR and OER activity [181, 182, 183]. For example, Zn doping increases the exposure of Co^3+^ species in spinel oxides, thereby significantly enhancing the initial activation of ORR, whereas Ni and Fe doping improve OER reversibility by strengthening metal‐oxygen charge transfer.

Synergistic co‐doping of TMOs is an effective strategy for enhancing catalytic performance. For instance, vertically aligned 3D porous Ni/Mn‐Co_3_O_4_ nanosheet arrays grown in situ on Ti substrates significantly increase active‐site density for oxygen electrochemical reactions [184]. DFT analysis reveals that Ni and Mn co‐doping enhances Co‐O bond covalency and induces charge redistribution, optimizing intermediate adsorption energies to promote reversible Li_2_O_2_ formation and decomposition while suppressing side reactions. A remarkably low total overpotential of approximately 0.61 V and stable cycling for about 850 cycles demonstrate highly efficient bifunctional ORR/OER catalysis. These results highlight the critical role of heterometal co‐doping in regulating electronic structure, intermediate adsorption behavior, and interfacial charge‐transfer kinetics of TMOs.

(3) Facet Engineering (regulating reaction pathways): Facet engineering, which tailors exposed crystal planes to modify surface atomic coordination and reaction pathways, represents another powerful approach to enhancing TMO catalytic performance [185]. In manganese oxides, distinct crystal facets exhibit pronounced differences in Li_2_O_2_ adsorption energy and decomposition kinetics, enabling fine regulation of discharge‐product morphology and reaction barriers through solvothermal synthesis and oriented crystal growth.

Ultrathin Mn_3_O_4_ nanosheet/graphene composites (Mn_3_O_4_ NS/G) with preferentially exposed (1 0 1) facets and high oxygen‐vacancy density show superior electrocatalytic performance compared with conventional Mn_3_O_4_ nanoparticles [186]. The Mn_3_O_4_ NS/G electrode delivers a charging overpotential of only ∼0.86 V, a reversible capacity up to 35,583 mAh g^−1^ at 200 mA g^−1^, and ultralong stable operation exceeding 1300 h. DFT calculations indicate that the (1 0 1) facet exhibits lower Li_2_O_2_ adsorption energy than the (2 1 1) facet, facilitating reversible decomposition and significantly reducing OER barriers.

In Co_3_O_4_ systems, different crystal facets likewise exhibit pronounced differences in the decomposition behavior of Li_2_O_2_. A representative study constructed a Li_2_O_2_/ Co_3_O_4_ interfacial model to investigate facet‐dependent reaction mechanisms [187]. The results reveal that the oxygen‐rich Co_3_O_4_ (1 1 1) facet possesses lower surface energy under high oxygen partial pressure and can significantly reduce charging overpotential and the O_2_ desorption barrier through electron transfer from the Li_2_O_2_ layer to the Co_3_O_4_ substrate, thereby exhibiting superior OER catalytic activity. In addition, further TM‐doping simulations indicate that p‐type doping of the Co_3_O_4_ (1 1 1) facet, such as with TM elements of higher ionization potential, can markedly facilitate charge extraction and O_2_ desorption, leading to a further reduction in OER overpotential.

These findings demonstrate that the combination of facet engineering and doping regulation enables simultaneous optimization of surface electronic structure, reaction‐intermediate adsorption behavior, and ORR/OER kinetics within a single crystal framework, providing an actionable crystallochemical basis for the efficient design of oxide catalysts such as Co_3_O_4_ and Mn_3_O_4_.

(4) Construction of Heterointerfaces or Core‐Shell Structures (enhancing interfacial synergy): Constructing heterointerfaces or core‐shell structures between TMOs and carbon materials, metal nanoparticles, or other oxides induces interfacial charge redistribution and band bending [188, 189, 190], significantly reducing reaction barriers and amplifying synergistic electrochemistry. Nitrogen‐doped carbon, hollow carbon spheres, and porous carbon frameworks are commonly employed as support to enhance electrical conductivity and specific surface area [191, 192], and when coupled with TMOs, they enable complementary advantages of a highly conductive scaffold and highly active interfaces.

For example, uniformly anchoring CoO‐Co_3_O_4_ nanoparticles onto nitrogen‐doped hollow carbon spheres (N‐HC@CoO‐Co_3_O_4_) yields a composite structure featuring high surface area, high conductivity, and abundant interfacial active sites [193]. The nitrogen‐doped carbon framework provides rapid electron‐transport pathways while forming strongly coupled interfaces with CoO/Co_3_O_4_, thereby facilitating the adsorption and conversion of oxygen species. This structure delivers an ultrahigh discharge capacity of up to 24,265 mAh g^−1^ at 300 mA g^1^ and sustains stable cycling for 112 cycles under a limited capacity of 500 mAh g^−1^.

In multiphase composite oxide catalytic systems, interfacial coupling between TMOs and rare‐earth oxides likewise exhibits pronounced performance enhancement. NiCo_2_O_4_@CeO_2_ composite microspheres prepared via hydrothermal synthesis followed by annealing consist of hierarchically porous nanoneedle bundles integrated within an overall spherical framework [194]. This architecture forms a highly open 3D porous network that facilitates rapid diffusion of O_2_ and Li^+^ as well as thorough electrolyte infiltration. More importantly, the incorporation of CeO_2_ not only induces abundant oxygen vacancies on the NiCo_2_O_4_ surface but also optimizes the electronic structure of Ni/Co metal centers through Ce‐O‐Co interfacial electronic reconstruction. This synergistic effect markedly enhances the overall electronic transport capability and bifunctional catalytic activity of the electrode. As a result, the total overpotential of the corresponding LOB is effectively reduced to 1.07 V. Stable cycling over 400 cycles is achieved at a current density of 500 mA g^−1^, with performance significantly superior to that of pristine NiCo_2_O_4_.

Although TMOs have demonstrated significant advantages in facilitating reversible Li_2_O_2_ conversion, their further development still faces several critical challenges. First, there is a lack of quantitative correlation between electronic structure descriptors and actual reaction pathways, leading to catalyst design that remains largely empirical. Second, the mechanisms of surface reconstruction and phase transformation under high‐voltage conditions are not yet fully understood. Third, the interactions between parasitic products and catalytic active sites are highly complex. Fourth, a unified theoretical framework describing multiscale structural synergistic effects is still lacking.

Therefore, future research should move beyond single‐structure optimization toward a new paradigm based on the synergistic regulation of electronic structure, interfacial processes, and reaction pathways. Establishing quantitative structure–performance relationships through the integration of in situ or operando characterization and first‐principles calculations will be essential. In addition, the incorporation of high‐throughput computation and artificial intelligence approaches is expected to enable a transition from empirical screening to predictive design, thereby accelerating the development and practical implementation of high‐performance TMO catalysts.

### 3.4 TM‐Sulfide‐, Nitride‐, and Carbide‐Based Catalysts

TM sulfides, nitrides, and carbides exhibit metallic or quasi‐metallic electrical conductivity, tunable coordination environments, and engineerable d‐orbital electronic structures, while maintaining relatively high chemical stability [195, 196, 197]. These materials not only significantly reduce the reaction energy barriers of ORR/OER but also enable precise regulation of Li_2_O_2_ nucleation, growth, and deposition morphology during discharge, as well as promote its decomposition during charging, thereby synergistically enhancing specific capacity, energy efficiency, and cycle life [198]. Through strategies such as crystal‐phase regulation, defect engineering, heteroatom doping, and compositing with conductive carbon materials, interfacial electronic coupling and intermediate adsorption can be further amplified.

#### 3.4.1 Chalcogenide‐Based Catalysts

TM sulfides, including CoS_2_, MoS_2_, and Ni_3_S_2_, possess multivalent metal‐sulfur bonds and semi‐metallic or metallic electronic structures, exhibiting outstanding capability in promoting the reversible formation and decomposition of Li_2_O_2_ [199, 200]. Their layered or cubic lattices provide tunable active sites for LiO_2_ stabilization and suppress electrode passivation, while simultaneously offering relatively open pathways for charge and gas transport. When integrated with carbon materials to form continuous conductive networks, their rate capability and cycling stability can be further enhanced, highlighting the structure‐electronic synergy of chalcogenides in bifunctional catalytic systems.

**FIGURE 8:**
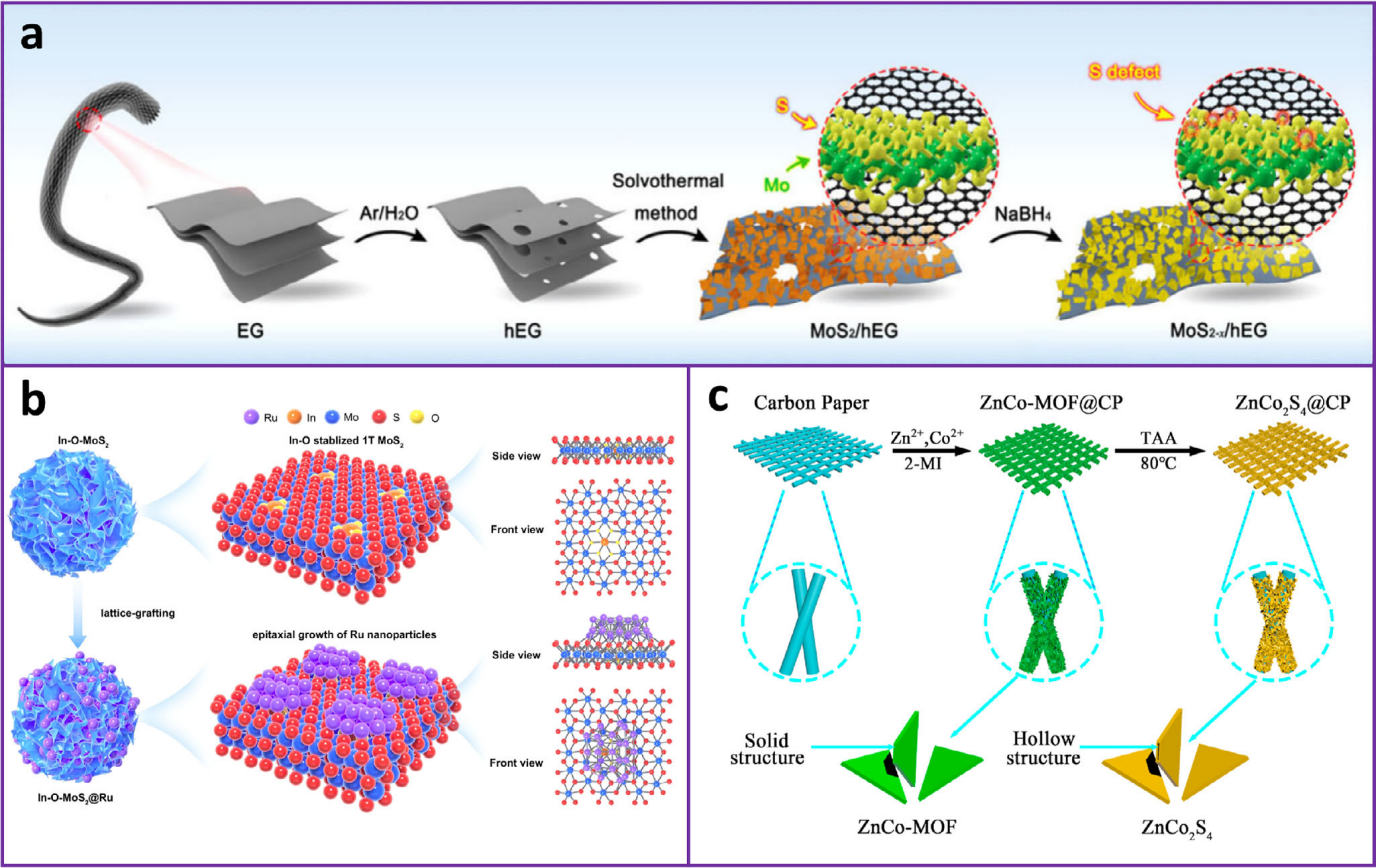
(a) Scheme of the synthetic procedures of MoS_2‐x_/hEG. Reproduced with permission [201]. Copyright 2024, American Chemical Society. (b) A Schematic illustration for the synthesis of In‐O‐MoS_2_@Ru. The Mo, S, Ru, In, and O atoms are described by blue, red, purple, orange, and yellow balls, respectively. Reproduced with permission [44]. Copyright 2025, Springer Nature. (c) Fabrication process of ZnCo_2_S_4_ nanosheet array on carbon paper. Reproduced with permission [202]. Copyright 2021, Elsevier.

In MoS_2_‐based systems, the synergistic construction of defect engineering and conductive frameworks has been demonstrated as an effective strategy to overcome kinetic limitations [201]. For instance, heterostructures composed of sulfur‐vacancy‐rich MoS_2‐x_ nanosheets and highly conductive holey expanded graphite (MoS_2‐x_/hEG) introduce abundant accessible active sites, while establishing efficient electron‐transport pathways through the continuous carbon scaffold (Figure 8a) [201]. Sulfur vacancies can regulate the electronic distribution of metal centers, enhance chemical adsorption of LiO_2_ intermediates, and lower the nucleation energy barrier of Li_2_O_2_. Meanwhile, the interconnected pores of hEG improve O_2_ diffusion and electrolyte wettability, preventing dense Li_2_O_2_ accumulation and electrode passivation, thereby enabling high discharge capacity and prolonged cycle life.

Beyond defect regulation, fine‐tuning of phase structure and surface orbital configuration can further strengthen the bifunctional catalytic performance of MoS_2_. Stabilizing the metallic 1T phase of MoS_2_ via p‐block element In‐O regulation and epitaxially anchoring Ru nanoscopic sites on its surface overcomes orbital steric hindrance and internal charge‐coupling limitations present in conventional 1T‐MoS_2_ (Figure 8b) [44]. Such In‐O‐MoS_2_@Ru architectures enhance O_2_ dissociation while simultaneously improving adsorption kinetics of LiO_2_ intermediates, directing the formation of weakly crystalline, thin‐film Li_2_O_2_ that is more readily decomposed during charging. Consequently, ORR/OER overpotentials are significantly reduced, and cycle life is markedly extended.

In multimetal sulfide systems, compositional regulation of metal active‐center valence states and exposure constitutes another key strategy for enhancing bifunctional catalytic performance. For example, incorporation of Zn into ZnCo_2_S_4_ induces a higher population of surface‐exposed Co^3+^ active sites, thereby substantially improving ORR/OER activity. Complementary hollow nanosheet arrays and 3D porous architectures shorten O_2_ and Li^+^ transport pathways and buffer volume variation during cycling, preventing structural collapse and electrode deactivation. As a result, stable cycling at high capacity and low overpotential can be achieved (Figure 8c) [202].

Beyond single sulfide systems, coupling sulfide/selenide phases such as Co/CoSe with N, Se co‐doped carbon frameworks and constructing 3D self‐supported architectures further amplifies electrocatalytic synergy [203]. This design leverages interfacial electronic reconstruction at Co/CoSe junctions, defect sites introduced by N/Se heteroatoms, and rapid gas/ion transport enabled by 3D porous networks. Consequently, electrodes can deliver high areal capacity at relatively low current densities while maintaining stable cycling over multiple cycles, demonstrating the competitive advantages of sulfide/selenide heterostructures as LOB cathodes.

TM chalcogenides, benefiting from tunable metal‐sulfur electronic structures, engineerable layered or hollow hierarchical architectures, and strong interfacial coupling with conductive carbon scaffolds, exhibit exceptional capability in promoting reversible Li_2_O_2_ formation and decomposition. Through defect engineering, phase regulation, heterometal incorporation, and heterointerface construction, chalcogenides have evolved into efficient catalytic systems integrating electronic‐structure engineering, defect control, and multiphase synergy. These attributes provide an important materials foundation for developing LOBs cathodes with high energy efficiency and high reversibility.

#### 3.4.2 Nitride‐Based Catalysts

Compared with oxides and sulfides, TM nitrides possess electronic structures closer to those of metals and higher intrinsic electrical conductivity [204]. Strong metal‐nitrogen coordination not only stabilizes LiO_2_ intermediates and lowers OER energy barriers but also provides additional active sites through surface nitrogen vacancies, thereby delivering superior bifunctional ORR/OER catalytic performance. Recent studies indicate that introducing auxiliary metals to regulate the d‐orbital electron distribution and e_g_ occupancy of metal centers is an effective approach to further enhance nitride catalytic activity.

**FIGURE 9:**
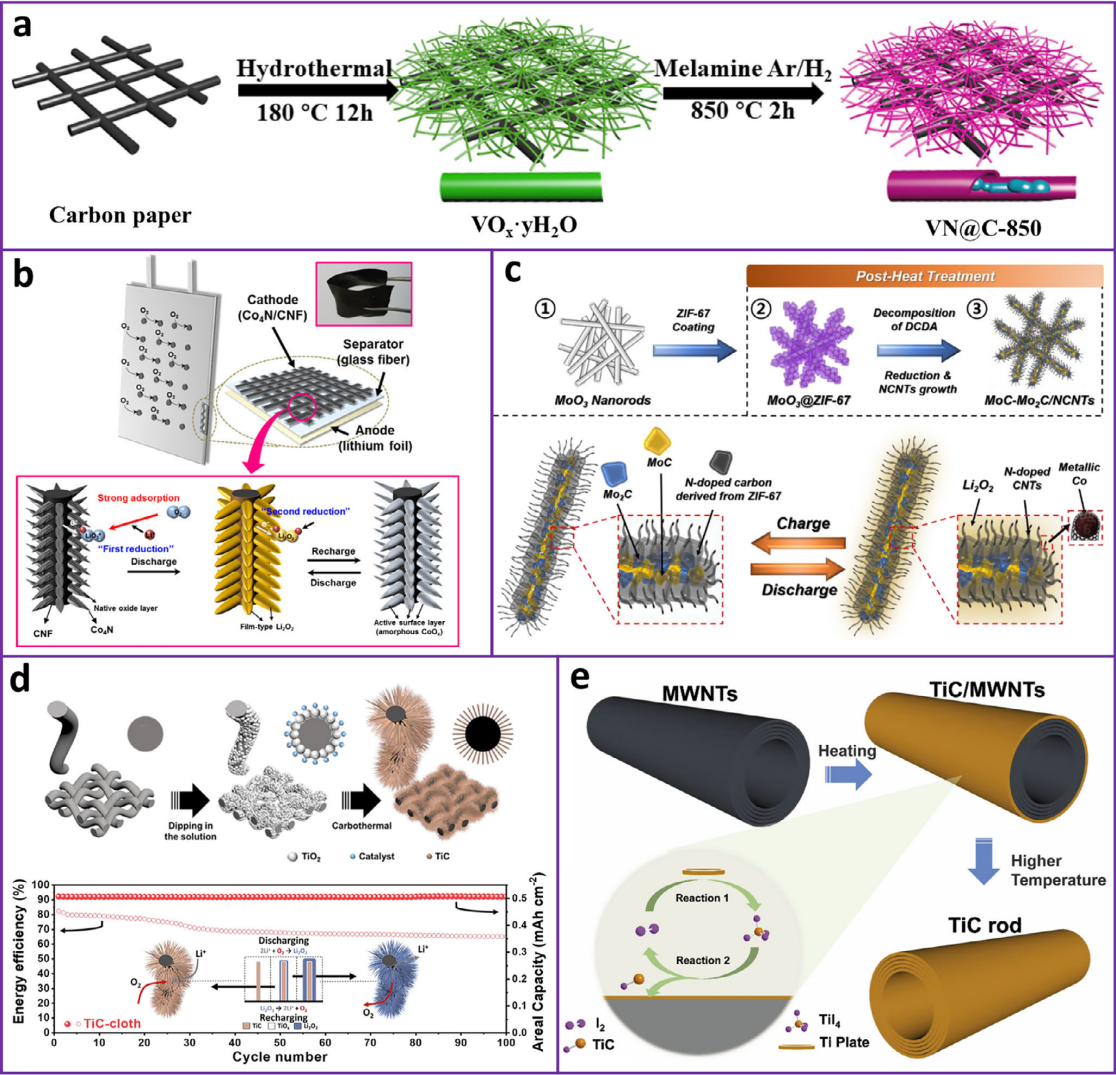
(a) Schematic illustration of the VN@C‐850 fabrication process. Reproduced with permission [205]. Copyright 2020, Elsevier. (b) Proposed reaction mechanism of LOBs with Co_4_N/CNF electrode. Reproduced with permission [206]. Copyright 2018, American Chemical Society. (c) Schematic illustration of the preparation of house centipede‐like MoC‐Mo_2_C/NCNT nanorods. Reproduced with permission [208]. Copyright 2020, Elsevier. (d) Schematic representation of TiC‐cloth fabrication from a piece of cotton T‐shirt. Reproduced with permission [211]. Copyright 2020, Elsevier. (e) Schematic illustration of synthesis of TiC/MWNTs. Reproduced with permission [212]. Copyright 2020, Elsevier.

Among various nitrides, vanadium nitride (VN) is considered a highly promising bifunctional catalyst owing to its high electronic conductivity, excellent chemical stability, and metallic‐like electronic structure. By in situ growth of VN followed by encapsulation with nitrogen‐doped carbon to construct VN@C composite cathodes, binder‐free self‐supported electrodes can be fabricated on carbon paper (Figure 9a) [205]. The nitrogen‐doped carbon layer markedly improves the structural stability and corrosion resistance of VN particles while simultaneously enhancing ORR/OER catalytic activity. At the interface, the carbon coating optimizes the adsorption and release behavior of LiO_2_/Li_2_O_2_ intermediates, ultimately achieving high initial specific capacity, a small charge‐discharge voltage gap, and a cycle life far superior to that of uncoated VN.

Co_4_N /CNF integrated air electrodes based on cobalt nitride are fabricated by growing Co(OH)F on CNF films followed by nitridation to form brush‐like Co_4_N nanorod arrays uniformly anchored on conductive CNF networks (Figure 9b) [206]. This architecture constructs continuous electron‐transport pathways and abundant active sites, enabling efficient electrochemical reactions. During cycling, a thin oxide layer formed on the Co_4_N surface facilitates reversible formation and decomposition of thin‐film Li_2_O_2_, thereby significantly reducing overpotential. The electrode maintains stable output performance under repeated cycling and mechanical bending, demonstrating the adaptability of nitride catalysts under flexible and practical operating conditions.

Benefiting from metallic‐like electronic structures, high conductivity, and strong metal‐nitrogen coordination, TM nitrides exhibit pronounced advantages in strengthening LiO_2_ intermediate adsorption, lowering OER energy barriers, and enhancing bifunctional catalytic activity. Through nitrogen‐doped carbon encapsulation, interfacial coupling, and defect or vacancy regulation, electron transport can be further improved, and side reactions effectively suppressed. Meanwhile, fine regulation of d‐orbital electron distribution and e_g_ occupancy enables nitrides to evolve from conventional conductivity‐enhancing phases into core catalytic systems capable of achieving highly reversible oxygen electrochemistry via coordination and interfacial engineering, forming a complementary catalytic gradient with chalcogenide‐based catalysts.

#### 3.4.3 Carbide‐Based Catalysts

TM carbides are regarded as “noble‐metal‐like catalysts” due to their noble‐metal‐like d‐band electronic structures, high electrical conductivity, and excellent chemical stability [207]. Among them, molybdenum carbides (MoC, Mo_2_C), possessing electronic structures similar to those of Pt‐group metals, are considered ideal candidates for achieving efficient ORR/OER in LOB systems. Titanium carbide (TiC) and tungsten carbide (WC), benefiting from high hardness, corrosion resistance, and electrical conductivity, mainly serve to enhance electrode stability and suppress carbon‐support degradation.

In molybdenum‐carbide‐based catalysts, MoC‐Mo_2_C/NCNTs composites derived from ZIF‐67‐coated MoO_3_ nanorods form a hierarchical centipede‐like architecture. In this structure, MoC‐Mo_2_C nanocrystals act as the main backbone, while radially distributed Co@NCNTs resemble legs (Figure 9c) [208]. This architecture significantly increases specific surface area and active‐site density, while establishing continuous electron‐transport pathways for efficient Li_2_O_2_ formation and decomposition. As a result, ultralow overpotential, high specific capacity, and prolonged cycle life are achieved, demonstrating the synergistic bifunctional catalysis of molybdenum carbide and nitrogen‐doped carbon nanotube heterostructures.

Ti‐based carbides are representative materials for improving electrode stability. TiC, with excellent electrical conductivity and corrosion resistance, is widely used to suppress carbon‐cathode degradation and enhance electrochemical reversibility [209, 210]. For example, TiC‐coated carbon cloth prepared via carbothermal reduction uniformly covers carbon fibers with TiC nanostructures, protecting the carbon support from high‐voltage decomposition while providing porous space for Li_2_O_2_ accommodation. Li_2_O_2_ growing along TiC orientations can undergo reversible formation and decomposition, thereby improving cycling stability without sacrificing electrical conductivity (Figure 9d) [211]. Furthermore, in situ construction of ultrathin TiC shells (∼3 nm) on multiwalled carbon nanotube frameworks to form TiC/MWNT composite electrodes combines the high conductivity of CNTs with the resistance of TiC to O_2_^−^ corrosion. This design effectively suppresses Li_2_CO_3_ by‐product formation and achieves extended cycle life after Ru catalyst loading (Figure 9e) [212].

Tungsten carbide (WC) likewise exhibits high electrical conductivity and chemical stability, effectively promoting electron transfer during ORR. Previous first‐principles studies have systematically analyzed the catalytic mechanisms of W_n+1_C_n_ and oxygen‐functionalized W_n+1_C_n_O_2_ (n = 1, 2, 3) MXenes in LOBs cathode reactions [213]. These MXenes show excellent metallicity and increasing conductivity with layer number, while surface oxidation converts electrophilic W surfaces into electronegative O‐terminated surfaces, facilitating Li‐O bond activation and Li^+^ deintercalation. Correlations between reaction barriers and adsorption energies indicate that tuning band‐center positions, oxygen functionalization, and layer thickness weakens Li_x_O_2_* intermediate adsorption, thereby simultaneously reducing ORR/OER overpotentials. Among them, W_4_C_3_O_2_ MXene exhibits an ultralow total overpotential of 0.63 V and outstanding electronic conductivity, making it a highly promising LOBs cathode catalyst. These results suggest that crystal‐phase regulation, MXene structural evolution, single‐atom or multimetal synergy, and interfacial charge redistribution are emerging as key directions for advancing carbide catalyst performance.

Owing to their noble‐metal‐like d‐band structures, high conductivity, and chemical stability, TM carbides have become important materials for enhancing bifunctional ORR/OER catalysis in LOBs [214]. By constructing heterointerfaces with conductive carbon scaffolds and achieving atomic‐level dispersion and epitaxial structural synergy, electron transport can be enhanced, LiO_2_/Li_2_O adsorption‐conversion optimized, and carbon corrosion and by‐product accumulation effectively suppressed. As theoretical calculations continue to elucidate quantitative relationships among d‐band centers, adsorption energies, and reaction barriers, carbides are evolving from noble‐metal substitutes into designable electronic‐structure regulation platforms.

TM sulfides, nitrides, and carbides, owing to their tunable structures, metallic‐like electrical conductivity, and adjustable metal‐nonmetal coordination environments, have demonstrated significant potential in cathode catalysis for LOBs. Compared with conventional oxide systems, these materials exhibit advantages in electron transport and interfacial reaction kinetics, which contribute to reduced reaction polarization and promote the reversible formation and decomposition of Li_2_O_2_. To more intuitively compare the performance of different systems, Table 2 systematically summarizes and compares key metrics of representative TM sulfide, nitride, and carbide catalysts, including discharge capacity, overpotential, current density, and cycling stability. As shown in the table, distinct differences in performance exist among various material systems, indicating that compositional regulation and structural design enable differentiated optimization across multiple performance parameters.

**TABLE 2:** Applications for Representative TM‐based Sulfide, Nitride, and Carbide catalysts in LOBs.

Material	First Discharge Capacity (mAh g^−1^@mA g^−1^)	Overpotential (V)	Current Rate (mA g^−1^)	Cycling performance	Refs.
MoS_2‐x_/hEG	19000.3@500	1	1000	500	[201]
In‐O‐MoS_2_@Ru	19800@200	0.37	200	284	[44]
ZnCo_2_S_4_	9505@100	1.02	100	90	[202]
Co‐CoSe@NSeC/bioC	9.1 mAh cm^−2@0.05^ mA cm^−2^	0.73	0.05 mA cm^−2^	76	[203]
VN@C‐850	8269@100	0.88	100	183	[205]
Co_4_N/CNF	11.9 mAh cm^−2@0.05^ mA cm^−2^	1.23	500	177	[206]
MoC‐Mo_2_C/NCNTs	34862@200	0.5	200	162	[208]

These materials still face several challenges, including insufficient utilization of active sites, interfacial side reactions, and limited structural stability. Future efforts may focus on constructing multiphase composite architectures, atomic‐level regulation of metal‐nonmetal coordination environments, and self‐supported electrode systems to achieve synergistic enhancement of activity and stability. On this basis, integrating in situ characterization with theoretical calculations to tailor electronic and interfacial structures for different reaction pathways and operating conditions will further advance the practical application of high‐energy‐density LOBs.

### 3.5 RMs

In LOBs, the electrically insulating nature of the discharge product Li_2_O_2_ and its nonuniform deposition on electrode surfaces often lead to sluggish ORR/OER kinetics, severe polarization, and poor cycling stability. In recent years, RMs, acting as soluble catalysts, have been widely employed to improve reaction kinetics at the electrode‐electrolyte interface [126]. In LOBs, the insolubility and insulating properties of Li_2_O_2_ in organic electrolytes result in interfacial contact instability, pore blockage, and kinetically limited reactions during repeated charge‐discharge processes. Although conventional solid‐state catalyst systems can promote electrochemical reactions to some extent, their solid‐liquid‐gas three‐phase interfacial reactions remain constrained, leading to overall sluggish kinetics and poor rate capability. To overcome these limitations, the use of soluble catalysts, namely RMs, has been proposed to enhance reaction reversibility and kinetic efficiency [215].

RMs can homogeneously dissolve in the electrolyte and fully contact all surfaces of discharge products, enabling solution‐phase electron transfer to effectively lower energy barriers during discharge and charge processes. RMs suppress the direct deposition of Li_2_O_2_ on electrode surfaces, thereby alleviating electrode passivation phenomena [216]. According to their dominant role in different electrochemical stages, RMs can be classified into ORR‐type and OER‐type. A common feature of both types is that solution‐phase electron transfer partially or fully shifts reactions originally confined to solid‐liquid interfaces into the electrolyte phase, thereby altering the formation, decomposition location, and morphological evolution pathways of Li_2_O_2_. Essentially, RMs do not merely accelerate reactions, but instead reconstruct the spatial domain and reaction pathways, mitigating long‐standing issues such as carbon‐cathode passivation, polarization accumulation, and amplification of side reactions.

#### 3.5.1 ORR‐Type RMs

The core function of ORR‐type RMs is not simply to accelerate surface reaction rates but to reconstruct the discharge process at the level of reaction pathways [217]. Existing studies indicate that such mediators enhance discharge kinetics and capacity mainly through two synergistic mechanisms: i) First, the solution‐phase electron‐shuttling mechanism operates when ORR‐type RMs undergo reversible redox reactions at the cathode and subsequently transfer electrons to dissolved oxygen, generating solution‐phase superoxide intermediates (LiO_2_ or its solvated forms). This pathway reduces the dependence of ORR on surface active sites and mitigates localized current‐density accumulation and pore blockage, provided that solution‐phase electron transfer and mass diffusion are sufficiently fast. ii) Second, the intermediate‐complexation and induced‐precipitation mechanism involves specific RMs interacting with LiO_2_ to form RM‐Li or RM‐LiO_2_ complexes. Such complexation weakens LiO_2_ adsorption and disproportionation on carbon surfaces, promotes Li_2_O_2_ nucleation in the solution phase, and enables the growth of more loosely packed and reversible particle‐ or sheet‐like morphologies, thereby delaying passivation and increasing capacity.

**FIGURE 10:**
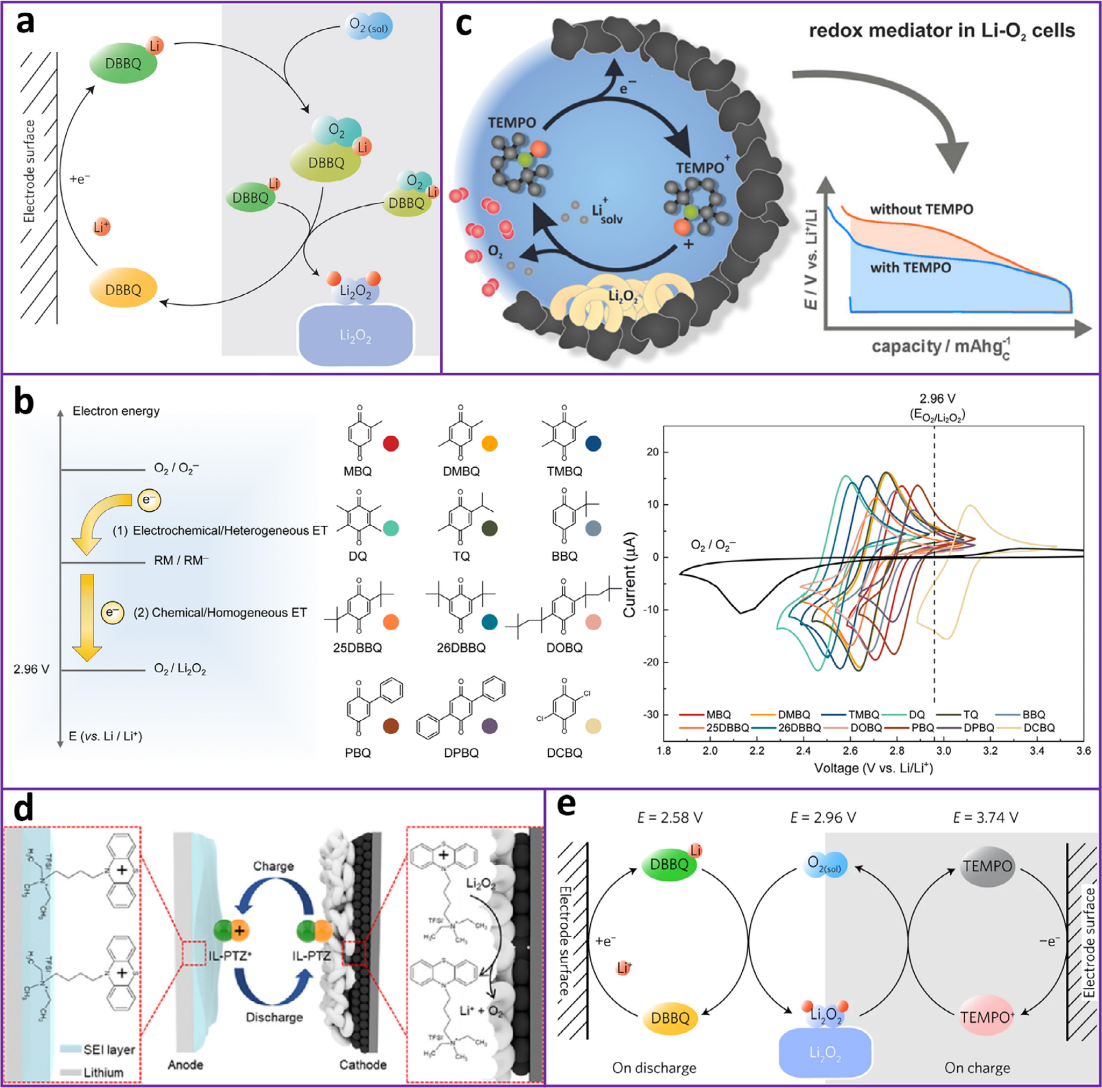
(a) How a shuttle transfers the oxygen reduction site away from the electrode surface. Reproduced with permission [218]. Copyright 2016, Springer Nature. (b) Energy relationship during ORR with a RM, various quinone derivatives, and their cyclic voltammetry curves at a scan rate of 50 mV s^−1^. Reproduced with permission [126]. Copyright 2023, Royal Society of Chemistry. (c) Proposed catalytic cycle for the electrochemical charging of LOBs with TEMPO. Reproduced with permission [226]. Copyright 2014, American Chemical Society. (d) A schematic diagram of ammonium ionic liquid functionalized phenothiazine as a new RM. Reproduced with permission [227]. Copyright 2022, American Chemical Society. (e) Schematics of positive electrode reactions on discharge and charge in the presence of DBBQ and TEMPO. Reproduced with permission [231]. Copyright 2017, Springer Nature.

A critical trade‐off underlying these mechanisms is that the redox potential of the RM is neither “the higher the better” nor “the lower the safer”. Early mediators, such as viologen derivatives (EtV(OTf)_2_) and certain quinones (2, 5‐di‐tert‐butyl‐1, 4‐benzoquinone, DBBQ) [218, 219], have demonstrated that solution‐phase pathways can substantially improve discharge behavior (Figure 10a). However, these studies also revealed a general dilemma in which overly low redox potentials provide insufficient driving force for solution‐phase ORR, leaving a considerable fraction of surface reduction and associated passivation. Conversely, excessively high redox potential can in principle suppress surface pathways and enforce solution‐mediated routes but may introduce stronger driving forces for side reactions with electrolytes, salts, or impurities, while not necessarily guaranteeing faster kinetics. Therefore, evaluation of ORR‐type RMs should extend beyond capacity enhancement alone to assess whether surface pathways are truly suppressed, whether Li_2_O_2_ forms away from the electrode surface, and whether such behavior can be sustained over multiple cycles.

Moving from material examples to design principles, molecular structure and steric effects are decisive factors governing the true reaction rate. Compounds such as trityl isothiocyanate [220], coenzyme Q_10_ [221], and polyoxometalates have been reported to exhibit effective ORR mediation [222], further improving discharge kinetics and energy efficiency. Systematic studies on benzoquinone derivatives suggest that reactions between RMs and O_2_ or LiO_2_ often proceed via inner‐sphere electron transfer or at least require effective molecular contact. Consequently, steric hindrance, molecular conformation, and conjugation directly influence apparent reaction rates (Figure 10b) [126]. Molecules with large steric bulk and loose spatial configurations may exhibit limited reactivity despite more positive redox potentials due to restricted access to O_2_ or LiO_2_ species. In contrast, compact and highly conjugated molecules facilitate electron delocalization and diffusion‐reaction coupling, resulting in superior performance under practical battery conditions. These observations imply that ORR‐type RM design should transition from a potential‐centered approach toward comprehensive optimization of redox potential, complexation capability, diffusion and steric characteristics, and solvation stability.

Regarding the “double‐edged sword” effect of water, its fundamental role lies in modifying solvation and nucleation free energies. Trace amounts of water enhance intermediate solvation, lower nucleation barriers, and promote solution‐mediated pathways, thereby markedly altering Li_2_O_2_ morphology and increasing capacity. However, excessive water introduces severe side reactions with metallic lithium and poses significant safety risks [223, 224]. Thus, the influence of water is not merely an increase in activity but a modulation of the thermodynamic and kinetic boundaries of LiO_2_/Li_2_O_2_ via microenvironment control. This insight further suggests more controllable strategies, such as employing designed hydrogen‐bond donors or acceptors, ionic liquids, or localized high‐concentration electrolytes to replicate the beneficial effects of water while avoiding its detrimental consequences.

#### 3.5.2 OER‐Type RMs

OER‐type RMs primarily function during the charging process, and their operating mechanism is relatively well defined. Specifically, the RM is electrochemically oxidized at the cathode to a high‐valence state (RM^+^), which subsequently chemically oxidizes Li_2_O_2_ in the solution phase into Li^+^ and O_2_. In this way, the high‐barrier charge‐transfer process originally occurring at the solid Li_2_O_2_/carbon interface is transformed into a solution‐phase chemical reaction, thereby significantly reducing charging overpotential and mitigating oxidative decomposition of carbon electrodes and electrolytes under high voltages [225].

OER‐type RMs encompass a wide range of materials, including organic molecules, organometallic salts, and inorganic compounds. In 2013, Bruce and co‐workers first introduced tetrathiafulvalene into LOB systems, achieving a notable improvement in cycling stability [73]. Subsequently, 2,2,6,6‐tetramethylpiperidine‐1‐oxyl (TEMPO), possessing excellent electrochemical stability and an appropriate redox potential, was shown to reduce the charging overpotential to 3.5–3.7 V (Figure 10c) [226]. However, the central challenge associated with OER‐type RMs is more acute, as stronger oxidative capability inevitably increases the likelihood of side reactions involving electrolytes, salt anions, impurities, and even separators or binders. Moreover, oxidized RMs are more prone to shuttle toward the anode and react with metallic lithium. Therefore, rational design of OER‐type RMs should advance from simple potential matching to considerations of reaction selectivity and spatial controllability. Such design must ensure preferential chemical oxidation of Li_2_O_2_ in the oxidized state while restricting undesired reactions with electrolytes and the lithium anode through molecular structure and interfacial engineering, and maintaining a stable, regenerable active state during repeated cycling.

Furthermore, the concept of ionic‐liquid‐functionalized mediators has been proposed, whose significance lies not in introducing merely another molecule but in highlighting a general strategy. By leveraging molecular volume, solvation shells, and ion‐pair structures, RM activity and side reactions can be spatially isolated, thereby improving compatibility without sacrificing kinetics. For example, ammonium ionic‐liquid‐functionalized phenothiazine effectively suppresses side reactions between the RM and metallic lithium through spatial protection provided by the ionic‐liquid structure [227], resulting in enhanced cycling stability (Figure 10d). In addition, halide‐based RMs containing I^−^ or Br^−^, such as LiI and LiBr, can participate in Li_2_O_2_ decomposition via changes in halide oxidation states, further catalyzing charge‐discharge reactions and reducing overpotential [228, 229].

#### 3.5.3 Multi‐Mediator Synergy: Reaction‐Architecture Decoupling

With a deeper understanding of solution‐phase reactions, a consensus has emerged that a single RM is rarely capable of simultaneously optimizing both discharge and charge processes. This limitation arises because the ideal properties required of RMs at the two stages are nearly opposite in nature. During discharge, RMs are expected to promote solution‐mediated pathways and induce reversible product morphologies [230]. In contrast, charging requires RMs with sufficient chemical oxidation capability while avoiding electrolyte and carbon corrosion.

Therefore, the significance of multi‐mediator synergy does not lie in an empirical “1 + 1 > 2” effect, but rather in enabling decoupling at the level of reaction architecture. In such systems, discharge is governed by ORR‐type RMs that direct solution‐phase formation of Li_2_O_2_, whereas charging is dominated by OER‐type RMs that drive its solution‐phase decomposition. As a result, electron transfer and Li_2_O_2_ nucleation/decomposition are no longer strongly coupled to the carbon surface. Bruce and co‐workers proposed a “dual‐mediator” strategy that, for the first time, achieves full solution‐phase decoupling of Li_2_O_2_ formation and decomposition in LOBs (Figure 10e) [231]. During discharge, benzoquinone‐based DBBQ, acting as an ORR‐type RM, drives solution‐phase reduction of O_2_ to Li_2_O_2_, preventing the formation of dense insulating films on carbon electrodes. During charging, TEMPO, serving as an OER‐type RM, efficiently decomposes solution‐phase Li_2_O_2_ via chemical oxidation, markedly suppressing high‐voltage polarization at conventional solid‐liquid interfaces. This dual‐mediator system enables low‐polarization and highly reversible electrochemical cycling. It reduces the carbon‐cathode decomposition rate from 0.12% to below 0.008%. A reversible areal capacity of up to 2 mAh cm^−2^ is achieved at 1 mA cm^−2^. Under higher‐rate conditions, the system even exhibits a theoretical capacity potential of up to 40 mAh cm^−2^.

The dual‐mediator strategy provides the first relatively complete demonstration of the feasibility of fully solution‐mediated Li_2_O_2_ generation and decomposition. It simultaneously advances the reduction of polarization and suppression of carbon degradation to a new, quantifiable level [232]. More importantly, this concept implicitly defines new evaluation criteria for the next research stage. Beyond capacity and overpotential, attention must be paid to whether Li_2_O_2_ truly forms away from the carbon surface and whether polarization accumulates during cycling. It is also essential to determine whether RMs are continuously consumed by side reactions. Rather than merely proving feasibility, systems must demonstrate sustained solution‐phase dominance under conditions closer to practical operation, such as high areal capacity, high rates, and limited electrolyte volumes.

RMs introduce solution‐phase electron‐transfer pathways and regulate Li_2_O_2_ nucleation, growth, and decomposition behaviors. In doing so, they provide effective means to alleviate kinetic sluggishness, polarization buildup, and limited reversibility that are pervasive in LOBs [233]. However, further progress toward practical application remains constrained by multiple factors: i) First, shuttle effects and incompatibility with lithium metal anodes are particularly severe, especially for OER‐type RMs in highly oxidized states; ii) Second, coupling decomposition among RMs, electrolytes, and carbon supports under high charging voltages can restrict the stable potential window; iii) Third, the effectiveness of RMs strongly depends on system‐specific microenvironments, including solvation structures, lithium salt chemistry, impurities, and water content. This dependence results in limited transferability and reproducibility across different studies. In addition, a systematic understanding of the intrinsic relationship between RM molecular structures and reaction‐pathway selectivity is still lacking. Many high‐performance systems therefore remain at an empirical level without predictive design rules.

Future research is expected to advance along several interrelated directions: i) First, molecular design principles centered on reaction‐pathway selectivity should be developed to quantitatively enhance solution‐phase contributions rather than merely pursuing higher redox potentials; ii) Second, controllable solvation and local microenvironment engineering should be employed to reproduce the beneficial effects of trace water while avoiding lithium anode side reactions; iii) Third, adaptive composite catalytic systems should be developed to allow mediators to switch active forms across different charge states or potential windows, enabling functional self‐matching between discharge and charge; iv) Fourth, systematic elucidation of RM‐electrolyte‐electrode ternary coupling mechanisms is required. Through in situ or operando characterization of discharge‐product morphology evolution, solution intermediates, and electron‐transfer kinetics, reaction processes can be visualized and quantitatively modeled. Such understanding will provide theoretical and methodological foundations for stability and reproducibility under scalable and engineering‐relevant conditions.

## 4 Novel Catalyst Systems and Advanced Design Strategies

Over the past decade, research on cathode catalysts for LOBs has evolved from single carbon‐based materials to TM compounds, and further to composite/derived porous architectures as well as, more recently, single‐atom/atomically defined catalytic sites [234]. The research focus has gradually shifted from simply increasing conductivity and specific surface area to synchronously optimizing the kinetics and selectivity of both ORR and OER through electronic‐structure regulation, surface‐chemistry engineering, and interface/pore‐structure design. Meanwhile, the integration of in situ/quasi‐in situ characterization with first‐principles calculations has driven a transition from empirically driven approaches toward mechanism‐guided design.

### 4.1 Single‐Atom Catalysts (SACs)

SACs anchor metal atoms on support surfaces in an atomically dispersed manner, achieving the theoretical limit of metal utilization and a highly uniform distribution of active sites, and have therefore attracted extensive attention in LOBs cathode catalysis in recent years [235]. Compared with nanoparticle or bulk catalysts, SACs can effectively avoid activity loss caused by aggregation and can markedly lower kinetic barriers for ORR and OER by finely tuning the local electronic structure of single‐atom centers, thereby improving energy efficiency and cycling reversibility. More importantly, since single‐atom sites are highly sensitive to their coordination environments, SACs serve as ideal model systems for probing reaction‐pathway regulation and structure‐performance relationships [236]. In terms of support types, current SAC research in LOBs mainly focuses on two categories: i) carbon‐based supports and ii) non‐carbon TM‐compound supports.

#### 4.1.1 Carbon‐Supported SACs

In LOBs research, SACs were first and most widely applied on carbon‐based supports such as N‐doped graphene, carbon nanotubes, and porous carbon [237, 238]. These supports offer high specific surface area, excellent conductivity, and abundant coordination sites, enabling stable formation of local structures such as M‐N_4_, M‐N_3_, or M‐O_4_. Systematic studies show that single atoms of TMs such as Fe, Co, Ni, and Mn can optimize O_2_ adsorption, activation, and dissociation by modulating d‐orbital electron filling, thereby significantly enhancing ORR/OER kinetics.

**FIGURE 11:**
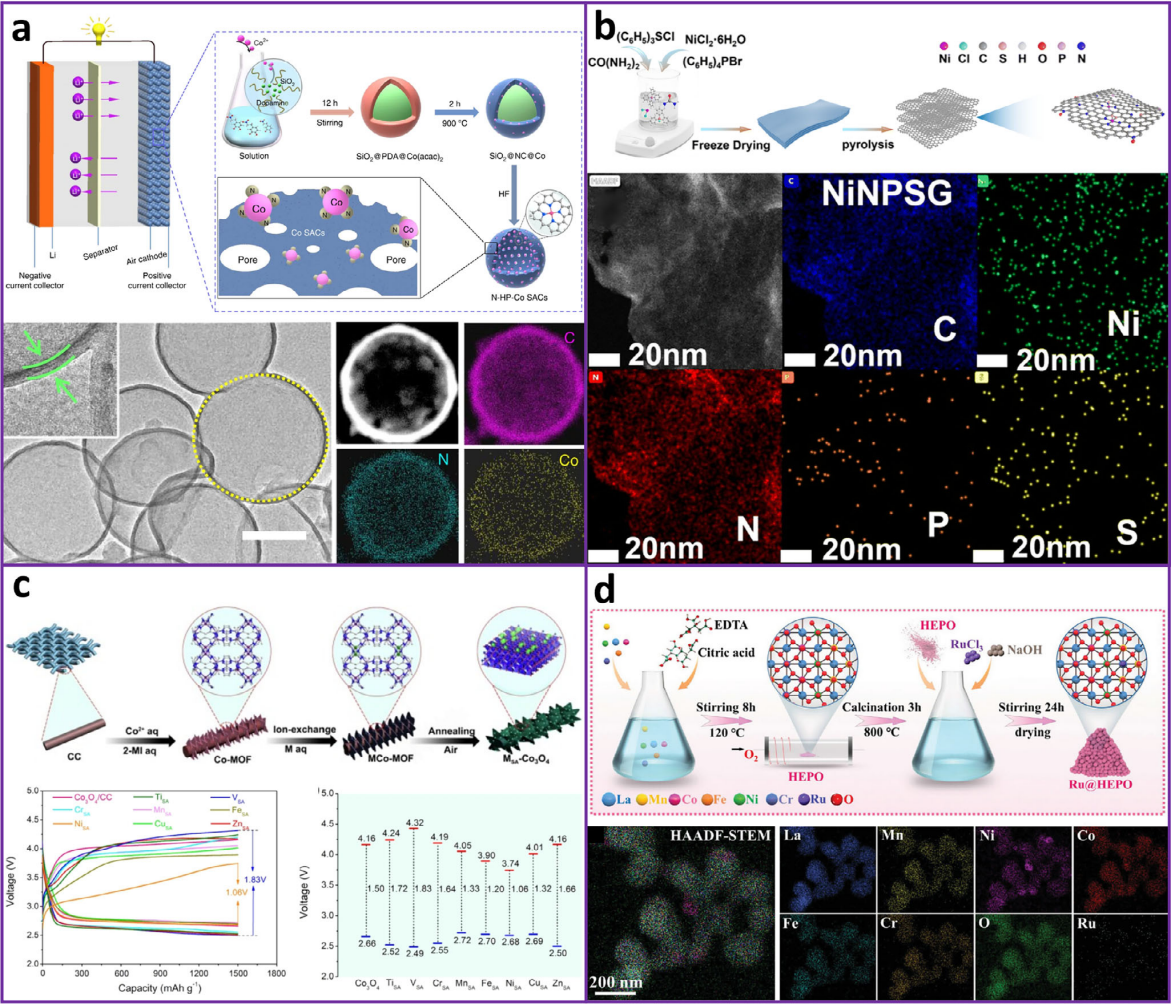
(a) Schematic illustration showing the synthesis of N‐HP‐Co SACs and SEM images of N‐HP‐Co SACs and the corresponding elemental maps. Reproduced with permission [75]. Copyright 2020, Springer Nature. (b) Schematic of the structural design of NiNPSG via one‐pot pyrolysis and the corresponding EDS mapping images of NiNPSG. Reproduced with permission [243]. Copyright 2025, Royal Society of Chemistry. (c) Schematic diagram of the MSA‐Co_3_O_4_/CC synthesis process and terminal discharge/charge potential and overpotential of different MSA‐Co_3_O_4_/CC. Reproduced with permission [246]. Copyright 2022, Elsevier. (d) Schematic diagram of the preparation process of Ru@HEPO and the corresponding elemental mapping images of Ru@HEPO. Reproduced with permission [248]. Copyright 2024, Wiley.

During discharge, carbon‐supported SACs not only accelerate reaction rates but also substantially influence the nucleation and growth behavior of Li_2_O_2_ [239]. For example, isolated Co single atoms can regulate the spatial distribution of active sites and induce the formation of micrometer‐scale flower‐like Li_2_O_2_ during discharge, thereby avoiding electrode blockage caused by dense deposition (Figure 11a) [75]. During charging, single‐atom sites more strongly favor a single‐electron oxidative decomposition pathway of Li_2_O_2_, significantly lowering the charging overpotential and accelerating kinetics. Compared with conventional noble‐metal nanocatalysts, such systems exhibit clear advantages in both energy efficiency and cycle life, highlighting the critical role of single‐atom‐support electronic coupling in regulating oxygen‐electrode reaction pathways.

The value of SACs lies not only in lower overpotential, but also in their ability to actively regulate the spatial distribution and accumulation behavior of discharge products. This ability to extend from catalytic reactions to product morphology engineering represents one of the core advantages that distinguish SACs from conventional catalysts.

#### 4.1.2 Reaction‐Pathway Reconstruction: Shifting from 2e^−^ ORR to 4e^−^ Process

Beyond regulating Li_2_O_2_ morphology, single‐atom sites can fundamentally alter the discharge reaction pathway. It has been reported that by precisely constructing specific coordination environments (e.g., embedding an asymmetric CoN_3_ motif into graphene) [240], the conventional 2e^−^ oxygen reduction pathway can be converted to a direct 4e^−^ pathway, shifting the discharge product from Li_2_O_2_ to LiOH. Compared with indirect routes that rely on Li_2_O_2_ hydrolysis, this direct electrochemical process significantly reduces the accumulation of reactive oxygen intermediates and effectively suppresses side reactions involving the electrolyte and carbon support, thereby markedly enhancing system stability [241]. Theoretical analyses further indicate that specific single‐atom sites can simultaneously strengthen adsorption and protonation of key intermediates, lower the O─O bond cleavage barrier, and thus benefit the pathway both thermodynamically and kinetically.

This finding shows that reaction‐pathway reconstruction enabled by electronic‐structure design is an important breakthrough through which SACs can transcend conventional catalytic strategies in LOBs. Changes in reaction pathways are often more decisive than activity enhancement. SACs offer the possibility of pathway‐selective regulation at the atomic scale, opening new design space for constructing LOBs with low parasitic reactions and high stability.

#### 4.1.3 Asymmetric Coordination and Multi‐Heteroatom Synergy

Traditional SAC designs often center on symmetric M‐N_4_ motifs [242], but increasing evidence suggests that introducing multiple heteroatoms (e.g., N, P, and S) to construct asymmetric, highly coordinated environments can further enhance catalytic performance (Figure 11b). Such multi‐heteroatom synergistic coordination can markedly tune local d‐orbital energy‐level distributions, optimize the adsorption‐desorption balance of oxygen intermediates, and increase the exposure and utilization of active sites [243]. In LOBs, these asymmetric single‐atom systems typically deliver higher discharge/charge capacities and longer cycle life, indicating that fine control of the local chemical environment is more critical than simply increasing metal species or loading. Future research on single‐atom catalysis should not be limited to finding the right metal, but should focus on constructing the right coordination asymmetry, enabling precise matching between electronic structure and reaction requirements.

#### 4.1.4 Non‐Carbon Supports and Oxygen‐Vacancy Synergy

Beyond carbon supports, non‐carbon TM compounds (e.g., TiO_2_, CeO_2_, and MXenes), owing to their tunable oxygen vacancies and surface defects, are considered ideal platforms for stabilizing single atoms [244, 245]. Synergy between oxygen vacancies and single‐atom centers can effectively tune the adsorption energies of key intermediates such as O_2_^−^ and LiO_2_ lower reaction barriers, and thereby “soften” ORR/OER pathways.

By systematically introducing different TM single atoms onto the same support while keeping the macroscopic structure identical, one can clearly distinguish atomic‐scale electronic effects from morphological factors. For example, in a series of single‐atom‐modified Co_3_O_4_ nanosheet arrays (MSA‐ Co_3_O_4_/CC; M = Ti, V, Cr, Mn, Fe, Ni, Cu, Zn), differences in catalytic performance were found to arise mainly from atomic‐scale reaction barriers and intermediate adsorption characteristics rather than macroscopic structural variations (Figure 11c) [246]. This provides an important methodological reference for rational screening and mechanistic comparison of SACs.

#### 4.1.5 Single Atoms on High‐Entropy Supports

In recent years, high‐entropy materials, owing to their multicomponent‐tunable local chemical environments and strong electronic coupling characteristics, have emerged as frontier platforms for hosting single‐atom active centers [247]. Pioneering studies have demonstrated that single‐atom Ru catalysts based on high‐entropy perovskite oxides La(Mn_0.2_Co_0.2_Fe_0.2_Ni_0.2_Cr_0.2_)O_3_ (Ru@HEPO) can construct stable atomic‐scale Ru‐O‐M (M = Mn, Co, Fe, Ni) bridge bonds by incorporating noble‐metal single atoms into high‐entropy perovskite oxides, thereby enabling rapid electron transfer from Ru to the HEPO host. This promotes effective redistribution of electron density at the catalytic interface and significantly enhances interfacial charge‐transfer kinetics and bifunctional electrocatalytic activity. In addition, strong electronic coupling between Ru and Mn facilitates hybridization between Mn 3d and O 2p orbitals, enhancing chemical affinity toward LiO_2_ intermediates and lowering oxygen‐electrode reaction barriers, thereby demonstrating the substantial potential of atomic‐level electronic‐structure regulation for high‐performance LOBs (Figure 11d) [248]. High‐entropy supports provide SACs with a programmable local environment, enabling single‐atom catalysis to advance from site‐level regulation toward atomic‐scale interface engineering, a concept with broad potential for extension to complex electrochemical systems.

Table 3 summarizes key performance metrics of representative SACs in LOBs, including initial discharge capacity, overpotential, current density, and cycling stability. Overall, although different metal centers, coordination structures, and support types exert markedly different influences on performance, general trends indicate that fine coordination control and strong electronic coupling typically correspond to lower overpotentials, asymmetric or multicenter structures are more conducive to improved cycling stability, and atomic‐scale interface engineering can enable long‐term stable operation at moderate current densities.

**TABLE 3:** Applications for Representative SACs in LOBs.

Material	First Discharge Capacity (mAh g^−1^@mA g^−1^)	Overpotential (V)	Current Rate (mA g^−1^)	Cycling performance	Refs.
Pt_1_Pd/C	8230@100	0.69	500	600 h	[249]
RuPt NPs@RuPt‐N‐C	16134@200	0.43	200	209 cycles (1045 h)	[250]
Co‐SA‐rGO Co‐N_4_	12760@200	1.33	200	220 cycles	[251]
CoN_3_ SACs	—	0.51 (COP)	300	3500 h	[252]
Pd SAs/NC	10000@200	0.24 (COP)	500	250 h	[234]
Ni‐NG SAC	24248@200	—	300	>500 cycles	[42]
CoSAs‐NHCS	3.59 mAh@500	0.74	200	>60 cycles	[253]
RuSA‐MnCo_2_O_4_	—	—	200	106 cycles	[254]
Ru@HEPO	12275@50	0.52	300	345 cycles	[248]
Ru SACs/OCS	—	—	100	2000 h	[255]
Ni_2_‐N/rGO	16000@200	1.08	200	225 cycles	[256]
Pt HEA@N‐C	12917.4@200	0.27	400	240 cycles	[257]
Nb‐SA@Co_3_O_4_/h‐PANI	8959@200	0.77	400	182 cycles (728 h)	[258]

SACs exhibit significant advantages in lowering overpotentials, improving reaction reversibility, and enhancing cycling stability through precise regulation of active‐site coordination environments and electronic structures. Nevertheless, further development still faces several key challenges, including the risk of single‐atom migration and aggregation under high loading or long cycling, unclear structural evolution and reaction mechanisms of single‐atom sites under practical operating conditions, and complex interactions among single atoms, electrolytes, and discharge products that may introduce new side‐reaction pathways.

### 4.2 MOFs‐Derived Catalysts and In Situ Transformation Strategies

MOFs offer distinctive advantages for designing cathode catalysts in LOBs, owing to their highly ordered pore architectures, tunable metal nodes, and uniformly distributed coordination environments [259]. Through approaches such as pyrolysis, sulfidation, doping, or in situ transformation, MOFs can be converted into derived catalysts featuring high specific surface areas, hierarchical porosity, continuous conductive networks, and atomically dispersed active centers. While inheriting the structural programmability of MOFs, these materials markedly improve electrical conductivity and chemical stability, thereby providing a highly tunable materials platform for bifunctional ORR/OER catalysis [260]. Existing studies indicate that the core value of MOF‐derived catalysts lies not in the novelty of the material source, but in their role as intrinsic templates for synergistically regulating structure, electronic properties, and reaction behavior, enabling engineering‐oriented design of complex catalytic systems.

#### 4.2.1 MOF‐Derived Hierarchical Porosity and Conductive Networks

**FIGURE 12:**
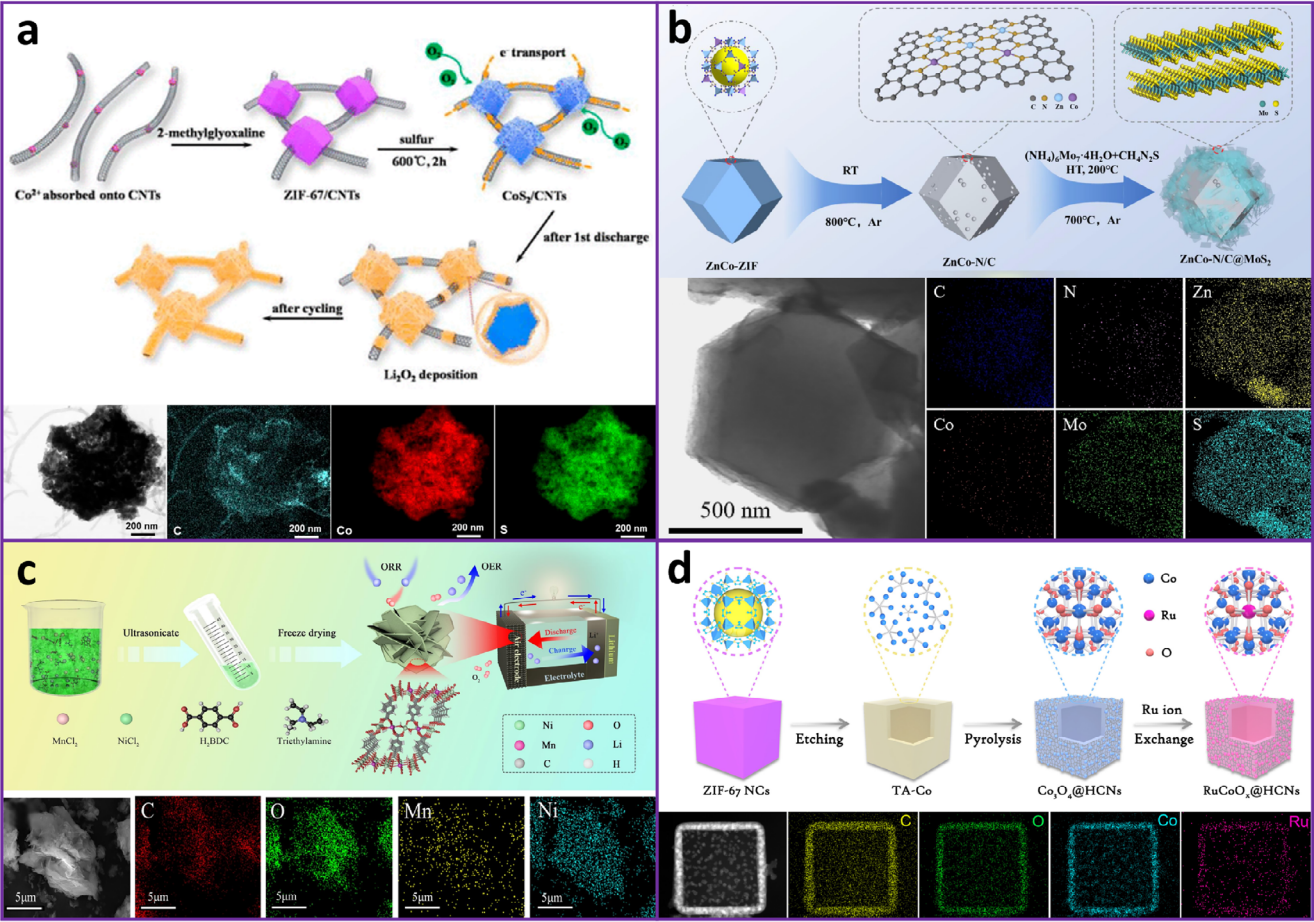
(a) Illustration of fabrication procedure for CoS_2_/CNTs composites and EDX mapping images of C, Co, and S elements of ZIF‐derived CoS_2_/CNTs composites. Reproduced with permission [261]. Copyright 2020, American Chemical Society. (b) Schematics for the synthesis of ZnCo‐N/C@MoS_2_ and elemental mapping images. Reproduced with permission [264]. Copyright 2025, Wiley. (c) Schematic illustration of the synthesis process of NiMn‐based MOF and elemental mapping images. Reproduced with permission [267]. Copyright 2023, Elsevier. (d) Schematic diagram of the formation process of RuCoO_x_@HCNs and elemental mapping images. Reproduced with permission [114]. Copyright 2025, American Chemical Society.

Converting MOFs into composite architectures with hierarchical pores and high conductivity via controlled carbonization or sulfidation is an effective strategy to enhance bifunctional oxygen electrocatalysis in LOBs. A typical design concept is to use MOFs as sacrificial templates to construct hollow or nanocage‐like structures, while introducing 1D conductive scaffolds (e.g., CNTs) to form 3D interconnected networks. For example, using ZIF‐67 as the precursor, a controllable sulfidation route can produce internally porous CoS_2_ nanocages, which are further integrated with CNTs to build a 3D interconnected conductive network (CoS_2_/CNTs, Figure 12a) [261].

Functionally, such architectures exhibit pronounced synergy. The porous nanocage structure provides abundant exposed edge/corner sites and interconnected channels, facilitating O_2_ diffusion, electrolyte infiltration, and intermediate conversion. The continuous conductive network compensates for the intrinsically insufficient conductivity of metal sulfides or oxides, significantly reducing electron‐transport resistance during ORR/OER processes. Structural synergy can guide more uniform and loosely packed deposition of Li_2_O_2_, thereby improving reaction reversibility and mitigating pore blockage [262]. Correspondingly, electrochemical performance is typically manifested as higher reversible capacity, lower polarization, and stable mid‐term cycling life.

For MOF‐derived systems, pore architecture and conductive network are not a simple additive relationship. Rather, it determines whether the overall reaction is limited by kinetics or by mass transport. In LOBs, only when electron transport and product‐deposition pathways are simultaneously optimized can bifunctional catalytic activity be translated into sustainable cycling performance.

#### 4.2.2 Multielement Synergistic Regulation

In MOF‐derived catalysts, multidimensional regulation of the coordination environment and electronic structure at metal‐support interfaces by introducing hetero‐elements is a key strategy for balancing bifunctional ORR/OER activity [263]. A representative approach is to use heterometals (e.g., Zn) to stabilize and tune single‐atom or quasi‐single‐atom active centers such as Co‐N_x_ while constructing an outer 2D TM sulfide coating layer (e.g., MoS_2_) to form a multicenter coupled interface (Figure 12b) [264]. In such a “bidirectional” structure‐electronics regulation scheme, different components assume distinct functions. Heterometals can tune the local coordination and charge distribution of Co‐N_x_, enhancing ORR activity and stability. The MoS_2_ coating introduces new metal‐nitrogen coupled centers, improves overall conductivity, and strengthens OER activity. Multicenter synergy can precisely regulate the generation and desorption behavior of *LiO_2_ intermediates, enabling more uniform discharge products that are easier to decompose. This strategy often simultaneously delivers low overpotential, high specific capacity, and long cycle life, highlighting the unique advantage of MOF‐derived systems in constructing complex multicenter catalytic environments.

The essence of multielement synergistic regulation is not “the more elements, the better” [265], but whether a functionally complementary and mutually constrained network of active centers can be established. The advantage of MOF‐derived systems is that their atomically distributed nature makes such fine regulation structurally feasible.

#### 4.2.3 Bimetal‐Node Modulation and Orbital Engineering

Beyond post‐treatment derivation, introducing a second metal node directly during MOF construction to optimize catalytic activity by tuning TM d‐orbital occupancy has also become an important direction in recent years [266]. A typical strategy is to introduce a small amount of heterometal ions into a monometallic MOF, such as the Ni/Mn bimetallic MOF (NiMn‐MOF) approach, where trace Mn ions are incorporated into Ni‐MOF to precisely tune the e_g_ orbital occupancy of Ni sites (Figure 12c) [267].

Theoretical analyses suggest that adjacent heterometal ions can induce redistribution of 3d electrons in the primary active metal, enhancing hybridization between metal d orbitals and O 2p orbitals, thereby optimizing the adsorption energy of key intermediates such as *LiO_2_ and lowering reaction barriers [268]. Benefiting from this orbital‐engineering effect, such bimetallic MOFs often exhibit ultrahigh specific capacities and significantly extended cycle life in LOBs.

The key implication of this line is that MOFs are not only structural templates, but also programmable electronic‐structure platforms. By finely tuning e_g_/t_2g_ orbital occupancy, conventional empirical adsorption‐energy regulation can be elevated to design principles with clear physical meaning.

#### 4.2.4 In Situ Atom‐Level Transformation

Introduction of atomically dispersed hetero‐active centers into MOF/oxide‐derived structures via in situ doping or substitution has gradually become a key strategy for improving the reversibility of LOBs [269]. Such methods typically realize atom‐level incorporation of heterometals at specific lattice sites, creating hetero‐bonded units with pronounced structural asymmetry (e.g., M‐O‐M’). One reported example is an in situ Ru‐doped Co_3_O_4_‐derived heterostructure (RuCoO_x_@HCNs), where atomic substitution at octahedral sites of Co_3_O_4_ constructs structurally asymmetric Ru‐O‐Co units (Figure 12d) [114].

These atomically heterogeneous structures offer multiple advantages [270]. Strong electronic coupling can reshape local electron density and d‐orbital energy levels, promoting O_2_ activation and its conversion to superoxide species. They exhibit higher affinity toward key intermediates such as LiO_2_, thereby inducing Li_2_O_2_ deposition with specific morphologies. Directionally deposited discharge products are more readily decomposed during charging, thus markedly lowering polarization and suppressing side reactions [271]. Accordingly, such systems often deliver extremely low polarization voltages and ultralong operational lifetimes, significantly outperforming conventional single‐phase oxides or common MOF‐derived materials.

The true value of in situ atom‐level transformation lies in directly linking catalytic site design with discharge‐product morphology control. For LOBs, the ability to actively steer the deposition and decomposition pathways of Li_2_O_2_ may be more critical than simply increasing intrinsic activity.

MOF‐derived catalysts, benefiting from strong structural programmability, highly tunable active centers, and pronounced interfacial synergistic effects, have become an important class of materials for constructing high‐performance LOBs cathodes, as shown in Table 4. From hierarchical porosity and conductive‐network construction to multimetal synergy and orbital engineering, and further to in situ generation of atomically heterogeneous centers, existing studies consistently demonstrate that MOF‐derived strategies provide a full‐chain design paradigm spanning structure‐electronics‐reaction behavior for complex electrocatalytic reactions.

**TABLE 4:** Applications for representative MOF‐derived materials in LOBs.

Material	Initial Discharge Capacity (mAh g^−1^@mA g^−1^)	Current Rate/Capacity limit (mA g^−1^/mAh g^−1^)	Overpotential (V)	Cycling performance (Cycles)	Refs.
MnCo‐MOF‐74	11150@200	200/1000	1.26	44	[272]
3DP‐NC‐Co	1124@0.05 mA cm^−2^	0.1 mA cm^−2^/1 mAh	—	150	[273]
Co_9_S_8_@CPNs	7000@50	100/500	1.057	110	[274]
CuO‐CuCo_2_O_4_	6844@100	400/1000	1.15	111	[275]
N‐HC@CoO‐Co_3_O_4_	24265@300	300/500	0.61	112	[193]
Co‐Cage	15500@500	500/1000	1.24	132	[276]
NiCo‐LDH/MnO_2_	13380@100	100/500	0.63	162	[277]
FeN_x_‐HDC@Ru	3080@100	200/500	0.93	90	[278]
Co_3_O_4_@NiCo_2_O_4_	11672@100	500/1000	1.012	280	[279]
MnOC@CC	22838@200	200/500	1.17	53	[280]
Na‐Pb‐MOF	6247@100	100/1000	0.52	140	[263]
[Cu_1.5_Ni_1.5_(HHTP)_2_]_1_‐(G‐OH)_1_	12542@50	50/500	1.1	40	[281]
NiCo_2_S_4_@NC	11618@100	200/1000	1.85	104	[282]
CoFe‐LDH@CNTs	32800@500	500/500	0.87	176	[283]
Co_3_O_4_@CuCo_2_O_4_	7015.5@100	400/1000	1.47	146	[284]
Co_3_O_4_/RuO_2_	11304@100	500/1000	0.998	387	[285]

### 4.3 Composite Heterostructures and Interfacial Synergistic Effects

Heterostructured catalysts, by constructing interfaces between different phases or chemical components, can simultaneously regulate electronic structure, charge‐carrier transport, and reactant adsorption behavior, and are therefore widely regarded as an effective strategy for enhancing the bifunctional ORR/OER activity of LOBs cathodes [286]. Compared with single‐phase materials, heterointerfaces not only provide richer local active sites but also trigger charge redistribution and band structure modulation, thereby lowering the kinetic energy barriers for key intermediate transformations. Meanwhile, rational 3D structural design can guide the macroscopic deposition morphology of Li_2_O_2_, alleviating pore blockage and reaction irreversibility [287]. Consequently, heterostructures in LOBs often exhibit synergistic gains arising from the combination of interfacial electronic effects and structural mass‐transport effects.

#### 4.3.1 Oxide/Oxide Heterostructures

**FIGURE 13:**
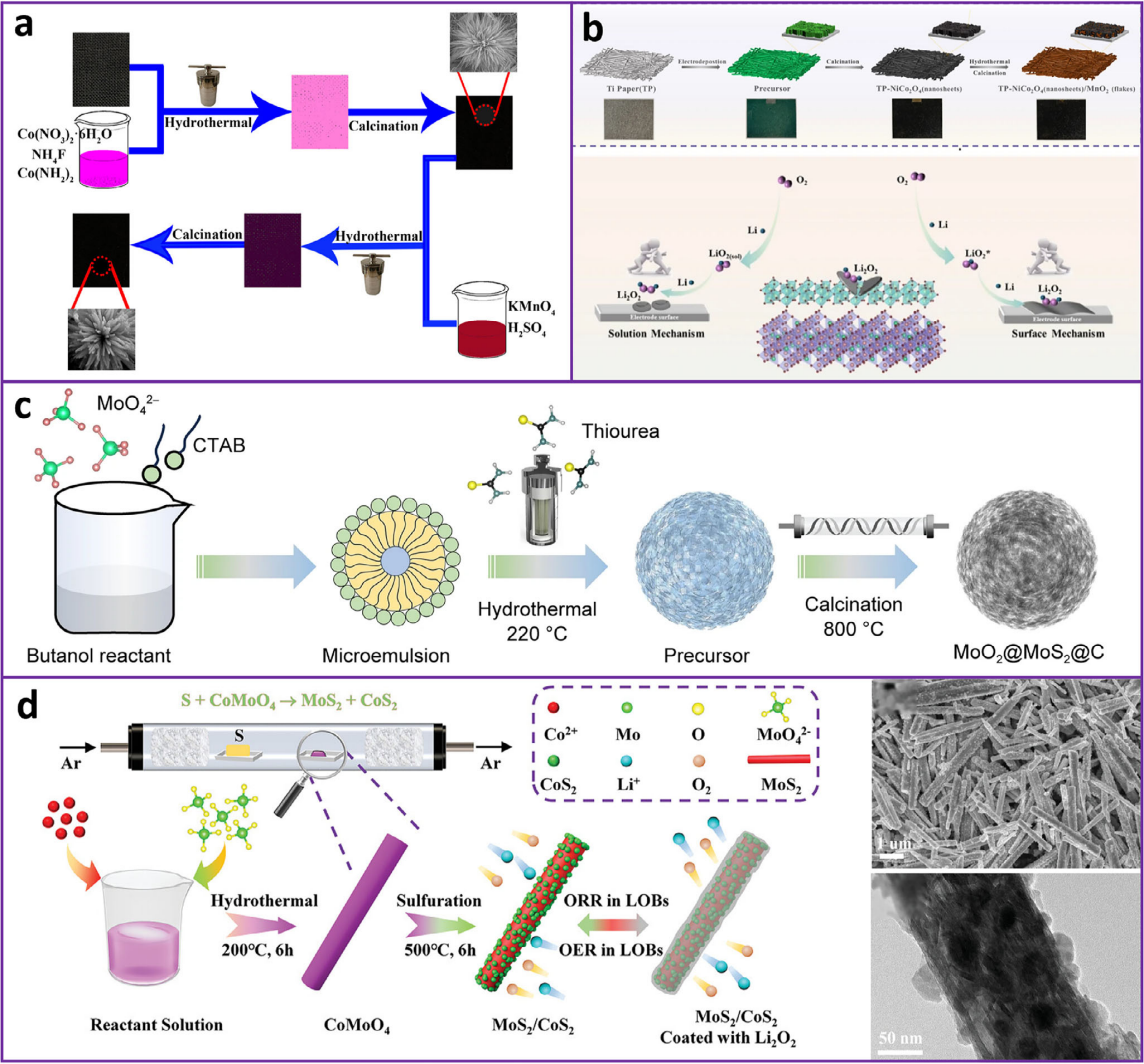
(a) Schematic illustration of the two‐step hydrothermal synthesis of 3DOS‐Co@Mn heteromatrix on carbon cloth. Reproduced with permission [289]. Copyright 2021, American Chemical Society. (b) A Flow illustration of TP‐NCO/MO preparation and schematic reaction mechanism. Reproduced with permission [292]. Copyright 2024, Springer Nature. (c) A Preparation schematic illustration of MoO_2_@MoS_2_@C. Reproduced with permission [293]. Copyright 2024, Springer Nature. (d) Schematic illustration of the synthesis of MoS_2_/CoS_2_ with the proposed lithium storage mechanism. Reproduced with permission [296]. Copyright 2021, Wiley.

One representative strategy involves coupling two oxide phases while introducing 3D open architectures to simultaneously address reaction kinetics and mass transport [288]. Taking the Co_3_O_4_@MnO_2_ bifunctional heterostructured composite as an example, the 3D open framework provides low‐resistance pathways for electron/ion transport and gas diffusion, while the heterointerface enhances electron transfer and intermediate conversion efficiency during ORR/OER processes (Figure 13a) [289]. Such electrodes can deliver high initial discharge capacities and stable cycling performance in LOBs. More importantly, the 3D pore structure can induce more uniform and loosely packed deposition of the discharge product Li_2_O_2_, reducing the risk of pore blockage and thereby improving reversibility and cycle life.

The advantages of oxide heterostructures stem not only from higher activity, but also from their ability to intervene in the product deposition pathway [290]. For LOBs, interfacial catalysis and pore‐structure regulation must be regarded as a unified design problem. Only when enhanced interfacial kinetics and controllable Li_2_O_2_ deposition are simultaneously achieved can capacity and lifetime be improved in parallel.

#### 4.3.2 Mott‐Schottky Heterojunctions

Another more mechanism‐oriented heterostructure strategy involves constructing Mott‐Schottky heterojunctions, in which band bending and carrier redistribution are used to regulate interfacial charge behavior [291]. Carbon‐free, self‐supported NiCo_2_O_4_/MnO_2_ heterostructured electrodes applied in high‐performance LOBs can form high‐quality interfacial contact via nanosheet arrays, inducing pronounced interfacial band perturbation and electronic coupling effects (Figure 13b) [292]. Mechanistically, such interfaces can simultaneously optimize electron transfer and adsorption/desorption charge regulation, enabling ORR/OER to proceed along pathways closer to reversibility. Accordingly, these electrodes exhibit ultralong cycle life and low overall overpotentials, significantly outperforming conventional carbon‐based or single‐phase oxide electrodes.

The unique value of Mott‐Schottky heterojunctions lies in elevating interfacial effects from empirical superposition to a designable band‐engineering problem [292]. Future attention should focus on how band structures correlate with the adsorption free‐energy windows of key LOBs intermediates, and whether such charge‐allocation mechanisms remain stable under high surface coverage and deposit formation.

#### 4.3.3 Homologous Heterostructures

Beyond coupling dissimilar materials, homologous heterostructures, which construct multiphase coexistence within the same chemical system (such as MoO_2_/MoS_2_@C spherical catalysts), often achieve both lower interfacial energy barriers and more continuous charge‐transport pathways (Figure 13c) [293]. Integrating homologous heterostructured phases can promote LiO_2_ formation and conversion during discharge while lowering overpotential, and accelerate Li_2_O_2_ decomposition during charging, thereby enhancing reversibility [294]. The induced synergistic electronic effects at multiphase interfaces play a key role in tuning intermediate adsorption energies and improving electron‐transport efficiency.

The core of homologous heterostructure design is not “the more phases, the better”, but rather functional division of labor combined with low‐loss interfaces [295]. For LOBs, more appropriate evaluation criteria should assess whether interfaces maintain structural continuity and electrochemical stability during cycling, and whether interface degradation caused by phase transformation or dissolution is avoided.

#### 4.3.4 Sulfide Heterostructures

TM sulfides generally possess higher intrinsic electrical conductivity, making them suitable as carrier platforms for enhancing interfacial charge transport. Taking CoS_2_/MoS_2_ heterostructures as a representative example, the metallic nature and ORR activity of CoS_2_ complement the layered structure and OER potential of MoS_2_ after coupling (Figure 13d) [296]. The heterointerface further enhances rapid electron migration between the two phases, improving overall conductivity and structural stability [297]. More importantly, such interfaces can induce preferential epitaxial growth or more controllable deposition of Li_2_O_2_ on the electrode surface, thereby mitigating nonuniform accumulation and pore blockage. As a result, batteries exhibit higher discharge capacities, improved rate capability, and longer cycling stability.

The advantages of sulfide heterostructures typically arise from the dual effects of enhanced conductivity and interface‐induced deposition control, while their main challenge lies in the chemical stability of sulfides under strongly oxidative environments and high charging potentials. Future studies should evaluate interfacial catalytic gains in parallel with material durability constraints [298], rather than relying solely on initial activity as the terminal performance metric.

Heterostructured catalysts, through interfacial synergistic effects and multiphase electronic regulation, provide a practical and broadly applicable materials‐design strategy for achieving efficient bifunctional electrocatalysis in LOBs. By introducing charge redistribution, band modulation, and electronic coupling at phase interfaces, heterostructured catalysts can construct highly active and stable oxygen‐electrode reaction platforms [299]. In LOBs, interfacial effects exert synergistic influences across three key dimensions: i) Interfacial electronic‐structure regulation optimizes the adsorption energies of ORR/OER intermediates, thereby significantly lowering kinetic barriers for oxygen‐electrode reactions; ii) Enhanced structural integrity and conductive networks ensure efficient electron/ion transport while mitigating structural collapse and interfacial deactivation commonly observed in single‐phase materials; iii) Controlled product morphology and accumulation behavior, achieved by inducing uniform deposition and reversible decomposition of Li_2_O_2_, effectively suppress electrode blockage and the continuous accumulation of side reactions.

From oxides and sulfides to homologous heterostructures and Mott‐Schottky interfacial systems, existing studies consistently demonstrate that refined heterointerface design has become one of the key pathways for overcoming bifunctional electrocatalytic bottlenecks in LOBs.

### 4.4 High‐Entropy Catalysts

Among the emerging catalyst‐design strategies for nonaqueous LOBs, high‐entropy materials are attracting increasing attention because they address a central contradiction of the oxygen cathode: the same catalyst must simultaneously facilitate O_2_ reduction during discharge, enable Li_2_O_2_ decomposition during charge, and remain stable in the presence of reactive oxygen intermediates and insulating solid products [300]. In aprotic LOBs, the cathode reaction is not a simple one‐step ORR/OER process but a coupled sequence involving O_2_ adsorption, LiO_2_ formation, solution/surface pathway competition, Li_2_O_2_ nucleation and growth, and its subsequent oxidation during charging. These multistep processes are further complicated by electrolyte decomposition, carbon corrosion, Li_2_CO_3_ accumulation, and singlet‐oxygen‐related parasitic chemistry, all of which make the cathode the dominant bottleneck for energy efficiency and cycle life [301]. Therefore, catalyst design in LOBs must move beyond maximizing a single descriptor and instead target multifunctional regulation of intermediate adsorption, reaction pathway distribution, discharge‐product morphology, and interfacial stability.

From this perspective, the importance of high‐entropy catalysts in LOBs is more fundamental than a simple “activity enhancement” narrative. Traditional single‐component catalysts or low‐component mixed oxides often optimize only one elementary step, such as O_2_ adsorption, LiO_2_ stabilization, or Li_2_O_2_ oxidation, but they rarely balance all of them simultaneously. By contrast, high‐entropy systems introduce a broad distribution of local atomic configurations, coordination environments, and electronic states, making it possible to host multiple types of catalytic sites within one phase. This feature is especially attractive for LOB cathodes because discharge and charge do not necessarily favor the same adsorption strength or the same reaction microenvironment. High‐entropy design therefore offers a pathway to decouple, or at least relax, the classical trade‐off between discharge activity, charge reversibility, and structural durability. More broadly, recent work in electrocatalysis has also emphasized that high‐entropy materials can help overcome the limitations imposed by conventional scaling relations by creating chemically diverse local motifs rather than a single average active site.

#### 4.4.1 Advantages of High‐Entropy Catalytic Materials

The relevance of high‐entropy catalysis to LOBs stems from the unique requirements of the oxygen electrode. First, the discharge reaction in aprotic LOBs can proceed through both surface‐mediated and solution‐mediated pathways, and the relative contribution of these routes determines whether Li_2_O_2_ forms as thin passivating films or as larger particles with better accommodation in porous cathodes. Second, the charging process is highly sensitive not only to Li_2_O_2_ oxidation kinetics but also to side reactions involving electrolyte and carbon components, which are often aggravated at elevated charge potentials. Third, the repeated precipitation and decomposition of electronically insulating Li_2_O_2_ continuously reconstruct the cathode interface, so catalytic stability cannot be separated from morphological tolerance. These features imply that an ideal LOBs cathode catalyst must offer not merely high intrinsic ORR/OER activity, but also tunable adsorption energetics, pathway adaptability, and resistance to chemical/structural degradation [104].

High‐entropy materials are well matched to these demands for three reasons [302]. First, the coexistence of multiple principal elements generates a wide spectrum of d‐band positions, charge distributions, and metal‐oxygen bonding environments, allowing a single catalyst surface to interact differently with O_2_, LiO_2_, Li_2_O_2_, and parasitic intermediates. Second, multicomponent oxides and alloys often contain redox‐flexible cations or composition‐dependent electronic heterogeneity, which is beneficial for bifunctional catalysis across both discharge and charge. Third, configurational entropy tends to stabilize single‐phase or quasi‐single‐phase structures against phase segregation and severe reconstruction, which is valuable under the strongly nonequilibrium environment of LOB cycling. In other words, the significance of high‐entropy catalysts in LOBs lies not only in providing “more active sites”, but in enabling a more distributed and adaptable catalytic landscape that better matches the multiscale complexity of oxygen electrochemistry.

#### 4.4.2 Mechanistic Roles of High‐Entropy Effects in LOBs

A more rigorous discussion of high‐entropy catalysts in LOBs should distinguish between three levels of effect: electronic‐structure broadening, local structural distortion, and reaction‐interface regulation.

1. Electronic‐Structure Broadening: At the electronic level, multicomponent occupation produces a distribution rather than a single value of adsorption energies. For LOB cathodes, this is important because excessively strong adsorption may hinder Li_2_O_2_ decomposition during charge, whereas excessively weak adsorption may fail to stabilize discharge intermediates and control Li_2_O_2_ nucleation. A broadened energetic landscape can therefore support multiple elementary steps within the same catalyst, reducing the likelihood that one reaction step becomes optimal only at the expense of another.
2. Local Structural Distortion and Defect Engineering: at the structural level, high configurational entropy is commonly accompanied by local lattice distortion, asymmetric coordination, defect redistribution, and strain effects. In oxide catalysts, these factors can tune metal‐oxygen covalency, oxygen‐vacancy chemistry, and the accessibility of different cation sites. For LOBs, such changes are closely related to how LiO_2_ adsorbs, whether Li_2_O_2_ grows through surface or solution routes, and how readily the discharge product can be oxidized on charge. Importantly, these effects are not purely thermodynamic; they also modify interfacial kinetics and product morphology. Thus, high entropy should not be understood as an abstract compositional feature, but as a practical method for constructing reaction microenvironments that are simultaneously heterogeneous and structurally persistent.
3. Interfacial Reaction Regulation and Suppression of Side Reactions: high‐entropy catalysts can regulate the balance between desired oxygen redox and undesired parasitic chemistry. Because charging in LOBs is particularly vulnerable to electrolyte oxidation and carbon‐side corrosion, any catalyst that lowers charge overpotential while preserving O_2_ evolution selectivity is intrinsically more valuable than one that merely boosts discharge capacity. This is why the role of high‐entropy catalysts in LOBs should be judged not only by initial capacity or rate capability, but by whether they improve round‐trip efficiency, suppress byproduct accumulation, and extend limited‐capacity cycling under realistic current densities.

#### 4.4.3 Functional Differentiation and Comparison of Different High‐Entropy Catalytic Systems in LOBs

  - High‐Entropy Alloys (HEAs): Multisite Synergy and Reaction Pathway Regulation

**FIGURE 14:**
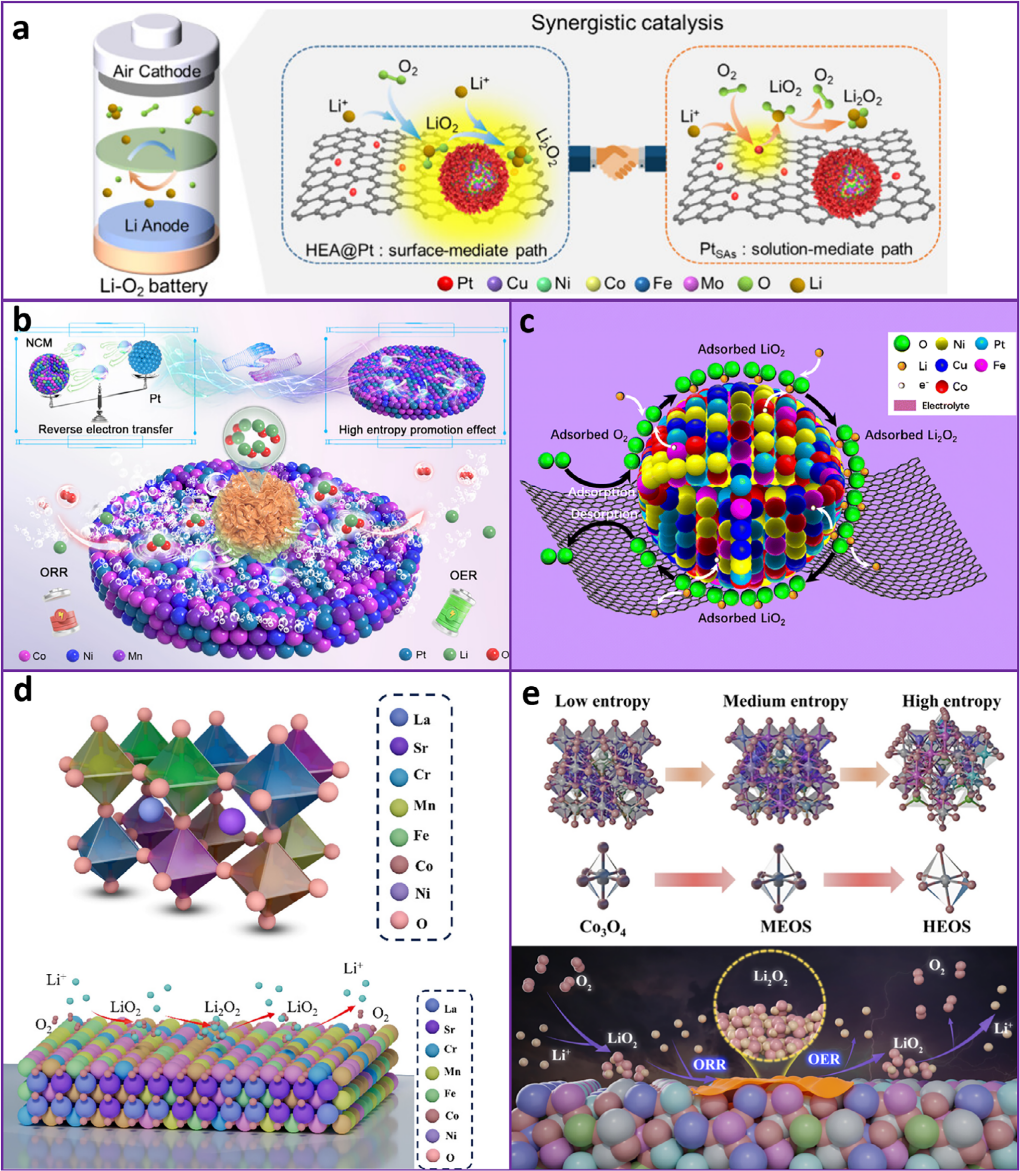
(a) Schematic diagram of the HEA@Pt synergistic catalysis mechanism. Copyright 2025, American Chemical Society [125]. (b) Schematic illustration of the underlying mechanism of the Pt HEA@N‐C catalyst. Reproduced with permission [257]. Copyright 2025, American Chemical Society. (c) Schematic diagram of the Li_2_O_2_ formation mechanism and electrochemical dynamics on the PtFeCoNiCu@rGO cathode. Reproduced with permission [303]. Copyright 2025, Royal Society of Chemistry. (d) Crystal structure model of perovskite LS(MFCCN)O_3_ and diagram showing the generation and breakdown of the discharge product Li_2_O_2_ on LS(MFCCN)O_3_. Reproduced with permission [304]. Copyright 2024, Wiley. (e) Crystal structure model of HEOS and Schematic diagram of the ORR and OER processes on the HEOS electrode. Reproduced with permission [305]. Copyright 2024, American Chemical Society.

HEAs are the most direct embodiment of the “multisite synergy” concept in LOBs catalysis. Their metallic nature favors rapid electron transport, while random near‐equimolar elemental mixing creates diverse surface ensembles for O_2_ adsorption and intermediate stabilization. A representative example is the dual‐site catalyst composed of Pt single atoms paired with HEA nanoparticles, where the HEA component was reported to favor adsorbed LiO_2_ while Pt single atoms promoted the soluble LiO_2_ pathway (Figure 14a) [125]. By tuning the relative contribution of these two pathways, the authors achieved accelerated ORR, suppressed parasitic reactions, an ultralow overpotential of 0.3 V, and cycling up to 470 cycles at 1000 mA g^−1^. This study is particularly important because it shows that, in LOBs, the value of HEAs may lie less in a simple increase of active‐site density and more in the deliberate balancing of different LiO_2_ formation/decomposition pathways. In addition, NiCoMn and Pt‐group metals (Pt, Ir, Ru) were synthesized into ultrafine α‐NiCoMn high‐entropy alloy nanoparticles via a one‐step Joule heating method and supported on N‐doped carbon (Figure 14b) [257]. The resulting material exhibits an unusual reverse electron transfer from NiCoMn to Pt, which modulates the d‐band center and optimizes the electronic structure. Meanwhile, the high‐entropy coordination environment provides diverse active sites, facilitating LiO_2_ intermediate adsorption and the reversible formation/decomposition of Li_2_O_2_.

Another representative HEA system is PtFeCoNiCu nanoparticles supported on reduced graphene oxide (Figure 14c) [303]. In this case, the conductive rGO scaffold worked together with the multicomponent alloy to improve the reversible formation/decomposition kinetics of Li_2_O_2_. The corresponding LOB delivered an initial discharge capacity of 13 949 mA h g^−1^, a low overpotential of 0.77 V, and stable cycling over 148 cycles under a limited capacity of 500 mA h g^−1^ at 100 mA g^−1^, with Li_2_O_2_ identified as the dominant discharge product and no obvious byproducts reported. Compared with isolated metallic nanoparticles, this type of HEA/carbon composite also highlights an important practical point: in LOBs cathodes, catalytic optimization must be integrated with electron transport and pore accessibility, because the catalyst alone cannot offset the transport penalties imposed by insulating discharge products.

Taken together, current HEA studies suggest that metallic high‐entropy catalysts are particularly promising when the design target is pathway redistribution, rapid interfacial electron transfer, and suppression of charge polarization. Their limitation, however, is that noble‐metal‐containing systems may raise cost and that the mechanistic origin of stability under long‐term oxidative conditions still requires deeper operando verification.

  - High‐Entropy Perovskite Oxides: Strain Engineering and Redox Flexibility

Compared with HEAs, high‐entropy perovskite oxides are more attractive from the viewpoint of structural tunability and redox chemistry. Their A‐site/B‐site compositional freedom enables simultaneous regulation of cation valence, metal‐oxygen covalency, and lattice distortion, all of which are closely linked to oxygen electrocatalysis [248]. In LOBs, this is valuable because oxide catalysts often need to mediate both ORR and OER while maintaining structural integrity under repeated Li_2_O_2_ deposition and removal. The strain‐rich high‐entropy perovskite (La_0.8_Sr_0.2_)(Mn_0.2_Fe_0.2_Cr_0.2_Co_0.2_Ni_0.2_)O_3_ is an illustrative example (Figure 14c) [304]. According to the reported study, the material showed high cationic dispersion and strain‐enhanced oxygen‐redox reactivity, and the LOBs using this catalyst delivered a discharge capacity of 17,078.2 mA h g^−1^ with a cycling life of 435 cycles.

The broader significance of high‐entropy perovskites is that they move high‐entropy catalysis in LOBs from the metallic‐surface paradigm toward an oxide‐framework paradigm. Here, entropy not only diversifies surface adsorption sites; it also modulates bulk‐surface coupling, defect chemistry, and lattice oxygen participation tendencies. This makes perovskite‐type high‐entropy oxides especially suitable as stable catalyst hosts or as platforms for coupling with single‐atom sites, defect engineering, and interface engineering. In this sense, high‐entropy perovskites may be viewed as a bridge between classical oxide electrocatalysts and next‐generation compositionally complex catalytic architectures for LOBs.

  - High‐Entropy Spinel Oxides: Local Coordination Asymmetry and Structural Stability

High‐entropy spinel oxides are particularly relevant to LOBs because spinel frameworks are already familiar oxygen‐electrode motifs, but entropy engineering introduces a qualitatively new degree of local structural asymmetry. In the recent study on high‐entropy spinel (Co_0.2_Mn_0.2_Ni_0.2_Fe_0.2_Cr_0.2_)_3_O_4_ (HEOS), the authors explicitly linked internal configurational entropy to controllable oxygen redox in LOBs (Figure 14d) [305]. The key point here is not only that multiple cations coexist, but that entropy alters the local coordination landscape of tetrahedral and octahedral sites, thereby influencing intermediate binding and the reversibility of Li_2_O_2_ chemistry. This line of work is important because it shows that for LOB cathodes, the catalytic role of high entropy can be embedded into a crystallographically well‐defined oxide framework instead of relying solely on alloy surface complexity.

Relative to HEAs, high‐entropy spinels may offer stronger resistance to structural collapse and easier compositional tuning of oxygen‐related defects, but they can also suffer from lower intrinsic conductivity. Therefore, their practical implementation in LOB cathodes will likely depend on conductive hybridization, nanoscale architecture control, and precise management of entropy–defect coupling. Even so, the emergence of HEOS‐type catalysts clearly indicates that high‐entropy design in LOBs is no longer limited to “adding more metals”; it is evolving into a framework‐level strategy for engineering oxygen‐redox microenvironments.

A comparison of the current literature reveals that the three main high‐entropy catalyst families play distinct but complementary roles in LOBs cathodes. HEAs are strongest in interfacial electron transfer and multisite pathway balancing; [125] high‐entropy perovskites are advantageous for redox‐flexible oxide catalysis and strain/defect engineering; high‐entropy spinels are promising for framework‐stable regulation of local coordination asymmetry and reversible Li_2_O_2_ chemistry [302]. Across these categories, the most convincing advances are not those reporting only higher initial capacity, but those demonstrating lower charge overpotential, cleaner Li_2_O_2_ reversibility, and longer limited‐capacity cycling under controlled conditions.

The real value of high‐entropy catalysts in LOBs lies in offering a design philosophy rather than merely a new material label. By constructing compositionally complex yet structurally stable catalytic environments, high‐entropy strategies provide a realistic route to reconcile the conflicting requirements of ORR activity, OER reversibility, and interfacial durability. Looking ahead, the most promising direction is likely not the isolated use of entropy alone, but its integration with single‐atom engineering, defect and strain control, conductive scaffold design, and operando‐guided descriptor development. Such a combination may ultimately enable LOB cathodes that are not only more active, but also more selective, more reversible, and more credible for practical operation.

### 4.5 Spin‐Regulated Oxygen Electrocatalysis

#### 4.5.1 Theoretical Background of Electron Spin Catalysis

Electron spin catalysis was initially proposed to explain the long‐standing kinetic bottlenecks in aqueous OER and ORR, with its theoretical foundation rooted in the fact that molecular oxygen has a triplet ground state (triplet O_2_), making spin conservation and spin‐flip processes unavoidable during reactions [306]. With the continuous advancement of research on the chiral‐induced spin selectivity (CISS) effect and magnetic catalysis, electron spin has gradually been recognized as a tunable reaction degree of freedom. This concept has begun to be introduced into nonaqueous LOBs systems and has demonstrated potential advantages that are highly compatible with the unique reaction characteristics of this system.

Unlike aqueous systems, cathode reactions in nonaqueous LOBs involve not only multielectron OER/ORR, but also the evolution of superoxide/peroxide intermediates (such as O_2_^−^ and LiO_2_) and highly reactive radical chemistry. Among these processes, the generation of singlet oxygen (^1^O_2_) is widely regarded as one of the key factors responsible for electrolyte decomposition, carbon support corrosion, and rapid capacity fading. Because the formation of ^1^O_2_ is closely related to the spin states of oxygen intermediates, LOBs are theoretically particularly well suited for suppressing side reactions and delaying system failure through spin regulation [70].

#### 4.5.2 Major Research Pathways of Spin Catalysis in Nonaqueous LOBs

Research on spin catalysis in nonaqueous LOBs mainly follows two pathways:

i) CISS strategies. Traditional cathode catalysis research for LOBs has mainly focused on adsorption energies, reaction energy barriers, and interfacial structure regulation, whereas the recently emerging field of electron spin catalysis suggests that spin selectivity during electron transfer may constitute an independent kinetic regulation dimension beyond conventional thermodynamic and structural factors. Among these mechanisms, the CISS effect is considered one of the most representative mechanisms in current spin catalysis research [307, 308, 309].

Extensive studies have shown that in chiral molecules or chiral surfaces, electrons undergo pronounced spin polarization during transport, thereby altering the formation pathways and reaction rates of intermediates [310, 311, 312]. In oxygen electrocatalysis, spin selectivity has been demonstrated to reduce kinetic barriers for oxygen reduction or evolution reactions, regulate the formation probability of reaction intermediates, and thus influence reaction selectivity, while effectively suppressing excessive oxidation or radical‐related side reactions in certain systems. Although existing studies have mainly focused on aqueous ORR/OER systems, the core concept of enhancing electron transfer efficiency through spin matching also provides important insights for LOBs.

**FIGURE 15:**
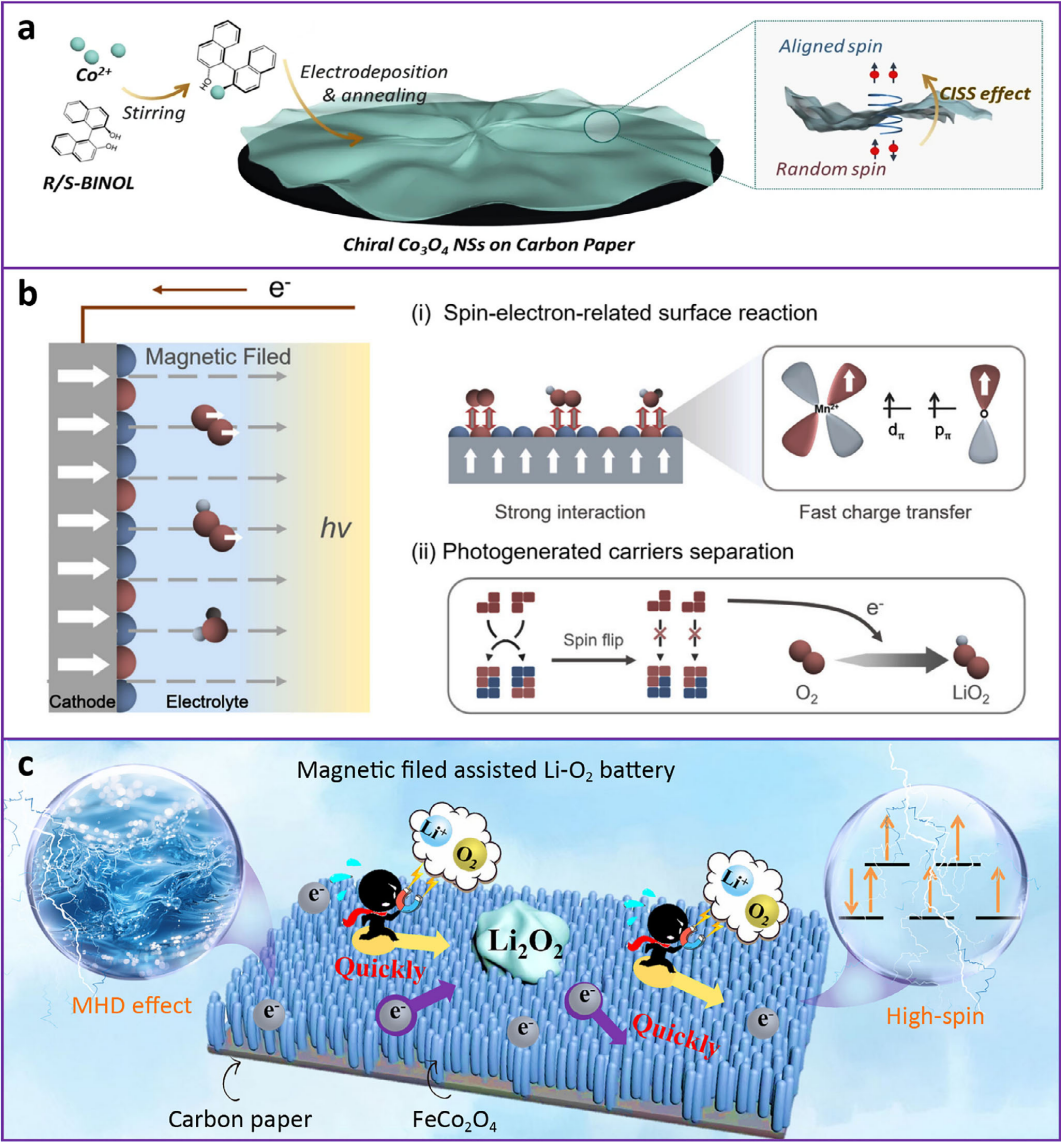
(a) Schematic illustration of the fabrication of chiral Co_3_O_4_ NS on CP. Reproduced with permission [316]. Copyright 2026, Springer Nature. (b) Illustration of enhanced spin‐polarization effect on LOBs by magnetic fields. Reproduced with permission [328]. Copyright 2025, Wiley. (c) Schematic diagram of spin regulation and magnetohydrodynamic effects driving novel magnetic field‐assisted LOBs. Reproduced with permission [329]. Copyright 2025, Elsevier.

In nonaqueous LOBs, the greatest advantage of the CISS strategy lies in its ability to regulate the spin‐state evolution of oxygen intermediates at the source, thereby suppressing the formation of harmful reactive oxygen species such as ^1^O_2_ [313, 314, 315]. Recent work has reported the use of chiral Co_3_O_4_ nanosheets as cathodes for LOBs, utilizing the CISS effect to suppress the generation of singlet oxygen (^1^O_2_), thereby mitigating parasitic reactions and extending cycle life (Figure 15a) [316]. The significance of this result lies in the fact that chiral cathodes can markedly suppress singlet oxygen generation, reduce parasitic reactions involving the electrolyte and carbon materials, and substantially prolong battery cycling stability.

More importantly, this finding demonstrates that spin regulation in LOBs is not merely reflected in lowering overpotentials or enhancing instantaneous activity, but rather in fundamentally weakening the most destructive side‐reaction mechanisms by altering the spin statistics of reaction pathways, thus providing a new strategy for improving long‐term stability.

ii) Magnetic materials and external magnetic field strategies. In addition to chiral systems, oxygen electrochemical reactions involving ferromagnetic materials or conducted under external magnetic fields have also shown significant performance enhancement [317]. Such activity enhancement is often attributed to the injection of spin‐polarized electrons, which makes reaction processes more consistent with spin angular momentum conservation requirements [318, 319, 320]. However, recent systematic studies indicate that activity improvements observed under magnetic fields are frequently accompanied by pronounced mass‐transfer enhancement effects, such as convection or bubble detachment induced by Lorentz forces [321, 322, 323, 324]. Therefore, when discussing spin catalysis mechanisms, it is essential to strictly distinguish intrinsic kinetic enhancement induced by spin polarization from mass‐transport or interfacial reconstruction effects triggered by magnetic fields [325, 326, 327]. This distinction is particularly critical for LOBs, which are inherently strongly limited by oxygen diffusion, Li^+^ transport, and pore blockage caused by discharge products.

Related studies have found that under external magnetic fields, charge‐discharge polarization in LOBs can be significantly reduced, while the formation and decomposition kinetics of Li_2_O_2_ are simultaneously enhanced (Figure 15b) [328]. This phenomenon is commonly explained by magnetic‐field‐induced electron spin polarization via Zeeman splitting or magnetic active centers, which alleviates spin restrictions during the initial electron transfer to oxygen molecules, and can be further combined with light irradiation to improve energy efficiency.

In nonaqueous LOBs, magnetic fields may also introduce magnetohydrodynamic effects, whereby the combined action of spin regulation and magnetohydrodynamics can improve oxygen diffusion and product mass transport (Figure 15c) [329]. At the same time, these studies emphasize that, beyond spin effects, the influence of magnetic fields on mass transport and convection must be considered, as oxygen diffusion, pore blockage, and product morphology strongly affect polarization in nonaqueous LOBs.

#### 4.5.3 Mechanistic Challenges and Characterization Limitations

Since LOBs are highly sensitive to mass transport, pore structure, and deposition morphology, mechanistic identification of electron spin effects in this system faces greater complexity than in aqueous systems. At present, spin‐catalysis‐induced changes in energy barriers are often small, yet their long‐term impact on cumulative side reactions and lifetime degradation may be decisive, making evaluations based solely on polarization voltage or initial capacity prone to underestimating their true value [330]. Meanwhile, the limited availability of operando spin‐resolved or intermediate characterization techniques in nonaqueous systems means that most conclusions still rely on indirect evidence.

To advance electron spin catalysis in nonaqueous LOBs from phenomenological validation to mechanistic consensus, future studies urgently need to introduce more rigorous and verifiable experimental frameworks. For example, paired comparisons between enantiomeric chiral materials and achiral controls, magnetic versus nonmagnetic cathodes with identical pore structures and surface areas, and the inclusion of singlet oxygen generation and electrolyte decomposition products as core evaluation metrics rather than focusing solely on cycle number or capacity retention are required. At the same time, combining experimental results with spin‐resolved theoretical calculations is expected to gradually clarify the key steps in which spin selectivity truly plays a role along the LOBs reaction pathway.

Electron spin catalysis has already demonstrated preliminary but clear application potential in nonaqueous LOBs, particularly in suppressing singlet‐oxygen‐related side reactions and improving reaction reversibility. Although this field is still at an early stage, the introduction of the spin degree of freedom is expected to become another important regulation dimension, following material composition, structural design, and interfacial engineering, to drive performance breakthroughs in LOBs.

## 5 HT Computation and AI‐Driven Design of Cathode Catalysts for LOBs

The performance of LOBs is constrained by the complex kinetics of the cathodic ORR and OER. The activity, stability, and dynamic structural evolution of cathode catalysts under operating conditions are key factors determining overall battery performance. However, owing to the structural complexity and large number of tunable parameters associated with catalytic systems such as oxides, metals, SACs, and heteroatom‐doped carbon materials, traditional experience‐driven material design approaches are often ineffective for efficient optimization within high‐dimensional chemical spaces. DFT simulations have laid an important foundation for battery material research [331, 332, 333, 334, 335, 336, 337], but with the exponential growth in the number of explorable materials, point‐by‐point screening based solely on DFT can no longer meet the contemporary demands of catalyst development in terms of efficiency, scale, and predictive capability.

The rapid development of HT computation, ML, and AI technologies has provided new pathways to overcome bottlenecks in materials design [338]. HT computation enables the automated generation of large‐scale structure‐property databases for catalysts, thereby establishing a foundation for data‐driven analysis [339]. ML techniques can extract structure‐activity relationships from these datasets, construct fast predictive models, and infer the performance of previously unexplored materials. Furthermore, with advances in AI in areas such as active learning, generative design, graph neural networks, and reinforcement learning, catalyst screening has evolved from passive prediction toward autonomous exploration and optimization. As a result, an intelligent design paradigm based on a closed loop of “computation‐learning‐experiment” is gradually taking shape.

### 5.1 HT and AI: From Configuration Compression to Generative Design

With the evolution of cathode catalysts in LOBs from simple metallic systems to multimetallic alloys, intermetallic compounds, single‐atom catalysts, and complex oxides with tunable compositions and defects, the compositional and structural space has expanded exponentially. Meanwhile, the structure‐performance relationship has become highly nonlinear and strongly coupled across multiple variables [340]. Under these circumstances, traditional design paradigms based on empirical screening and point‐by‐point DFT calculations face two fundamental challenges: (i) the difficulty of efficiently exploring the vast configurational space, and (ii) the prohibitive computational cost associated with large‐scale materials screening. Therefore, developing computational strategies that simultaneously ensure physical reliability and high exploration efficiency has become essential for the rational design of high‐performance cathode catalysts.

In recent years, the integration of HT, ML, and AI has provided a promising solution to these challenges. By introducing data‐driven models and automated workflows, catalyst discovery has gradually shifted from “exhaustive enumeration” to “strategic exploration”, forming a systematic framework that encompasses search space reduction, computational acceleration, complex system modeling, and experimental closed‐loop optimization.

#### 5.1.1 Search Space Reduction: Active Learning‐Driven HT Screening

**FIGURE 16:**
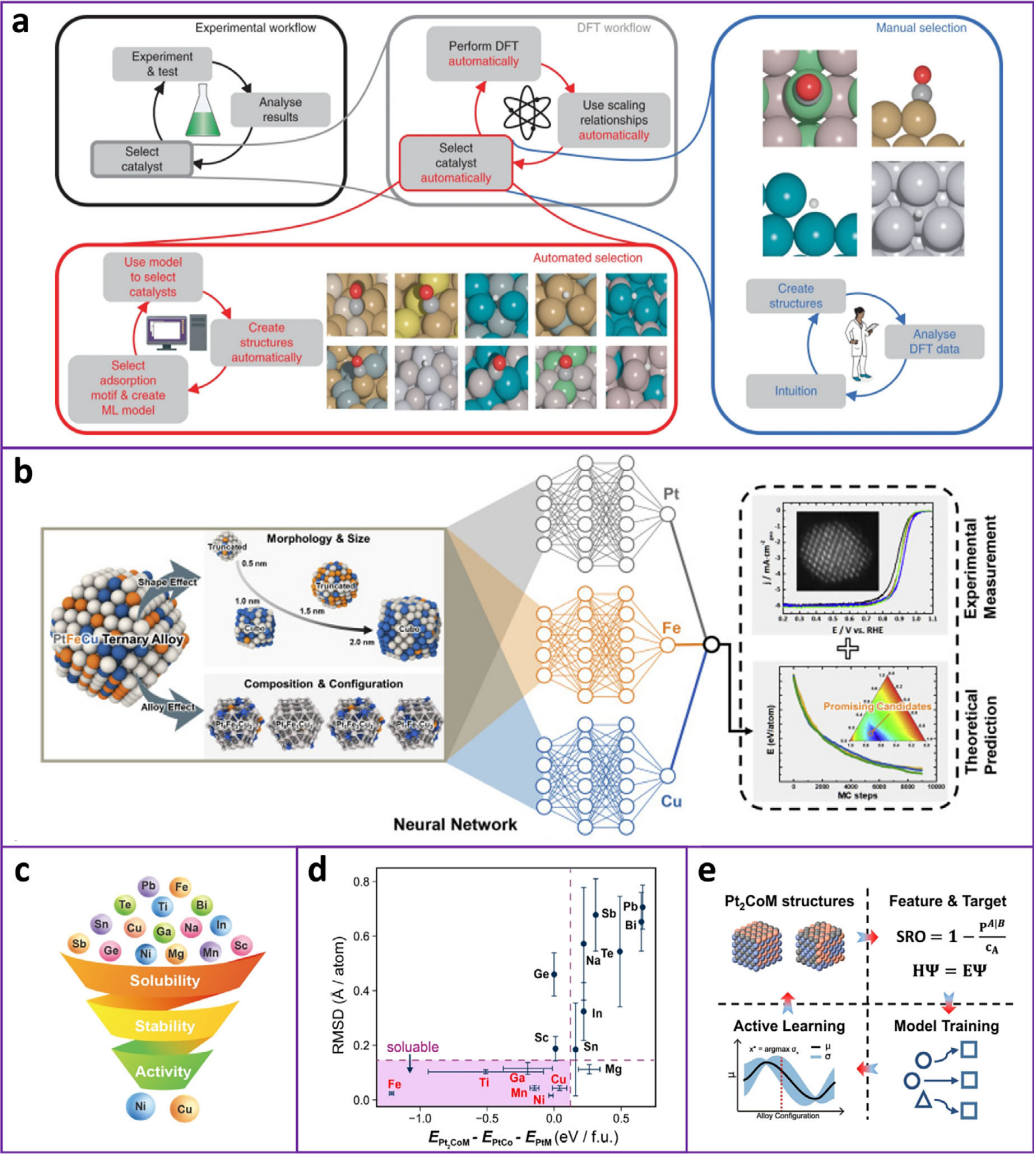
(a) Workflow for automating theoretical materials discovery. Reproduced with permission [341]. Copyright 2018, Springer Nature. (b) Schematic diagram of ternary alloy configuration search and theoretical predictions and experimental validation for ORR. Reproduced with permission [342]. Copyright 2021, Elsevier. (c‐e) Development of ML model. (c) Screening flowchart of ternary Pt_2_CoM combinations. (d) Structural deformation and relative energy distribution of Pt_2_CoM combinations. (e) Active learning procedures to construct an ML prediction model for relative energy of Pt_2_CoM. Reproduced with permission [343]. Copyright 2024, Springer Nature.

In high‐dimensional compositional and configurational spaces, the key to efficient exploration lies in minimizing redundant evaluations. To this end, active learning combined with high‐throughput computation has been widely adopted in catalyst screening (Figure 16a) [341]. In this framework, machine learning models are coupled with DFT calculations, enabling the model to dynamically select the most informative candidate structures for labeling based on prediction uncertainty. As a result, the training dataset is iteratively optimized, and the search space converges rapidly under a limited computational budget.

In practice, catalyst surfaces are represented by a set of quantifiable structural and electronic descriptors, including atomic identity, local coordination environment, electronegativity differences, and adsorption‐related features. These descriptors serve as inputs to machine learning models for predicting the adsorption behavior of key reaction intermediates on different surface sites. Through iterative “prediction‐selection‐validation” cycles, the model can efficiently identify promising candidates within large materials databases that satisfy optimal adsorption energy windows, significantly reducing the number of required first‐principles calculations.

Fundamentally, this approach transforms catalyst screening from uniform sampling to information‐driven sampling, enabling a shift from experience‐based manual decision‐making to self‐optimizing intelligent workflows. This strategy is particularly effective for narrowing down candidate spaces of multicomponent alloys and complex oxide catalysts in LOBs.

#### 5.1.2 Computational Acceleration: ML Potentials and High‐Dimensional Exploration

Although active learning effectively reduces the search space, the configurational complexity of multicomponent nanocatalysts still grows exponentially, making conventional DFT‐based enumeration computationally infeasible. To overcome this limitation, machine learning potentials (MLPs) have been introduced into catalyst screening.

By training neural network potentials on high‐quality first‐principles datasets, it becomes possible to achieve near‐DFT accuracy while reducing the computational cost of energy evaluations by several orders of magnitude. This enables large‐scale simulations of nanoparticle structures, surface reconstructions, and compositional segregation. When combined with high‐dimensional phase diagram exploration and statistical sampling techniques, MLPs allow for rapid identification of candidate structures that balance thermodynamic stability and catalytic activity (Figure 16b) [342].

Importantly, MLPs do not simply replace DFT; rather, they enable hierarchical allocation of computational resources, where DFT is reserved for critical configurations. This strategy significantly enhances overall screening efficiency and allows catalyst design to evolve from “computable” to “explorable”.

#### 5.1.3 Multidimensional Optimization in Complex Catalytic Systems

In practical systems, cathode catalysts in LOBs often involve multicomponent alloys, single‐atom sites, and complex oxides, where catalytic performance arises from the coupling of multiple structural variables such as size, composition, ordering, strain, and surface segregation. Such multidimensional interactions render traditional design strategies based on single descriptors or empirical rules insufficient.

ML methods have been employed to uncover hidden synergistic effects within high‐dimensional compositional‐structural spaces. Studies have shown that, in multimetallic alloys, the introduction of a third element can break existing coupling constraints among variables, enabling non‐intuitive optimal combinations of size, stability, and activity. This finding moves beyond the conventional binary tuning paradigm and highlights the importance of multicomponent synergy in catalyst design (Figure 16c–e) [343].

For single‐atom catalysts and multicomponent oxides, additional complexity arises from active site distribution, support stability, and reactant transport pathways. HT DFT combined with multi‐descriptor machine learning models incorporating adsorption free energies, electronic structure features, and orbital hybridization enables quantitative prediction of catalytic performance and offers mechanistic insights [344]. Furthermore, integrating limited experimental data with active learning strategies facilitates rapid identification of high‐performance candidates within complex compositional spaces [345, 346, 347].

These studies demonstrate that the key advantage of AI lies in its ability to identify non‐intuitive optimal regions under strongly coupled, high‐dimensional conditions, thereby overcoming the limitations of traditional single‐variable optimization strategies.

#### 5.1.4 From Screening to Generation: Data‐Driven Closed‐Loop Materials Discovery

**FIGURE 17:**
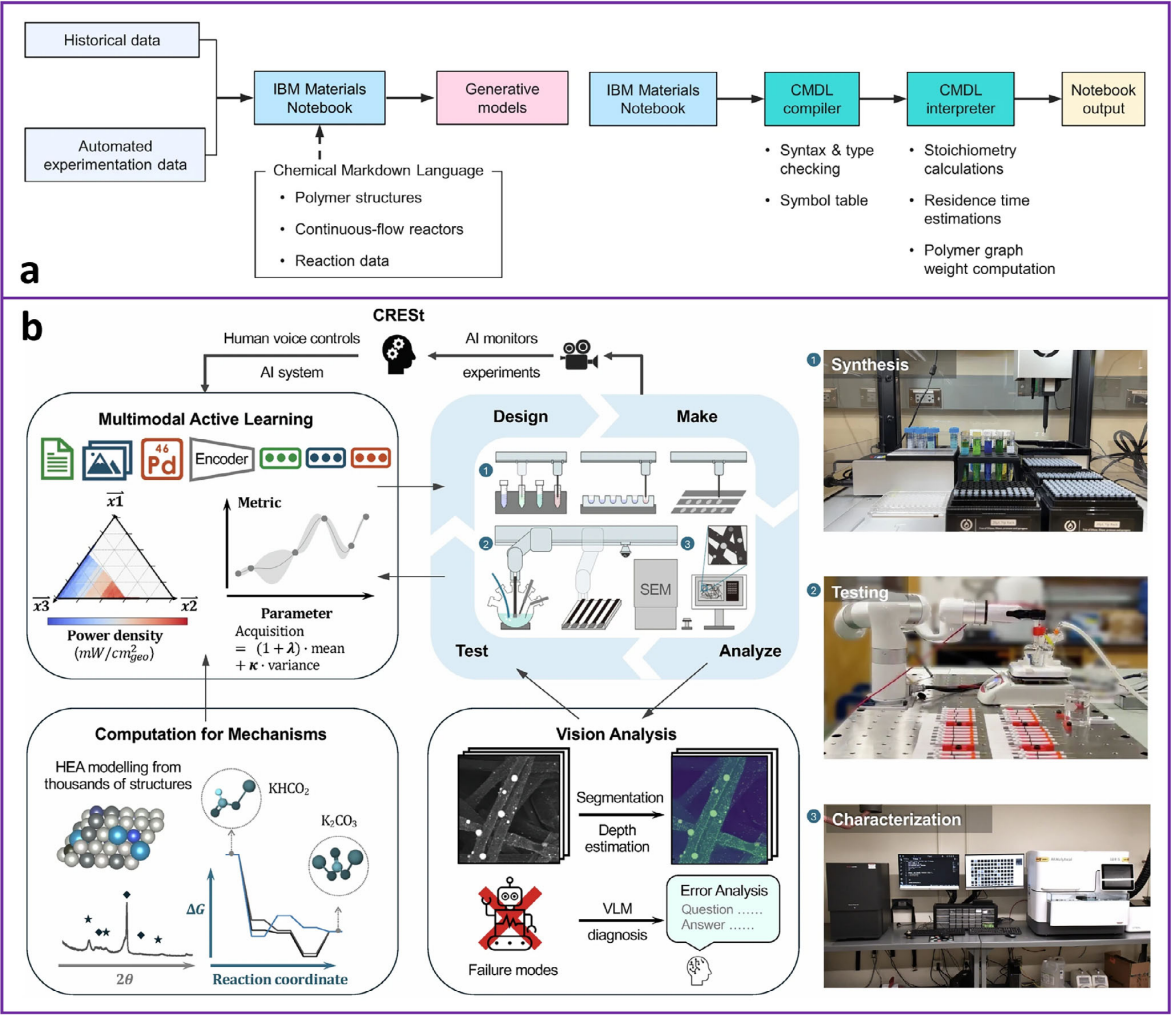
(a) Enabling development of improved ML for guiding discoveries in polymer science. Reproduced with permission [349]. Copyright 2023, Springer Nature. (b) The workflow of electrocatalyst discovery guided by CRESt. Reproduced with permission [350]. Copyright 2025, Springer Nature.

Beyond screening, AI‐driven materials design is increasingly shifting toward generative paradigms. By incorporating generative models, domain‐specific languages, and multimodal data integration, machine learning is no longer limited to property prediction but can actively participate in material structure design and synthesis planning [348]. For instance, a chemical markdown language (CMDL)‐based framework has been developed for generative design of catalysts and polymeric materials, demonstrating strong scalability and generality, particularly in ring‐opening polymerization (ROP) systems (Figure 17a) [349].

In parallel, the integration of automated experimental platforms and robotic systems has enabled the construction of closed‐loop frameworks that combine “generation‐synthesis‐testing‐feedback”. For example, the multimodal robotic platform CRESt (Copilot for Real‐world Experimental Scientists) integrates compositional, textual, and imaging data, and employs knowledge‐assisted Bayesian optimization to iteratively refine material design. This tightly coupled computational‐experimental workflow significantly accelerates catalyst discovery (Figure 17b) [350]. This evolution marks a fundamental shift in catalyst research from computation‐assisted screening to fully data‐driven autonomous discovery.

HT computation combined with AI is fundamentally transforming the design paradigm of cathode catalysts for LOBs. Through active learning‐based search space reduction, machine learning‐driven computational acceleration, multidimensional modeling of complex systems, and the integration of generative design with experimental feedback, materials discovery is evolving into a systematic and scalable framework [351].

However, these approaches primarily address the question of “which materials are promising”, while understanding “why they perform well” still requires deeper insights into the real structures and dynamic evolution of catalysts under operating conditions. In LOBs, catalyst composition, structure, and interfaces undergo multiscale transformations during operation, which critically influence reaction pathways and kinetics [352]. Therefore, advancing multiscale modeling of structural evolution and reaction mechanisms under realistic conditions will be essential for achieving truly rational catalyst design and performance optimization.

### 5.2 Multiscale Structural Modeling and Reaction‐Pathway Optimization

In LOBs, the performance of cathode catalysts is not determined solely by material composition, but rather by the combined effects of multiscale structural features and interfacial reaction processes [353]. From local coordination environments and electronic structure regulation at the atomic scale, to cluster size distributions, surface heterogeneity, and cooperative site effects at the nanoscale, and further to oxygen transport, electron/ion pathways, and discharge‐product deposition behavior within porous electrodes at the device scale, all of these factors can significantly influence the pathway selection, energy barrier distribution, and reversibility of the ORR and OER [354, 355, 356]. Therefore, as high‐throughput screening has gradually addressed the question of “which materials are worth investigating”, current research is increasingly shifting toward understanding “how structure determines reaction behavior” and “how to achieve synergistic regulation of structure, reaction, and mass transport across multiple scales”. In this transition, the role of artificial intelligence has also evolved from that of a simple performance prediction tool into an integrated framework linking structural characterization, reaction modeling, and mechanistic inference.

#### 5.2.1 Porous‐Network Modeling

At the device scale, the cathode in LOBs typically exhibits a highly complex porous network, in which pore‐size distribution, connectivity, tortuosity, and local geometric constraints collectively determine oxygen transport, ion diffusion, and the nucleation, growth, and clogging behavior of Li_2_O_2_ [357].

**FIGURE 18:**
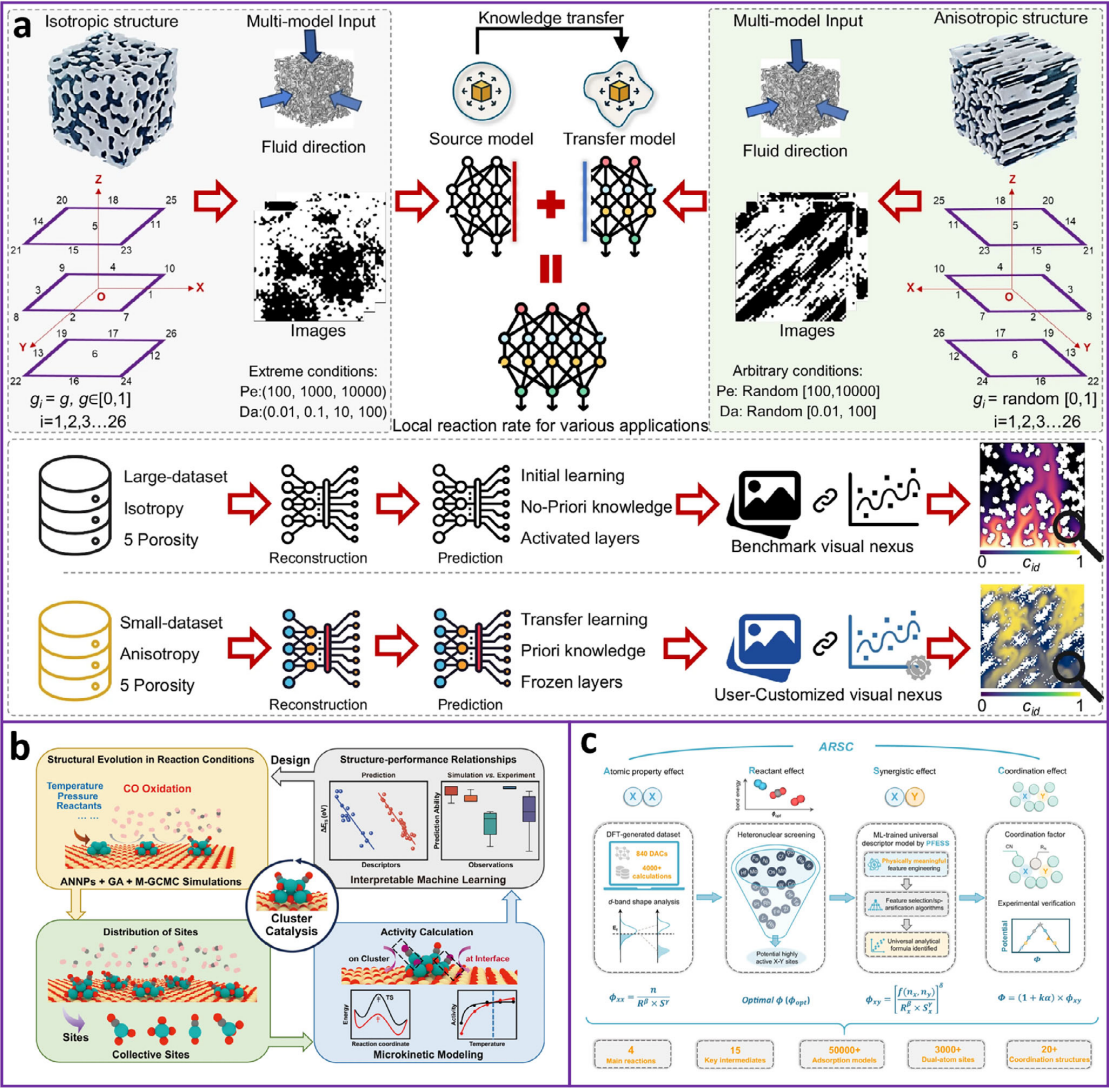
(a) Conditional Generative Adversarial Network and feature representation transfer learning (cGAN‐FRT) framework description. Reproduced with permission [358]. Copyright 2025, Springer Nature. (b) Workflow for studying cluster catalysis under reaction conditions. Reproduced with permission [362]. Copyright 2025, Springer Nature. (c) General workflow: (i) primitive descriptor (φ_xx_) for atomic property effects through d‐band shape analysis, (ii) screening principle (φ_opt_) of potentially desirable heteronuclear DACs based on reactant effects, (iii) ML‐based descriptors (φ_xy_) for synergistic effects through physically meaningful feature engineering based on φ_xx_ and feature selection/sparsification algorithms, (iv) the final universal descriptor model (Φ) with quantification of coordination effects and corresponding experimental verifications. Reproduced with permission [364]. Copyright 2024, Springer Nature.

Traditional studies have largely relied on two‐dimensional microscopic characterization or empirical structural parameters. Although these approaches can provide qualitative insights, they are insufficient to establish quantitative relationships between electrode geometry and local reaction behavior [358, 359]. In recent years, data‐driven structural reconstruction and reaction‐field modeling have provided a new route to address this challenge. By means of deep‐learning computer vision and data‐driven methods based on transfer learning (DLCV), three‐dimensional porous structures can be reconstructed from two‐dimensional experimental images, and then coupled with reaction kinetics and mass transport models to predict local concentration distributions, reaction‐rate fields, and the spatial evolution of deposited products (Figure 18a). In this way, porous electrodes are no longer merely objects of experimental characterization, but are transformed into computable and optimizable structural variables. This enables electrode design to be guided by objectives such as reducing concentration polarization, delaying pore blockage, and controlling deposition morphology, thereby improving rate capability and cycle stability at the device level.

#### 5.2.2 Collective Activity of Nanoclusters

At the nanoscale, the origin of catalytic activity in LOB catalysts also exhibits pronounced statistical and dynamic characteristics. Traditional catalytic theory often explains reaction behavior in terms of a single idealized active site or representative crystal facet. However, under real operating conditions, catalyst surfaces are typically composed of sites with different sizes, configurations, coordination environments, and coverage states, and these sites may play distinct roles at different potentials, intermediate coverages, and reaction stages [360]. In particular, during the formation and decomposition of Li_2_O_2_, the interface itself is continuously evolving, such that catalytic activity no longer corresponds to one fixed optimal site, but is more appropriately understood as an emergent “site community” effect arising from the collective contributions of multiple types of sites [361]. By performing rapid energy evaluation and reaction‐parameter estimation over large structural ensembles and combining these with kinetic models, it becomes possible to quantify the contribution weights of different cluster sizes, isomers, and site types to the overall activity, and to identify the effective site density, adsorption‐energy distribution, and their dynamic evolution that govern the overall performance (Figure 18b) [362]. The significance of this perspective lies in the fact that it shifts catalyst design from “searching for the optimal site” toward “constructing a site ensemble that maintains a high density of effective sites and a rational functional distribution under operating conditions”, which is more consistent with the actual characteristics of complex interfacial reactions in LOBs [363].

#### 5.2.3 Interpretable Atomic‐Level Cooperative Design

At the atomic scale, artificial intelligence is further promoting catalyst design from empirical correlation‐based prediction toward interpretable mechanistic modeling. Although deep‐learning models have demonstrated strong capabilities in catalytic activity prediction, their “black‐box” nature often limits the verifiability of the results and their transferability across different systems. For LOBs, simply knowing that a certain structure is “predicted to be more active” is far from sufficient. More importantly, one needs to understand which local electronic‐structure features favor the stabilization of key intermediates, which coordination environments help lower the energy barriers of key OER steps, and which structural factors may induce side reactions or irreversible deactivation. Accordingly, a more promising recent direction has been to embed interpretability into atomic‐scale design.

One example is an interpretable dual‐atom‐site design strategy, termed Activity‐Reactant‐Synergy‐Coordination (ARSC), which constructs an interpretable descriptor system linking atomic properties, coordination environments, reactant coupling, and cooperative site effects, thereby connecting “site geometry‐electronic structure‐adsorption behavior‐reaction selectivity” within a unified framework and enabling rapid prediction and candidate screening with limited DFT support (Figure 18c) [364]. Particularly in the design of dual‐atom or multi‐atom cooperative sites, such methods can transform synergy effects from empirical concepts into quantifiable variables, such as synergy strength and coordination‐driven trends in d‐band regulation, thereby enabling controllable optimization toward target reactions, including ORR/OER enhancement or side‐reaction suppression [365]. For the rational development of cathode catalysts for LOBs, the value of these interpretable models lies not only in improving screening efficiency, but also in providing a clearer theoretical basis for designing active sites that are synthesizable, stable, and functional under real operating conditions [366].

#### 5.2.4 Intelligent Mechanistic Analysis

However, structural description and active‐site analysis alone are still insufficient to fully explain the electrochemical behavior of LOBs, because their performance is ultimately governed by pathway competition and kinetic control within complex reaction networks [367]. During ORR/OER, multiple adsorbed intermediates, surface species, and deposited products often coexist, forming a highly coupled reaction space under conditions of high coverage, strong interactions, and dynamic interfacial reconstruction [368]. Although traditional DFT‐based mechanistic studies have been highly valuable for elucidating individual reaction steps or low‐coverage systems, they are often constrained in the face of high‐dimensional reaction networks by the vast configurational space, the high computational cost of pathway searching, and the strong dependence on initial assumptions [369].

**FIGURE 19:**
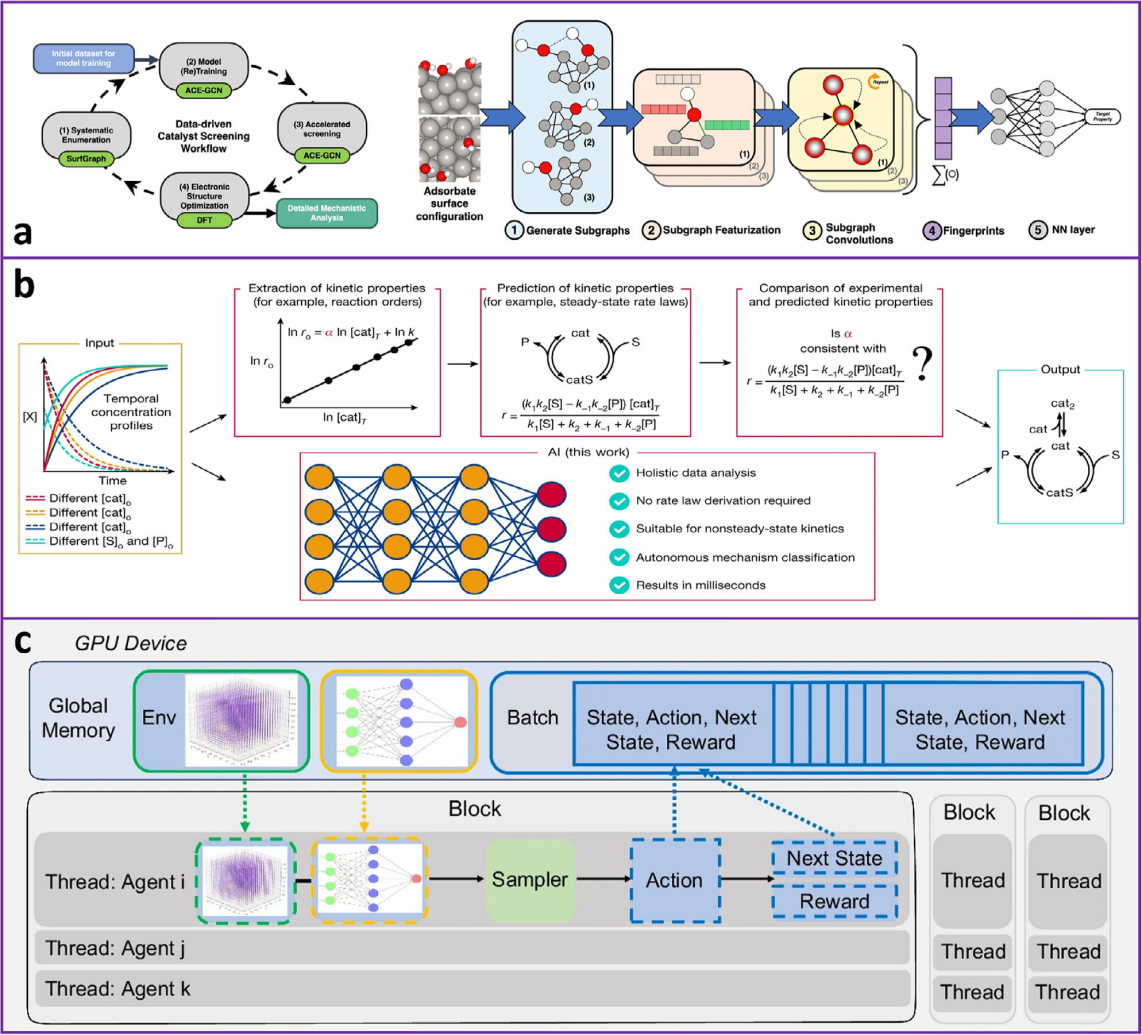
(a) Catalyst screening workflow and overview of the ACE‐GCN algorithm. Reproduced with permission [370]. Copyright 2022, Springer Nature. (b) Comparison of current pipeline for mechanistic elucidation through kinetic analysis versus the use of our new, AI‐based mechanistic elucidation. Reproduced with permission [371]. Copyright 2023, Springer Nature. (c) A flow chart depicting the first‐principles‐instructed, HT deep reinforcement learning framework used in the study of the hydrogenation reaction in ammonia synthesis on the Fe (1 1 1) surface. Reproduced with permission [372]. Copyright 2024, Springer Nature.

The introduction of artificial intelligence is driving mechanistic studies from “local pathway analysis” toward “global reaction‐network modeling”. On one hand, researchers have proposed an Adsorbate Chemical Environment‐based Graph Convolution Neural Network (ACE‐GCN) framework (Figure 19a) [370]. This graph‐neural‐network‐based modeling strategy represents adsorbates, surface sites, and their interactions within a unified graph structure, thereby effectively capturing many‐body effects under high‐coverage conditions and rapidly identifying stable configurations and potential deactivation environments in complex adsorption‐state spaces. On the other hand, deep learning has been used to directly identify reaction‐mechanism types from kinetic data. By training on large numbers of simulated and experimental reaction curves, neural networks can learn the “kinetic fingerprints” of different mechanisms in time‐ or potential‐dependent responses and classify reaction processes without the need to manually specify a mechanistic model. Such methods can maintain high identification accuracy even when data are noisy, sparsely sampled, or under non‐steady‐state conditions, demonstrating strong robustness (Figure 19b) [371].

In addition, a reaction‐pathway search framework combining reinforcement learning with first‐principles calculations has also been proposed. In this high‐throughput deep reinforcement learning framework with first principles (HDRL‐FP), atomic structures are mapped onto potential energy surfaces defined by first‐principles calculations, and the reinforcement‐learning agent autonomously searches for the lowest‐energy‐barrier reaction pathways through continuous trial, feedback, and policy updating, thereby overcoming the dependence of conventional transition‐state searches on manually predefined pathways (Figure 19c) [372]. For LOBs, these advances imply that reaction mechanisms are no longer merely analytical tools used a posteriori to interpret experimental phenomena, but are beginning to become design objects that can be directly modeled, identified, and optimized.

For LOBs, such self‐learning reaction‐pathway exploration is particularly valuable. By sampling catalyst‐surface potential energy surfaces via reinforcement learning, models can identify minimum‐barrier pathways. They can also automatically locate key intermediates and rate‐determining steps [369]. This provides quantitative guidance for reaction‐pathway optimization of bifunctional ORR/OER catalysts. When further integrated with high‐coverage adsorption models and mechanistic classification results, a continuously self‐optimizing reaction‐network exploration framework becomes feasible.

AI is fundamentally reshaping the paradigm of reaction‐mechanism research in LOBs. Graph neural networks render high‐coverage, multi‐intermediate adsorption systems computationally tractable. Deep‐learning‐based mechanism classification enables experimental data to directly reflect reaction‐pathway characteristics. Reinforcement learning offers new tools for automatic exploration and optimization of high‐dimensional reaction‐pathway spaces. Together, these approaches are driving mechanistic analysis from hypothesis‐driven local studies toward data‐driven global reaction‐network modeling [373]. Within this new paradigm, reaction mechanisms are no longer merely post hoc explanations of performance. Instead, they become explicit design variables that can be directly optimized. By unifying mechanistic analysis, structural modeling, and materials screening within a single AI‐driven framework, a truly closed‐loop design strategy becomes possible. Such integration provides systematic theoretical support for developing highly efficient and reversible cathode catalysts for LOBs.

### 5.3 Challenges and Perspectives: Toward Interpretable, Reliable, and Generalizable Mechanistic AI

Although ML and AI have demonstrated significant advantages in HT catalyst screening and performance prediction, enabling automated exploration of large chemical spaces, their practical application in systems as complex and strongly coupled as LOBs still faces a series of critical challenges [374]. These challenges arise not merely from algorithmic performance limitations, but more fundamentally from issues related to model assumptions, physical consistency, experimental verifiability, and cross‐system generalizability.

#### 5.3.1 The Gap Between High Predictive Performance and Experimental Feasibility

Current AI‐driven screening workflows often optimize adsorption energies, reaction free energies, or theoretical overpotentials as primary objectives. However, in practical battery systems, catalyst feasibility is additionally constrained by thermal stability, electrochemical stability, adsorption‐induced surface segregation, structural reconstruction, noble‐metal cost, and synthetic accessibility. Neglecting these factors can easily lead to screening outcomes that are theoretically optimal but experimentally impractical.

Future catalyst‐screening frameworks must therefore evolve from single‐metric optimization toward multi‐objective, strongly constrained decision problems [375]. At the model level, stability, structural evolution tendencies, and process feasibility constraints should be incorporated simultaneously. This integration would enable AI‐generated candidate materials to more closely approximate realistically achievable systems. Such progress also requires a tighter iterative feedback loop between computational models and experimental validation.

#### 5.3.2 Systematic Management of Uncertainty Quantification and Error Propagation

Uncertainty in ML‐based catalytic predictions arises from multiple sources. These sources include exchange‐correlation functional approximations in DFT, biases in assumed reaction mechanisms, breakdown of scaling relations, and intrinsic regression errors of the models themselves. If such uncertainties are not explicitly quantified, model predictions cannot reliably serve as decision‐making tools [376]. Consequently, a central future direction for mechanistic AI is to elevate uncertainty quantification from an auxiliary feature to an intrinsic component of model design. Through Bayesian learning, ensemble modeling, or active learning strategies, prediction confidence intervals can be explicitly defined. These uncertainties can then be directly propagated into screening and pathway‐optimization decisions. This enables the development of risk‐aware intelligent design frameworks.

#### 5.3.3 From “Black‐Box Prediction” to Physically Interpretable ML

Existing deep‐learning models in complex catalytic systems often exhibit high accuracy but low interpretability. Such “black‐box” behavior limits the transferability of model conclusions to new systems [377]. It also restricts the ability of AI models to contribute back to fundamental catalytic theory.

A more sustainable direction is the development of mechanistic AI with embedded physical constraints and interpretability. This includes explicitly incorporating conservation laws, chemical priors, symmetry constraints, and interpretable descriptors into model architectures. In this way, models can not only predict what performs well, but also explain why it performs well and how it can be tuned. In LOBs, such interpretability is particularly critical for understanding interconversions among key ORR/OER intermediates such as *O_2_^−^, LiO_2_, Li_2_O_2_, and LiOH.

#### 5.3.4 Unified Representation of High‐Dimensional Active Sites and Dynamic Reconstruction

Future industrial‐scale LOBs impose more stringent requirements on cathode catalysts. Beyond high activity, catalysts must maintain long‐term stability under complex operating conditions [378]. This necessitates upgrading active‐site descriptions from static, low‐dimensional features to high‐dimensional representations. Such representations must simultaneously capture electronic structure, interfacial potentials, solvent effects, and dynamic reconstruction behavior [379]. In this context, the key mission of mechanistic AI is to unify atomic‐scale electronic structure, mesoscopic structural evolution, and macroscopic reaction behavior within a single modeling framework. Under such conditions, active sites are no longer treated as fixed structures. Instead, they become state variables that evolve with reaction conditions. This perspective more faithfully reflects the true working mechanisms of LOBs.

#### 5.3.5 Computation‐Experiment‐Learning Closed‐Loop and Mechanism‐Driven Design Paradigm

As mechanistic AI frameworks mature, their application is expanding from idealized model systems to metal oxides, SACs, and multiphase interfacial reactions unique to LOBs. Compared with traditional experience‐driven approaches, mechanistic AI can learn deep mappings between catalyst electronic structure, intermediate adsorption, and reaction pathways [380]. This capability enables direct prediction of reaction bottlenecks and optimal pathways.

More importantly, when mechanistic AI is deeply integrated with HT computation, operando or online experimental characterization, and automated experimental platforms, a truly self‐iterative computation‐experiment‐learning closed loop can be established. Within this loop, models continuously refine mechanistic hypotheses based on experimental feedback [381, 382]. Through iteration, they gradually converge toward optimal structures and reaction pathways. This paradigm not only compensates for limitations of traditional catalytic theory in multistep and multiphase interfacial systems. It also provides a clear developmental blueprint for the intelligent design of next‐generation high‐performance LOBs cathode catalysts.

The role of AI in future LOBs catalyst research will evolve from an efficient screening tool to a core engine for mechanistic understanding and pathway design. Only when models achieve interpretability, reliability, and cross‐system generalizability can AI truly become an integral part of catalytic science rather than merely a computational accelerator. Mechanistic AI research oriented toward these goals will be a key driving force in advancing LOBs from material discovery toward controllable performance and mechanism‐guided design.

## 6 Summary

Rechargeable nonaqueous LOBs are regarded as one of the most promising candidates for surpassing the performance limits of existing energy storage systems owing to their ultrahigh theoretical energy density. However, their practical application is still hindered by a series of critical challenges, including sluggish cathode reaction kinetics, irreversible accumulation of discharge products, severe side reactions between electrolytes and carbon supports, and limited cycling life. Among these issues, cathode catalysts play a central role in regulating ORR/OER pathways, stabilizing reaction interfaces, and suppressing detrimental side reactions, and therefore have remained a long‐term research focus in this field.

This review systematically summarizes the research progress and development landscape of cathode catalyst materials for nonaqueous LOBs. First, starting from the historical development, fundamental working principles, and key bottlenecks of LOBs, the necessity and complexity of designing high‐performance cathode catalysts are elucidated. Subsequently, focusing on conventional cathode catalytic systems, representative advances of carbon‐based catalysts, noble‐metal‐based catalysts, TM oxides, sulfides/nitrides/carbides, and RMs in enhancing reaction kinetics and regulating discharge‐product behavior are systematically summarized. The trade‐offs among catalytic activity, structural stability, and material cost associated with these different catalyst classes are also critically analyzed.

**FIGURE 20:**
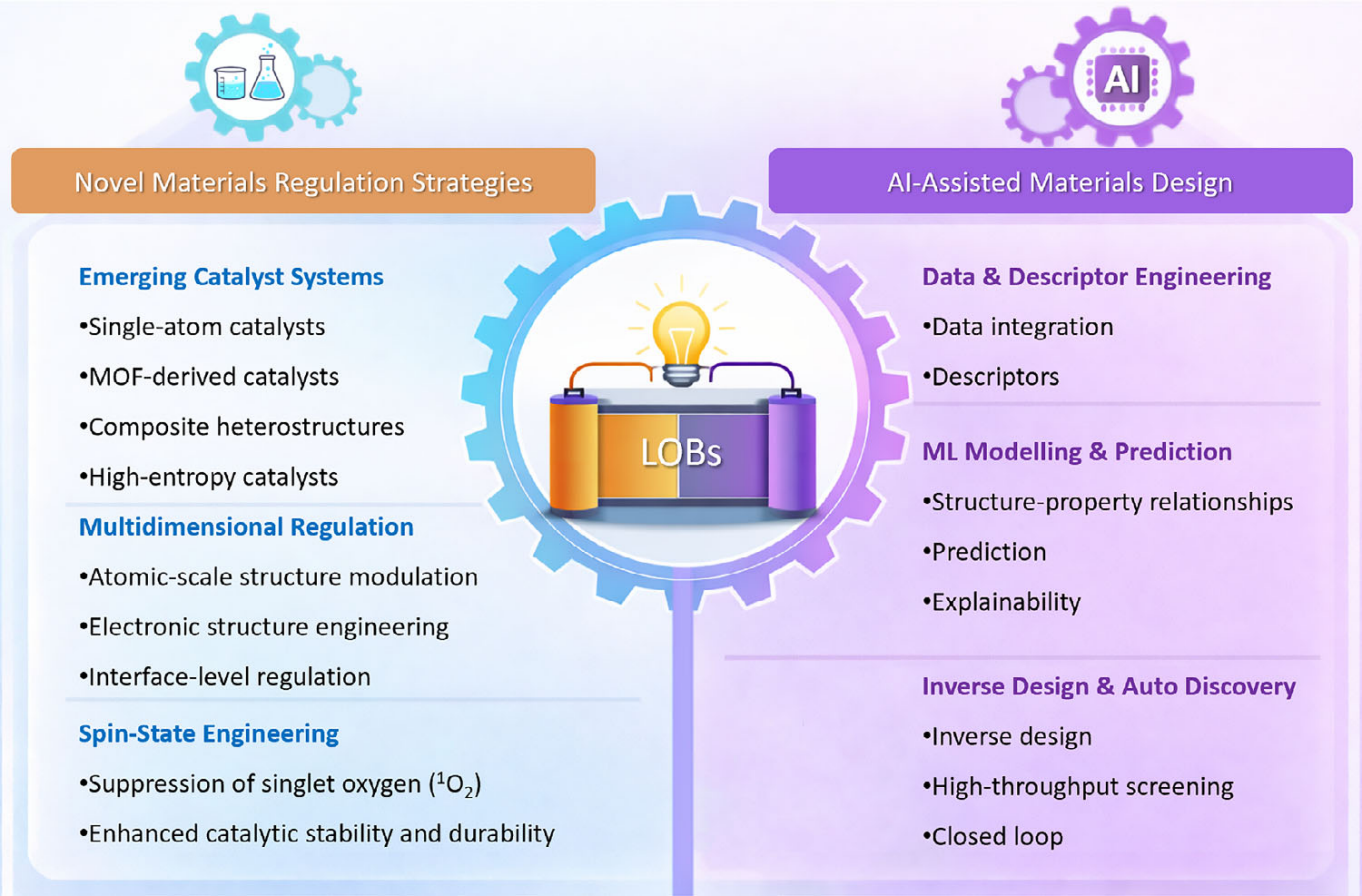
Forward‐looking research avenues for catalysts in LOBs.

On this basis, emerging catalytic systems and advanced design strategies developed in recent years are highlighted, including SACs, MOF‐derived catalysts and in situ transformation strategies, composite heterostructures and interfacial synergistic effects, as well as high‐entropy catalysts. These strategies introduce higher‐dimensional degrees of freedom at the atomic, electronic, and interfacial levels, offering new opportunities to overcome the intrinsic performance limitations of conventional catalysts. Meanwhile, this review further emphasizes the unique potential of spin‐regulated oxygen electrocatalysis in nonaqueous LOBs, particularly its long‐term advantages in suppressing harmful reactive oxygen species such as singlet oxygen and mitigating system degradation (Figure 20).

In addition, with the rapid development of HT computation, AI, and ML techniques, catalyst design is gradually shifting from experience‐driven to data‐ and model‐driven paradigms. By integrating atomic‐scale characterization with multiscale modeling, a deeper understanding of true active sites and reaction mechanisms can be achieved. Furthermore, material design strategies based on HT screening and intelligent algorithms are expected to significantly accelerate the discovery and optimization of high‐performance cathode catalysts.

Overall, research on cathode catalysts for nonaqueous LOBs is evolving toward multidimensional synergistic regulation, mechanism‐oriented design, and cross‐scale integrated optimization. Although challenges such as complex reaction mechanisms, nonuniform evaluation criteria, and decoupling between experiments and theory still persist, continuous advances in characterization techniques, theoretical models, and intelligent design tools are gradually enabling rational catalyst design and performance breakthroughs. It can be anticipated that, through the synergistic integration of material composition, structural engineering, interfacial regulation, and electronic degrees of freedom such as spin, nonaqueous LOBs will achieve substantial improvements in energy density, cycling stability, and practical feasibility, thereby laying a solid foundation for the development of next‐generation high‐energy‐density energy storage technologies.

## Funding

Q.S. acknowledges financial support from the European Union's Horizon Europe Research and Innovation Programme under the Marie Skłodowska‐Curie Grant Agreement No. 101211154. This work was supported by the National Key R&D Program of China (Grant No. 2025YFE0125100), the National Natural Science Foundation of China (Grant Nos. 52475336 and 52125505), the MAIAMI project (Grant No. AIA2025‐164488‐C22) funded by MICIU/AEI/10.13039/501100011033, the State Key Laboratory of Precision Welding & Joining of Materials and Structures (Grant No. MSWJ‐25M‐04), and the Generalitat de Catalunya (Grant No. 2021SGR01581).

## Conflicts of Interest

The authors declare no conflicts of interest.

## Data Availability

Data sharing not applicable to this article as no datasets were generated or analysed during the current study.
